# Supplementary material for: How has the emergence of the Omicron SARS-CoV-2 variant of concern influenced worry, perceived risk and behaviour in the UK? A series of cross-sectional surveys
Source: BMJ Open. 2022 Aug 29;12(8):e061203. doi: 10.1136/bmjopen-2022-061203 (PMC9437738; doi:10.1136/bmjopen-2022-061203)
Supplement: Supplementary data [file bmjopen-2022-061203supp001.pdf]

Supplementary materials

Figure 1. Timeline of announcements, data collection, and dates of self-reported behaviours. All dates 2021.

<table>
<tr><td>22-Nov</td><td></td><td></td><td rowspan="11">Wave 63 “past seven days” could cover</td><td></td><td></td></tr>
<tr><td>23-Nov</td><td></td><td></td><td></td><td></td></tr>
<tr><td>24-Nov</td><td>Omicron variant reported to WHO</td><td></td><td></td><td></td></tr>
<tr><td>25-Nov</td><td></td><td></td><td></td><td></td></tr>
<tr><td>26-Nov</td><td>Omicron designated a variant of concern</td><td></td><td></td><td></td></tr>
<tr><td>27-Nov</td><td>New measures announced
First UK cases</td><td></td><td></td><td></td></tr>
<tr><td>28-Nov</td><td></td><td></td><td></td><td></td></tr>
<tr><td>29-Nov</td><td></td><td rowspan="4">Wave 63</td><td rowspan="10">Wave 63.5 “past seven days” could cover</td><td></td></tr>
<tr><td>30-Nov</td><td>New measures take effect
Daily Mirror breaks ‘Partygate’</td><td></td></tr>
<tr><td>01-Dec</td><td></td><td></td></tr>
<tr><td>02-Dec</td><td></td><td></td></tr>
<tr><td>03-Dec</td><td>Omicron cases in the UK exceed 100</td><td></td><td></td><td></td></tr>
<tr><td>04-Dec</td><td></td><td></td><td></td><td></td></tr>
<tr><td>05-Dec</td><td></td><td></td><td></td><td></td></tr>
<tr><td>06-Dec</td><td></td><td rowspan="3">Wave 63.5</td><td></td><td rowspan="8">Wave 64 “past seven days” could cover</td></tr>
<tr><td>07-Dec</td><td>ITV reports on a video seeming to confirm ‘Partygate’</td><td></td></tr>
<tr><td>08-Dec</td><td>“Plan B” announced</td><td></td></tr>
<tr><td>09-Dec</td><td></td><td></td><td></td><td></td></tr>
<tr><td>10-Dec</td><td></td><td></td><td></td><td></td></tr>
<tr><td>11-Dec</td><td></td><td></td><td></td><td></td></tr>
<tr><td>12-Dec</td><td></td><td></td><td></td><td></td></tr>
<tr><td>13-Dec</td><td>“Plan B” mostly takes effect
First UK death from Omicron</td><td>Wave 64</td><td></td><td></td></tr>
</table>

14-Dec					
15-Dec	UK records its highest number of daily cases (78,610)				
16-Dec	UK records its highest number of daily cases (88,376)				

Table 1. Associations between out-of-home activities and socio-demographic factors, adjusting for all other socio-demographic factors. Bolding denotes significant findings (p<.002).

		Going out shopping (for groceries/pharmacy and other items) [block 1]				Attending the workplace [block 1]			
Attribute	Level	Wave 63.5 [a] aIRR for going out shopping (95% CI)	*p*	Wave 64 [b] aIRR for going out shopping (95% CI)	*p*	Wave 63.5 [c] aIRR for attending the workplace (95% CI)	*p*	Wave 64 [d] aIRR for attending the workplace (95% CI)	*p*
Region	East Midlands	Ref	-	Ref	-	Ref	-	Ref	-
	East of England	0.93 (0.72 to 1.21)	0.59	0.90 (0.70 to 1.15)	0.40	1.07 (0.53 to 2.16)	0.84	0.94 (0.52 to 1.70)	0.83
	London	1.10 (0.84 to 1.44)	0.48	1.09 (0.86 to 1.39)	0.46	0.93 (0.51 to 1.69)	0.81	1.15 (0.69 to 1.92)	0.60
	North East	1.01 (0.71 to 1.45)	0.94	0.96 (0.71 to 1.31)	0.81	0.83 (0.38 to 1.84)	0.65	1.07 (0.49 to 2.34)	0.86
	North West	1.08 (0.83 to 1.42)	0.57	1.06 (0.84 to 1.33)	0.63	1.17 (0.62 to 2.18)	0.63	0.91 (0.53 to 1.58)	0.75
	South East	0.99 (0.77 to 1.27)	0.93	0.94 (0.75 to 1.17)	0.57	0.96 (0.51 to 1.82)	0.90	1.14 (0.67 to 1.97)	0.63
	South West	0.98 (0.75 to 1.30)	0.91	1.05 (0.82 to 1.35)	0.68	1.50 (0.79 to 2.84)	0.22	1.54 (0.81 to 2.91)	0.18
	West Midlands	1.13 (0.86 to 1.49)	0.37	1.02 (0.80 to 1.31)	0.86	1.32 (0.71 to 2.46)	0.38	0.78 (0.44 to 1.39)	0.41
	Yorkshire and The Humber	1.37 (0.99 to 1.90)	0.06	0.89 (0.70 to 1.13)	0.33	1.24 (0.53 to 2.88)	0.62	0.67 (0.35 to 1.31)	0.24
	Overall	$\chi^2(8)=9.3$	0.32	$\chi^2(8)=6.6$	0.58	$\chi^2(8)=5.9$	0.66	$\chi^2(8)=7.9$	0.44
Sex	Male	Ref	-	Ref	-	Ref	-	Ref	-
	Female	0.83 (0.74 to 0.94)	0.002	0.94 (0.84 to 1.05)	0.25	0.72 (0.54 to 0.96)	0.03	0.72 (0.54 to 0.95)	0.02
Age (per decade)	Raw age	0.95 (0.91 to 0.99)	0.02	0.98 (0.94 to 1.01)	0.22	0.78 (0.65 to 0.93)	0.007	0.89 (0.77 to 1.03)	0.13
Age: quadratic $(\text{age-mean})^2$	-	1.0000 (0.9998 to 1.0002)	0.99	0.9999 (0.9997 to 1.0001)	0.37	0.9995 (0.9987 to 1.0003)	0.23	0.9997 (0.9990 to 1.0005)	0.48
Dependent child in household	None	Ref	-	Ref	-	Ref	-	Ref	-
	Child present	1.07 (0.93 to 1.23)	0.34	1.15 (1.01 to 1.31)	0.04	1.33 (0.98 to 1.81)	0.07	1.21 (0.91 to 1.61)	0.20
At high risk (self)	No	Ref	-	Ref	-	Ref	-	Ref	-
	Yes	0.88 (0.75 to 1.03)	0.10	0.96 (0.83 to 1.10)	0.53	0.99 (0.69 to 1.41)	0.94	0.83 (0.57 to 1.22)	0.35
Household member has chronic illness	No	Ref	-	Ref	-	Ref	-	Ref	-
	Yes	0.95 (0.80 to 1.13)	0.57	1.14 (0.98 to 1.33)	0.08	1.12 (0.68 to 1.84)	0.66	0.97 (0.61 to 1.55)	0.90
Employment status	Not working	Ref	-	Ref	-	-	-	-	-
	Working	1.07 (0.92 to 1.23)	0.37	0.97 (0.85 to 1.11)	0.65	-	-	-	-
Socio-economic grade	ABC1	Ref	-	Ref	-	Ref	-	Ref	-
	C2DE	1.06 (0.93 to 1.20)	0.38	0.98 (0.87 to 1.10)	0.74	0.70 (0.51 to 0.96)	0.03	0.85 (0.61 to 1.18)	0.32
Index of multiple deprivation	1st (least) to 4th quartile (most deprived)	0.95 (0.90 to 1.01)	0.10	0.95 (0.89 to 1.00)	0.05	0.97 (0.84 to 1.12)	0.71	0.84 (0.73 to 0.97)	0.02
Highest educational or professional qualification	Less than degree	Ref	-	Ref	-	Ref	-	Ref	-
	Degree or higher	0.96 (0.84 to 1.10)	0.54	1.02 (0.90 to 1.16)	0.74	0.83 (0.63 to 1.11)	0.22	0.64 (0.47 to 0.87)	0.004
Ethnicity	White British	Ref	-	Ref	-	Ref	-	Ref	-
	White other	1.21 (0.88 to 1.64)	0.24	0.76 (0.55 to 1.06)	0.11	1.36 (0.69 to 2.68)	0.38	0.97 (0.51 to 1.84)	0.92
	Black and minority ethnicity	0.87 (0.71 to 1.07)	0.20	1.06 (0.88 to 1.27)	0.55	1.03 (0.70 to 1.49)	0.90	0.98 (0.67 to 1.44)	0.92
	Overall	$\chi^2(2)=4.3$	0.12	$\chi^2(2)=3.9$	0.14	$\chi^2(2)=0.8$	0.68	$\chi^2(2)=0.0$	0.99

First language	Not English	Ref	-	Ref	-	Ref	-	Ref	-
	English	0.72 (0.55 to 0.93)	0.013	0.71 (0.55 to 0.92)	0.01	1.23 (0.74 to 2.04)	0.42	0.68 (0.41 to 1.12)	0.13
Ever had COVID-19	Think not	Ref	-	Ref	-	Ref	-	Ref	-
	Think yes	1.17 (1.02 to 1.34)	0.03	0.95 (0.84 to 1.09)	0.48	0.92 (0.68 to 1.25)	0.60	0.96 (0.71 to 1.30)	0.80
Vaccination status	Not vaccinated	Ref	-	Ref	-	Ref	-	Ref	-
	1 dose	1.19 (0.89 to 1.57)	0.24	1.16 (0.89 to 1.51)	0.27	1.37 (0.81 to 2.32)	0.24	1.47 (0.87 to 2.47)	0.15
	2 doses or more	0.98 (0.82 to 1.18)	0.85	0.80 (0.68 to 0.96)	0.01	0.95 (0.64 to 1.40)	0.78	1.10 (0.76 to 1.60)	0.62
	Overall	$\chi^2(2)=2.2$	0.34	$\chi^2(2)=12.5$	0.002	$\chi^2(2)=2.6$	0.27	$\chi^2(2)=2.1$	0.34
Financial hardship	Range 3 (least) to 15 (most)	1.01 (0.99 to 1.03)	0.21	**1.03 (1.02 to 1.05)**	**<0.001**	1.02 (0.97 to 1.07)	0.40	1.06 (1.01 to 1.10)	0.02

a) 1622 people were eligible for inclusion in analyses investigating going out shopping analyses. There were different amounts of missing data depending on variables included in the models, so n ranged between 1440 and 1491 for different models.

b) 1841 people were eligible for inclusion in analyses investigating going out shopping analyses. There were different amounts of missing data depending on variables included in the models, so n ranged between 1671 and 1713 for different models.

c) 374 people were eligible for inclusion in analyses investigating non-essential workplace attendance (sample limited to people who reported they could work entirely from home). Due to missing data, n included in analyses ranged between 349 and 354.

d) 410 people were eligible for inclusion in analyses investigating non-essential workplace attendance (sample limited to people who reported they could work entirely from home). Due to missing data, n included in analyses ranged between 379 and 389.

Table 2. Associations between highest risk social mixing and socio-demographic factors, adjusting for all other socio-demographic characteristics. Results reported are for block 1 of worry analyses. Bolding denotes significant findings (p<.002).

Attribute	Level	Highest risk social mixing				Wearing a face covering in shops				Wearing a face covering in hospitality venues			
		Wave 63.5 [a] aOR for engaging in highest risk social mixing (95% CI)	*p*	Wave 64 [b] aOR for engaging in highest risk social mixing (95% CI)		Wave 63.5 [c] aOR for wearing a face covering in shops (95% CI)	*p*	Wave 64 [d] aOR for wearing a face covering in shops (95% CI)	*p*	Wave 63.5 [e] aOR for wearing a face covering in hospitality venues (95% CI)	*p*	Wave 64 [f] aOR for wearing a face covering in hospitality venues (95% CI)	*p*
Region	East Midlands	Ref	-	Ref	-	Ref	-	Ref	-	Ref	-	Ref	-
	East of England	1.58 (0.99 to 2.54)	0.06	0.89 (0.57 to 1.39)	0.61	1.04 (0.54 to 2.02)	0.91	0.55 (0.26 to 1.18)	0.13	0.59 (0.29 to 1.24)	0.17	1.17 (0.60 to 2.25)	0.65
	London	1.36 (0.82 to 2.26)	0.23	0.94 (0.61 to 1.45)	0.77	1.12 (0.57 to 2.18)	0.75	0.48 (0.23 to 1.00)	0.05	0.52 (0.25 to 1.09)	0.08	0.91 (0.50 to 1.66)	0.76
	North East	1.52 (0.79 to 2.91)	0.21	1.31 (0.76 to 2.27)	0.33	0.99 (0.42 to 2.35)	0.99	0.46 (0.19 to 1.11)	0.08	0.47 (0.18 to 1.22)	0.12	2.04 (0.97 to 4.33)	0.06
	North West	1.25 (0.75 to 2.06)	0.39	1.17 (0.77 to 1.79)	0.45	0.86 (0.44 to 1.67)	0.65	**0.31 (0.15 to 0.62)**	**0.001**	0.44 (0.21 to 0.96)	0.04	0.67 (0.36 to 1.24)	0.20
	South East	1.59 (1.00 to 2.51)	0.05	1.17 (0.77 to 1.76)	0.46	0.93 (0.50 to 1.73)	0.81	0.49 (0.24 to 1.00)	0.05	0.51 (0.25 to 1.02)	0.06	1.28 (0.71 to 2.30)	0.42
	South West	1.34 (0.80 to 2.23)	0.27	1.03 (0.66 to 1.62)	0.89	1.02 (0.51 to 2.02)	0.96	0.44 (0.21 to 0.94)	0.03	0.65 (0.29 to 1.43)	0.28	1.27 (0.68 to 2.38)	0.45
	West Midlands	1.27 (0.77 to 2.11)	0.35	1.01 (0.65 to 1.59)	0.96	1.07 (0.54 to 2.11)	0.85	0.34 (0.16 to 0.71)	0.004	0.52 (0.24 to 1.10)	0.09	1.01 (0.52 to 1.97)	0.97
	Yorkshire and The Humber	1.34 (0.73 to 2.46)	0.35	1.21 (0.78 to 1.87)	0.40	1.21 (0.53 to 2.75)	0.65	0.33 (0.16 to 0.69)	0.003	0.26 (0.10 to 0.70)	0.008	1.22 (0.64 to 2.32)	0.54
	Overall	$\chi^2(8)=5.6$	0.69	$\chi^2(8)=5.0$	0.76	$\chi^2(8)=1.6$	0.99	$\chi^2(8)=15.2$	0.05	$\chi^2(8)=8.8$	0.36	$\chi^2(8)=12.3$	0.14
Sex	Male	Ref	-	Ref	-	Ref	-	Ref	-	Ref	-	Ref	-
	Female	1.40 (1.12 to 1.74)	0.003	1.35 (1.10 to 1.65)	0.003	**1.64 (1.22 to 2.21)**	**0.001**	1.37 (1.03 to 1.83)	0.03	1.34 (0.96 to 1.86)	0.08	1.46 (1.09 to 1.95)	0.01
Age (per decade)	Raw age	0.98 (0.90 to 1.06)	0.55	0.95 (0.88 to 1.02)	0.15	1.18 (1.05 to 1.32)	0.005	**1.22 (1.10 to 1.35)**	**<0.001**	1.03 (0.91 to 1.16)	0.66	1.06 (0.96 to 1.18)	0.24
Age: quadratic (age-mean)^2	-	1.0000 (0.9996 to 1.0004)	0.97	1.0002 (0.9999 to 1.0006)	0.20	1.0000 (0.9995 to 1.0006)	0.91	0.9998 (0.9993 to 1.0003)	0.40	0.9999 (0.9993 to 1.0005)	0.64	1.0006 (1.0001 to 1.0011)	0.02
Dependent child in household	None	Ref	-	Ref	-	Ref	-	Ref	-	Ref	-	Ref	-
	Child present	0.82 (0.63 to 1.06)	0.13	0.83 (0.65 to 1.05)	0.12	**0.56 (0.41 to 0.78)**	**0.001**	0.93 (0.68 to 1.28)	0.66	0.97 (0.67 to 1.41)	0.88	1.68 (1.19 to 2.37)	0.003
At high risk (self)	No	Ref	-	Ref	-	Ref	-	Ref	-	Ref	-	Ref	-
	Yes	0.90 (0.67 to 1.19)	0.45	0.77 (0.59 to 1.01)	0.05	1.14 (0.77 to 1.69)	0.50	0.80 (0.56 to 1.16)	0.24	1.32 (0.87 to 2.00)	0.19	1.34 (0.91 to 1.96)	0.13
	No	Ref	-	Ref	-	Ref	-	Ref	-	Ref	-	Ref	-

Household member has chronic illness	Yes	0.83 (0.61 to 1.13)	0.24	1.07 (0.81 to 1.41)	0.64	0.84 (0.55 to 1.27)	0.40	0.85 (0.58 to 1.25)	0.42	0.96 (0.60 to 1.55)	0.88	1.47 (0.97 to 2.22)	0.07
Employment status	Not working	Ref	-	Ref	-	Ref	-	Ref	-	Ref	-	Ref	-
	Working	0.76 (0.58 to 0.99)	0.04	0.77 (0.60 to 0.98)	0.04	0.98 (0.69 to 1.40)	0.92	1.01 (0.72 to 1.42)	0.96	0.63 (0.42 to 0.95)	0.03	0.96 (0.66 to 1.38)	0.81
Socio-economic grade‡	ABC1	Ref	-	Ref	-	Ref	-	Ref	-	Ref	-	Ref	-
	C2DE	0.89 (0.71 to 1.13)	0.34	0.79 (0.64 to 0.98)	0.03	0.76 (0.56 to 1.04)	0.09	0.74 (0.55 to 1.01)	0.06	0.84 (0.59 to 1.20)	0.34	0.88 (0.64 to 1.21)	0.43
Index of multiple deprivation	1st (least) to 4th quartile (most deprived)	1.02 (0.91 to 1.14)	0.71	1.03 (0.93 to 1.14)	0.54	1.01 (0.87 to 1.17)	0.93	1.07 (0.93 to 1.24)	0.34	0.99 (0.84 to 1.18)	0.95	1.04 (0.89 to 1.21)	0.61
Highest educational or professional qualification	Less than degree	Ref	-	Ref	-	Ref	-	Ref	-	Ref	-	Ref	-
	Degree or higher	1.05 (0.83 to 1.34)	0.68	1.05 (0.84 to 1.32)	0.66	1.16 (0.83 to 1.61)	0.38	1.21 (0.86 to 1.70)	0.27	1.03 (0.72 to 1.46)	0.88	1.40 (1.01 to 1.95)	0.04
Ethnicity	White British	Ref	-	Ref	-	Ref	-	Ref	-	Ref	-	Ref	-
	White other	1.16 (0.64 to 2.11)	0.63	0.99 (0.54 to 1.80)	0.96	1.17 (0.54 to 2.50)	0.69	0.84 (0.38 to 1.85)	0.67	0.52 (0.21 to 1.30)	0.16	1.57 (0.66 to 3.72)	0.31
	Black and minority ethnicity	0.69 (0.47 to 1.03)	0.07	0.81 (0.57 to 1.16)	0.25	1.39 (0.86 to 2.26)	0.18	1.65 (1.02 to 2.68)	0.04	1.49 (0.87 to 2.55)	0.14	1.40 (0.85 to 2.29)	0.19
	Overall	$\chi^2(2)=4.4$	0.11	$\chi^2(2)=1.5$	0.48	$\chi^2(2)=1.8$	0.41	$\chi^2(2)=5.4$	0.07	$\chi^2(2)=5.4$	0.07	$\chi^2(2)=2.1$	0.35
First language	Not English	Ref	-	Ref	-	Ref	-	Ref	-	Ref	-	Ref	-
	English	1.45 (0.86 to 2.47)	0.17	0.90 (0.55 to 1.49)	0.68	1.09 (0.58 to 2.02)	0.80	0.91 (0.46 to 1.78)	0.77	1.48 (0.72 to 3.03)	0.28	1.10 (0.56 to 2.16)	0.79
Ever had COVID-19	Think not	Ref	-	Ref	-	Ref	-	Ref	-	Ref	-	Ref	-
	Think yes	1.01 (0.78 to 1.31)	0.93	1.31 (1.03 to 1.66)	0.03	1.11 (0.80 to 1.54)	0.54	0.85 (0.62 to 1.18)	0.34	1.13 (0.78 to 1.64)	0.50	0.63 (0.45 to 0.87)	0.006
Vaccination status	Not vaccinated	Ref	-	Ref	-	Ref	-	Ref	-	Ref	-	Ref	-
	1 dose	0.72 (0.41 to 1.26)	0.25	0.61 (0.36 to 1.01)	0.06	2.62 (1.37 to 4.99)	0.003	**2.62 (1.48 to 4.66)**	**0.001**	**3.83 (1.86 to 7.88)**	**<0.001**	1.22 (0.63 to 2.39)	0.55
	2 doses or more	0.90 (0.64 to 1.26)	0.54	0.98 (0.71 to 1.35)	0.92	**2.31 (1.56 to 3.40)**	**<0.001**	**4.44 (3.05 to 6.48)**	**<0.001**	2.29 (1.35 to 3.87)	0.002	1.34 (0.84 to 2.15)	0.22
	Overall	$\chi^2(2)=1.4$	0.51	$\chi^2(2)=4.3$	0.11	**$\chi^2(2)=19.4$**	**<0.001**	**$\chi^2(2)=60.1$**	**<0.001**	**$\chi^2(2)=14.9$**	**0.001**	$\chi^2(2)=1.5$	0.48
Financial hardship	Range 3 (least) to 15 (most)	0.95 (0.92 to 0.98)	0.004	0.96 (0.92 to 0.99)	0.01	0.97 (0.93 to 1.02)	0.21	0.94 (0.9 to 0.99)	0.02	1.03 (0.97 to 1.08)	0.33	1.01 (0.96 to 1.06)	0.65

a) 1622 people were eligible for inclusion in highest risk social mixing analyses. There were different amounts of missing data depending on variables included in the models, so n ranged between 1439 and 1446 for different models.

b) 1841 people were eligible for inclusion in highest risk social mixing analyses. There were different amounts of missing data depending on variables included in the models, so n ranged between 1668 and 1689 for different models.

c) 1404 people were eligible for inclusion in wearing a face covering in shops analyses (sample limited to people who reported having been out shopping in the last week). There were different amounts of missing data depending on variables included in the models, so n ranged between 1247 and 1266 for different models.

d) 1600 people were eligible for inclusion in wearing a face covering in shops analyses (sample limited to people who reported having been out shopping in the last week). There were different amounts of missing data depending on variables included in the models, so n ranged between 1454 and 1475 for different models.

e) 789 people were eligible for inclusion in wearing a face covering in hospitality venues analyses (sample limited to people who reported having been out to hospitality venues in the last week). There were different amounts of missing data depending on variables included in the models, so n ranged between 700 and 713 for different models.

f) 894 people were eligible for inclusion in wearing a face covering in hospitality venues analyses (sample limited to people who reported having been out to hospitality venues in the last week). There were different amounts of missing data depending on variables included in the models, so n ranged between 817 and 829 for different models.
