# Supplementary material for: How has the emergence of the Omicron SARS-CoV-2 variant of concern influenced worry, perceived risk and behaviour in the UK? A series of cross-sectional surveys
Source: BMJ Open. 2022 Aug 29;12(8):e061203. doi: 10.1136/bmjopen-2022-061203 (PMC9437738; doi:10.1136/bmjopen-2022-061203)
Supplement: Author's manuscript [file bmjopen-2022-061203.draft_revisions.pdf]

BMJ Open is committed to open peer review. As part of this commitment we make the peer review history of every article we publish publicly available.

When an article is published we post the peer reviewers' comments and the authors' responses online. We also post the versions of the paper that were used during peer review. These are the versions that the peer review comments apply to.

The versions of the paper that follow are the versions that were submitted during the peer review process. They are not the versions of record or the final published versions. They should not be cited or distributed as the published version of this manuscript.

BMJ Open is an open access journal and the full, final, typeset and author-corrected version of record of the manuscript is available on our site with no access controls, subscription charges or pay-per-view fees (http://bmjopen.bmj.com).

If you have any questions on BMJ Open's open peer review process please email info.bmjopen@bmj.com

BMJ Open

How has the emergence of the Omicron SARS-CoV-2 variant of concern influenced worry, perceived risk, and behaviour in the UK? A series of cross-sectional surveys

Journal:	*BMJ Open*
Manuscript ID	bmjopen-2022-061203
Article Type:	Original research
Date Submitted by the Author:	19-Jan-2022

Complete List of Authors:	Smith, Louise; King's College London<br>Potts, Henry; University College London, Institute of Health Informatics<br>Amlot, Richard; UK Health Security Agency<br>Fear, Nicola; King's College London, King’s Centre for Military Health Research and Academic Department of Military Mental Health<br>Michie, Susan; University College London, Centre for Behaviour Change<br>Rubin, GJ; King's College London, Institute of Psychiatry, Psychology and Neuroscience

Keywords:	COVID-19, Public health < INFECTIOUS DISEASES, PUBLIC HEALTH

SCHOLARONE™
Manuscripts

I, the Submitting Author has the right to grant and does grant on behalf of all authors of the Work (as defined in the below author licence), an exclusive licence and/or a non-exclusive licence for contributions from authors who are: i) UK Crown employees; ii) where BMJ has agreed a CC-BY licence shall apply, and/or iii) in accordance with the terms applicable for US Federal Government officers or employees acting as part of their official duties; on a worldwide, perpetual, irrevocable, royalty-free basis to BMJ Publishing Group Ltd ("BMJ") its licensees and where the relevant Journal is co-owned by BMJ to the co-owners of the Journal, to publish the Work in this journal and any other BMJ products and to exploit all rights, as set out in our licence.

The Submitting Author accepts and understands that any supply made under these terms is made by BMJ to the Submitting Author unless you are acting as an employee on behalf of your employer or a postgraduate student of an affiliated institution which is paying any applicable article publishing charge ("APC") for Open Access articles. Where the Submitting Author wishes to make the Work available on an Open Access basis (and intends to pay the relevant APC), the terms of reuse of such Open Access shall be governed by a Creative Commons licence – details of these licences and which Creative Commons licence will apply to this Work are set out in our licence referred to above.

Other than as permitted in any relevant BMJ Author's Self Archiving Policies, I confirm this Work has not been accepted for publication elsewhere, is not being considered for publication elsewhere and does not duplicate material already published. I confirm all authors consent to publication of this Work and authorise the granting of this licence.

For peer review only

How has the emergence of the Omicron SARS-CoV-2 variant of concern influenced worry, perceived risk, and behaviour in the UK? A series of cross-sectional surveys

Louise E Smith (0000-0002-1277-2564),[1,2] PhD, Henry WW Potts (0000-0002-6200-8804),[3] PhD, Richard Amlôt (0000-0003-3481-6588),[2,4] PhD, Nicola T Fear (0000-0002-5792-2925),[1,5] DPhil (Oxon), Susan Michie (0000-0003-0063-6378),[6] DPhil, G James Rubin (0000-0002-4440-0570),[1,2] PhD

1 King's College London, Institute of Psychiatry, Psychology and Neuroscience

2 NIHR Health Protection Research Unit in Emergency Preparedness and Response

3 University College London, Institute of Health Informatics

4 UK Health Security Agency, Behavioural Science and Insights Unit

5 King's Centre for Military Health Research and Academic Department of Military Mental Health, King's College London

6 University College London, Centre for Behaviour Change

Corresponding author: Louise E Smith, Post-doctoral Researcher. Department of Psychological Medicine, King's College London, Weston Education Centre, Cutcombe Road, London, SE5 9RJ. Email: louise.e.smith@kcl.ac.uk. ORCID number: 0000-0002-1277-2564. Twitter handle: @louisesmith142

Henry WW Potts, Professor of Health Informatics. Institute of Health Informatics, 222 Euston Road, London, NW1 2DA.

Richard Amlôt, Head of Behavioural Science in the Behavioural Science and Insights Unit at the UK Health Security Agency. Porton Down, Salisbury, Wiltshire, SP4 0JG.

Nicola T Fear, Professor of Epidemiology. Department of Psychological Medicine, King's College London, Weston Education Centre, Cutcombe Road, London, SE5 9RJ.

Susan Michie, Professor of Health Psychology. Centre for Behaviour Change, University College London,1-19 Torrington Place, London, WC1E 7HB.

G James Rubin, Assistant Director, NIHR Health Protection Research Unit in Emergency Preparedness and Response, King's College London, Weston Education Centre, Cutcombe Road, London, SE5 9RJ.

Manuscript word count: 4445

For peer review only - http://bmjopen.bmj.com/site/about/guidelines.xhtml

Abstract

Objectives: To investigate: changes in beliefs and behaviours following news of the Omicron variant and changes to guidance; understanding of Omicron-related guidance; and factors associated with engaging with protective behaviours.

Design: Series of cross-sectional surveys (1 November to 16 December 2021, 5 waves of data collection).

Setting: Online.

Participants: People living in England, aged 16 years or over (n=1622 to 1902 per wave).

Primary and secondary outcome measures: Levels of worry and perceived risk, and engagement with key behaviours (out-of-home activities, risky social mixing, wearing a face covering, and testing uptake).

Results: Beliefs about worry and perceived risk of COVID-19 fluctuated over time, with worry, perceived risk to self and perceived risk to people increasing slightly around the time of the announcement about Omicron. Understanding of the new rules in England was low, with people over-estimating the stringency of the new rules. Rates of wearing a face covering increased over time, as did testing uptake. Meeting up with people from another household decreased around the time of the announcement of Omicron (29 November to 1 December), but then returned to previous levels. Associations with engagement with protective behaviours was investigated using regression analyses. There was no evidence for significant associations between out-of-home activity and worry or perceived risk (COVID-19 generally or Omicron-specific). Engaging in highest risk social mixing and always wearing a face covering in hospitality venues were associated with worry and perceived risk about COVID-19. Always wearing a face covering in shops was associated with having heard more about Omicron.

Conclusions: Almost two years into the COVID-19 outbreak, the emergence of a novel variant of concern only slightly influenced worry and perceived risk. The main protective behaviour (wearing a face covering) promoted by new guidance showed significant re-uptake, but other protective behaviours showed little or no change.

Abstract word count: 298

Key words: COVID-19, variant of concern, perceptions, behaviour, non-pharmaceutical interventions

For peer review only

For peer review only - http://bmjopen.bmj.com/site/about/guidelines.xhtml

Strengths and limitations of this study

- Rapid data collection, reporting on beliefs and behaviours immediately following news of the emergence of the Omicron variant of concern.
- Large sample size, and continued questions, allow for precise prevalence estimates and investigation of longer-term trends.
- Data are self-reported and may therefore represent an overestimation of engagement with protective behaviours.
- Data are cross-sectional, and we cannot imply the direction of associations.
- We are unsure of the representativeness of the beliefs and behaviours of people who sign up to take part in online surveys.

For peer review only

For peer review only - http://bmjopen.bmj.com/site/about/guidelines.xhtml

Introduction

The Omicron variant of SARS-CoV-2 was reported to the World Health Organization (WHO) on 24 November 2021 and was designated by the WHO as a variant of concern on 26 November 2021.(1) Since this date, it has attracted substantial media coverage.(2, 3) The emergence of the Omicron variant presented policymakers, and society more generally, with a dilemma. What action should be taken in the face of a rapidly spreading infection, the severity of which is unclear? The UK has witnessed intense debate around this question, with disagreements being played out across the national press, in the House of Commons, and in academic articles. In the early stages of the COVID-19 pandemic, the emergence of the original SARS-CoV-2 virus prompted similar controversy and led to modest increases in levels of worry among the UK public, with 40% engaging in recommended respiratory and hand hygiene behaviours, and 14% reducing the number of people that they met, a behaviour that had not then been officially recommended.(4)

England had removed legal COVID-19 restrictions about wearing a face covering and physical distancing on 19 July 2021.(5) This was followed by decreases in rates of protective behaviour.(6) In response to the Omicron variant, the UK Prime Minister, English Chief Medical Officer and Government Chief Scientific Advisor held a press conference on 27 November, the same day the first UK cases were reported,(7) in which new measures were announced.(8) These were implemented from 30 November 2021.(9) They included making face coverings compulsory in shops and on public transport, and requiring all international arrivals to take a polymerase chain reaction (PCR) test within two days of arriving in the UK and self-isolating until they receive a negative test result. (5, 6) Recommendations for all members of the public to use a lateral flow test regularly, and before meeting other people (epitomised by the slogan "lateral flow before you go" used in the Devolved Administrations(10)) were retained and reiterated.

As more evidence about the rapid spread of the Omicron variant appeared, on 8 December 2021, further measures were announced as part of the UK's "Plan B", with face coverings becoming compulsory in most public indoor venues (apart from hospitality), vaccine passports becoming mandatory in specific settings and people being asked to work from home where possible.(11) These changes came into effect on 13 December 2021. On 27 December, the Government announced no new restrictions for England before the end of the year.(12)

Throughout the pandemic, concern has been raised that public adherence to rules may wane over time.(13) Nonetheless, changes in rules have consistently caused changes in behaviour.(14) Research conducted during the COVID-19 and the 2009 H1N1 pandemics indicates engagement with protective behaviours was associated with having heard more about the pandemic,(4, 15) and increased worry about, and perceived risk of, infection.(16, 17) Public fears are known to be greater when risks are novel and uncertain.(18) While the risks of COVID-19 are now familiar to members of the public, the new variant represents a possible new source of public worry that may affect behaviour.

In this study, we investigated whether beliefs about COVID-19 and engagement with protective behaviours changed in the first three weeks of the emergence of the Omicron variant. We measured understanding of new guidance and satisfaction with the government response to Omicron. We also investigated whether engaging with protective behaviours was associated with amount heard about Omicron, worry (about COVID-19 generally and Omicron specifically), and perceived risk (of COVID-19 generally and Omicron specifically).

For peer review only - http://bmjopen.bmj.com/site/about/guidelines.xhtml

Methods

Design

Series of online cross-sectional surveys conducted by Savanta (a Market Research Society company partner). Surveys have been conducted since January 2020 on behalf of the English Department of Health and Social Care, and analysed by us as part of CORSAIR (COVID-19 Rapid Survey of Adherence to Interventions and Responses).(19) For these analyses, we used data collected in five waves: wave 61 (1-4 November 2021), wave 62 (15-17 November), wave 63 (29 November-2 December), an *ad hoc* wave added to the series to assess responses to Omicron (6-8 December 2021; wave 63.5), and wave 64 (13-16 December).

Data collection for wave 63 took place after the first news about Omicron and the announcement of new COVID-19 rules. It overlapped a period before and after the rules came into force (30 November 2021; see supplementary materials Figure 1). Because questions in each wave asked about behaviour over the previous week, behaviours asked about in wave 63 include a small amount of time before the news of Omicron first emerged and a longer period of time before the new rules in England were announced. Questions in wave 63.5 asking about behaviour all related to the period after news about Omicron appeared, but also covered a period before and after the new rules came into force. Further rules ("Plan B") were announced on 8 December 2021 and came into force on 13 December 2021 (when data collection for wave 64 started). Questions in wave 64 therefore ask about behaviour in the week before Plan B rules came in to place, with some participants answering about a small amount of time under Plan B rules and a larger amount of time before these rules. See Supplementary materials Figure 1 for a timeline.

Participants

Participants were recruited from a pool of people who had signed up to take part in online surveys (known as online research panels). Participants were eligible to take part if they were aged 16 years or over and lived in the UK. Non-probability sampling (quotas based on age and sex [combined], and region) was used to ensure the sample was broadly similar to the UK general population. After completing the survey, participants are unable to take part in the subsequent three waves of data collection. Participants were reimbursed in points which could be redeemed in cash, gift vouchers or charitable donations (up to 70p per survey).

We report figures for England only as the four nations of the UK made different changes for Omicron. We excluded participants in Wave 63.5 who completed the survey after the 8 December Government press conference began (n=58).

Study materials

Unless otherwise specified, participants answered all items.

Worry and perceived risk

Participants were asked "overall, how worried are you about coronavirus" on a five-point scale from "not at all worried" to "extremely worried". They were also asked "to what extent you think coronavirus poses a risk to…" them personally and people in the UK, on a five-point scale from "no risk at all" to "major risk". From wave 63.5, participants were also asked congruent questions about their worry about, and perceived risk of, Omicron. The items asked participants "Thinking about the Omicron variant, how worried are you about this specific variant of coronavirus?" and "to what extent you think this specific variant of coronavirus poses a risk…".

For peer review only - http://bmjopen.bmj.com/site/about/guidelines.xhtml

Worry and perceived risk (to oneself, others in the UK) were coded into separate binary variables (worry: very and extremely worried, versus somewhat, not very, and not at all worried; perceived risk: major and significant risk, versus moderate, minor, and no risk at all).

Behaviours

Participants were asked how many times in the last week they had done each of a list of twenty activities including shopping for groceries/pharmacy; shopping for other items; providing help or care for a vulnerable person; meeting up with friends or family that they did not live with; going to a restaurant, café or pub; using public transport or a taxi/minicab; and going out to work. Responses were capped at 30.

Participants who indicated that they had met up with friends or family from another household were asked a series of follow-up questions about the setting and number of people involved in their most recent meeting in the past seven days. We derived a measure categorising the risk of transmission involved in a participant's most recent instance of social mixing.(14) We were unable to calculate this measure for five participants.

Participants who indicated that they had visited a shop, hospitality venue, or used public transport or a minicab were asked whether they wore a face covering while doing so. Response options were "yes – on all occasions", "yes – on some occasions", and "no, not at all". We categorised people as wearing a face covering all the time, versus sometimes or not at all.

We asked participants when they last took a test for coronavirus. We categorised people as having tested if they indicated that they took their most recent test in the last week.

Amount heard about Omicron

From wave 63.5, participants were asked to indicate "how much, if anything, have you seen or heard about the new Omicron variant of coronavirus that was first detected in southern Africa?" on a four-point scale from "I have not seen or heard anything" to "I have seen or heard a lot".

Satisfaction with Government response

Participants in wave 63.5 onwards were asked to what extent they agreed or disagreed that "The Government was putting the right measures in place to protect the UK public from the Omicron variant of coronavirus", you "have enough information from the Government and other public authorities on the symptoms associated with the Omicron variant of coronavirus", and you "have enough information from the Government and other public authorities on how effective current vaccines are against the Omicron variant of coronavirus" on a five-point scale from strongly agree to strongly disagree.

Understanding of new rules

From wave 63.5, participants living in England were asked to indicate whether a series of nine statements about rules brought in to prevent the spread of Omicron were true, false, or they did not know. A tenth statement was added for wave 64. Statements included items about wearing a face covering in different locations (in shops, on public transport, in hospitality venues), self-isolation, and out-of-home behaviour.

Socio-demographic factors

We measured participants' age in years, sex, employment status, socio-economic grade, highest educational or professional qualification, ethnicity, their first language, COVID-19 vaccination status,

For peer review only - http://bmjopen.bmj.com/site/about/guidelines.xhtml

whether there was a dependent child in the household, whether they were at high risk for COVID-19,(20) whether a household member had a chronic illness, and whether they thought they had previously, or currently, had COVID-19 (recoded to a binary variable: “I’ve definitely had it, and had it confirmed by a test” and “I think I’ve probably had it”, vs “I don’t know whether I’ve had it or not”, “I think I’ve probably not had it”, and “I’ve definitely not had it”). Participants were also asked to report their full postcode, from which geographical region and indices of multiple deprivation were determined.(21)

To measure financial hardship, participants were asked to what extent in the past seven days they had been struggling to make ends meet, skipping meals they would usually have, and were finding their current living situation difficult (Cronbach’s α=.84).

Patient and public involvement

Lay members served on the advisory group for the project that developed our prototype survey material; this included three rounds of qualitative testing.(22) Due to the rapid nature of this research, the public was not involved in the further development of the materials during the COVID-19 pandemic.

Ethics

This work was conducted as a service evaluation of the Department of Health and Social Care’s public communications campaign. Following advice from King’s College London Research Ethics Committee, it was exempt from requiring ethical approval.

Power

A sample size of 1,600 per wave allows a 95% confidence interval of approximately plus or minus 2% for the prevalence estimate for a survey item with an overall prevalence of 50%.

Analysis

Unless otherwise specified, answers of “don’t know” were coded as missing.

We limited analyses investigating non-essential workplace attendance to participants who reported being in in full-, part-, or self-employment, and who indicated that they could work from home full-time. Questions about wearing a face covering were only asked to people who reported having completed that activity in the past seven days. Therefore, analyses were restricted to those who reported having been in shops, on public transport, and in hospitality venues in the last week.

We plotted worry and perceived risk, and behaviours by survey wave. For uptake of testing, we plotted two lines, including and excluding those whose most recent test was a polymerase chain reaction (PCR) test and who did not know their most recent test type. To investigate change over time, we used χ^2 analyses (categorical data), one-way ANOVAs (continuous data), and Kruskal-Wallis tests (skewed continuous data).

We present descriptive statistics of participants’ understanding of the new rules brought in in response to Omicron and satisfaction with the Government response.

To investigate associations with engagement with protective behaviours, we used data collected 6 to 8 December 2021 (wave 63.5) and 13 to 16 December (wave 64) separately as we hypothesised that people’s views and behaviour were likely to change due to the fast-moving nature of the spread of Omicron. We used negative binomial regression analyses (to account for skewed outcomes) to investigate associations with out-of-home activities (going out shopping, going to the workplace).

For peer review only - http://bmjopen.bmj.com/site/about/guidelines.xhtml

For these analyses, we summed the number of times participants reported going out shopping for groceries/pharmacy and other items, to give a total number of times gone shopping. We ran one model including only socio-demographic factors; a second that additionally included amount heard about Omicron, and perceived worry about COVID-19, perceived risk of COVID-19 to oneself, or perceived risk of COVID-19 to people in the UK; and a third that additionally included Omicron-specific worry or perceived risk. For these analyses, we report adjusted incidence rate ratios (aIRR).

For binary outcomes (risky social mixing: highest risk social mixing, vs other; always wearing a face covering in shops: wearing a face covering on all occasions, vs other; wearing a face covering in hospitality venues: wearing a face covering on all occasions, vs other), we used logistic regression analyses. Socio-demographic factors were entered as block one. Amount heard about Omicron and either worry about COVID-19, perceived risk of COVID-19 to oneself, or perceived risk of COVID-19 to people in the UK were entered as block two. Omicron-specific worry, perceived risk to self or perceived risk to people in the UK were entered as block three. For these analyses, we report adjusted odds ratios (aOR).

To account for the large number of analyses, we used a Bonferroni correction. For analyses investigating changes in beliefs and behaviour over time, we set significance at $p<0.003$ (n=22). For regression analyses, we set significance at $p<0.002$ (n=28).

Results

Participant characteristics

8941 responses were included in analyses (wave 61, n=1833; wave 62, n=1902; wave 63, n=1743; wave 63.5, n=1622; wave 64, n=1841). Respondents were slightly more likely to be women, and white compared to the general population (Table 1). Although there were significant differences in sex and ethnicity by survey wave, in practice, there were small differences between waves, with percentages differing at most by 1.2% (sex) and 3.3% (ethnicity).

Table 1. Participant characteristics

Attribute	Level	Wave 61, % (n) [total n=1833]	Wave 62, % (n) [total n=1902]	Wave 63, % (n) [total n=1743]	Wave 63.5, % (n) [total n=1622]	Wave 64, % (n) [total n=1841]	*p*
Sex	Male	46.8 (853)	47.2 (893)	46.7 (812)	45.8 (741)	47.0 (862)	<0.001
	Female	53.2 (968)	52.8 (999)	53.3 (925)	54.2 (878)	53.0 (973)	
Age	Range 16 to >90 years	M=48.7, SD=19.1	M=47.8, SD=18.8	M=49.1, SD=18.2	M=47.7, SD=18.4	M=47.7, SD=18.8	0.07
Employment status	Not working	46.8 (844)	44.8 (840)	45.5 (786)	44 (707)	44.7 (813)	0.25
	Working	53.2 (959)	55.2 (1033)	54.5 (943)	56 (899)	55.3 (1005)	
Index of multiple deprivation	1st (least) to 4th quartile (most deprived)	M=2.7, SD=1.0	M=2.7, SD=1.0	M=2.7, SD=1.0	M=2.8, SD=1.0	M=2.7, SD=1.0	0.62
Highest educational or professional qualification	Less than degree	65.4 (1198)	67.1 (1277)	66.8 (1165)	65.9 (1069)	67.5 (1243)	0.63
	Degree or higher	34.6 (635)	32.9 (625)	33.2 (578)	34.1 (553)	32.5 (598)	
Ethnicity	White British	82.2 (1498)	82.7 (1563)	84.2 (1460)	82.4 (1329)	82.0 (1505)	<0.001
	White other	6.1 (111)	5.1 (96)	5.5 (96)	5.1 (82)	4.5 (83)	
	Black and minority ethnicity	11.7 (214)	12.2 (231)	10.2 (177)	12.5 (202)	13.5 (247)	

For peer review only - http://bmjopen.bmj.com/site/about/guidelines.xhtml

Beliefs and behaviours over time

Beliefs about worry and perceived risk of COVID-19 fluctuated over time, with worry, perceived risk to self and perceived risk to people increasing slightly around the time of the announcement about the Omicron variant, then returning to pre-Omicron levels (worry (F(4,8921)=10.08, p<.001); perceived risk to self (F(4,8857)=7.10, p<.001); perceived risk to people in UK (F(4,8854)=5.12, p<.001); Figure 1).

Meeting up with people from another household changed between 1 November and 16 December 2021 (H(4)=17.4, n=8941, p=0.002; Figure 2). This change was driven by a decrease in reported rates in data collected on 29 November to 1 December 2021 (around the time of the announcement of Omicron) compared to the previous survey wave. Providing help or care for a vulnerable person also changed between 1 November and 16 December 2021 (H(4)=17.0, n=8941, p=0.002), with this change being driven by an increase in reported rates in data collected on 15 to 17 November 2021 compared to the previous survey wave. There were no other significant changes in out-of-home activity over time (been to the shops, for groceries/pharmacy (H(4)=7.5, n=8941, p=0.11); been to the shops, for things other than groceries/pharmacy (H(4)=8.4, n=8941, p=0.08); been to a restaurant, café or pub (H(4)=7.0, n=8941, p=0.14); used public transport or been in a taxi/minicab (H(4)=1.1, n=8941, p=0.90); left home to go to out to work (number of days) (H(4)=4.3, n=1904, p=0.36).

There were no differences in social mixing over time, stratified by risk of transmission (H(4)=8.9, p=.06; Figure 3).

Rates of always wearing a face covering increased over time in all settings (in shops for groceries / pharmacy (χ^2(4)=286.0, n=7815, p<.001); in a restaurant, café or pub (χ^2(4)=90.9, n=4497, p<.001); on public transport or in a taxi/minicab (χ^2(4)=50.8, n=3310, p<.001); Figure 4).

Rates of testing increased over time (whole sample (χ^2=33.2 (4), n=8780, p<.001; excluding people whose most recent test was a PCR test or who did not know what their most recent test type was (χ^2=32.4 (4), n=7912, p<.001); Figure 5).

Omicron worry, perceived risk, and amount heard

39.0% (95% CI 36.6% to 41.4%, n=624, wave 63.5) to 42.7% (95% CI 40.4% to 45.0%, n=777) of people reported being very or extremely worried about the Omicron variant (F(1,3417)=4.74, p=.03; Table 2; Figure 1). 44.9% (95% CI 42.5% to 47.4%, n=703, wave 63.5) to 46.4% (95% CI 44.1% to 48.7%, n=840; F(1,3371)=0.75, p=.39) perceived a major or significant risk of Omicron to themselves, with 56.7% (95% CI 54.2% to 59.1%, n=892, wave 63.5) to 61.4% (95% CI 59.1% to 63.6%, n=1116, wave 64; F(1,3391)=7.67, p=.006) of respondents perceiving a major or significant risk of Omicron to people in the UK.

For peer review only - http://bmjopen.bmj.com/site/about/guidelines.xhtml

Table 2. Perceived worry about, and risk of, Omicron variant.

	Thinking about the Omicron variant, how worried are you about this specific variant of coronavirus?					Still thinking about the Omicron variant, to what extent do you think this specific variant of coronavirus poses a risk to you personally				Still thinking about the Omicron variant, to what extent do you think this specific variant of coronavirus poses a risk to people in the UK					How much, if anything, have you seen or heard about the new Omicron variant of coronavirus that was first detected in southern Africa?			
	Wave 63.5 [total n=1622]		Wave 64 [total n=1841]			Wave 63.5 [total n=1622]		Wave 64 [total n=1841]		Wave 63.5 [total n=1622]		Wave 64 [total n=1841]			Wave 63.5 [total n=1622]		Wave 64 [total n=1841]	
	% (95% CI)	n	% (95% CI)	n		% (95% CI)	n	% (95% CI)	n	% (95% CI)	n	% (95% CI)	n		% (95% CI)	n	% (95% CI)	n
Extremely worried	14.1 (12.5 to 15.9)	229	17.2 (15.4 to 18.9)	316	Major risk	20.6 (18.7 to 22.6)	334	23.2 (21.3 to 25.1)	427	25.5 (23.4 to 27.7)	414	29.0 (26.9 to 31.0)	533	I have seen or heard a lot	31.6 (29.3 to 33.9)	512	37.1 (34.9 to 39.3)	683
Very worried	24.4 (22.3 to 26.5)	395	25.0 (23.1 to 27.0)	461	Significant risk	22.7 (20.7 to 24.8)	369	22.4 (20.5 to 24.3)	413	29.5 (27.3 to 31.8)	478	31.7 (29.5 to 33.8)	583	I have seen or heard a fair amount	47.7 (45.3 to 50.2)	774	44.2 (41.9 to 46.5)	814
Somewhat worried	36.9 (34.6 to 39.3)	599	34.1 (31.9 to 36.3)	628	Moderate risk	27.9 (25.7 to 30.1)	452	26.6 (24.5 to 28.6)	489	27.2 (25.0 to 29.4)	441	23.8 (21.8 to 25.7)	438	I have seen or heard a little	18.7 (16.8 to 20.7)	303	16.3 (14.6 to 18.0)	300
Not very worried	16.5 (14.7 to 18.4)	267	14.4 (12.8 to 16.0)	265	Minor risk	20.3 (18.4 to 22.3)	329	21.0 (19.1 to 22.8)	386	12.2 (10.7 to 13.9)	198	11.2 (9.8 to 12.7)	207	I have not seen or heard anything	1.7 (1.1 to 2.4)	27	2.0 (1.3 to 2.6)	36
Not at all worried	6.7 (5.6 to 8.1)	109	8.1 (6.9 to 9.4)	150	No risk at all	4.9 (3.9 to 6.1)	80	5.1 (4.1 to 6.1)	94	2.7 (1.9 to 3.6)	43	3.2 (2.4 to 3.9)	58					
Don’t know	1.4 (0.9 to 2.1)	23	1.1 (0.7 to 1.6)	21	Don’t know	3.6 (2.7 to 4.6)	58	1.7 (1.1 to 2.3)	32	3.0 (2.2 to 3.9)	48	1.2 (0.7 to 1.7)	22	Don't know	0.4 (0.1 to 0.8)	6	0.4 (0.1 to 0.7)	8

For peer review only - http://bmjopen.bmj.com/site/about/guidelines.xhtml

Understanding of new rules

Understanding of the new rules introduced in response to Omicron was varied (Table 3). Respondents scored well on rules requiring behaviour (around 80%+ correct, 90%+ correct on some rules). However, other items were answered incorrectly by most people, in the direction of believing that the rules were stricter than was the case. For some items (wearing a face covering in hospitality venues and all crowded and enclosed spaces), the percentage over-estimating the rules increased from Wave 63.5 to Wave 64. From 13 December 2021, people were asked to work from home if possible. This was the only rule that changed between survey waves, with high recognition in the latter wave.

For peer review only

For peer review only - http://bmjopen.bmj.com/site/about/guidelines.xhtml

Table 3. Endorsement of rules introduced in response to Omicron. Bold answers are correct.

The Government has issued new rules on how people should act to help prevent the spread of the Omicron variant of coronavirus. Please tell us, for the following options, if you think they are true or false?	Wave 63.5 [total n=1622]						Wave 64 [total n=1841]					
	True		False		Don't know		True		False		Don't know	
	% (95% CI)	n	% (95% CI)	n	% (95% CI)	n	% (95% CI)	n	% (95% CI)	n	% (95% CI)	n
You must wear a face covering in shops (unless you are exempt)	**91.9 (90.5 to 93.2)**	**1490**	5.6 (4.5 to 6.7)	91	2.5 (1.8 to 3.3)	41	**90.3 (88.9 to 91.6)**	**1622**	5.4 (4.4 to 6.5)	100	4.3 (3.4 to 5.2)	79
You must wear a face covering on public transport (unless you are exempt)	**91.1 (89.7 to 92.5)**	**1477**	6.2 (5.0 to 7.3)	100	2.8 (2.0 to 3.6)	45	**91.7 (90.5 to 93.0)**	**1689**	4.8 (3.8 to 5.8)	88	3.5 (2.6 to 4.3)	64
You must wear a face covering while moving around in restaurants, cafés and pubs (unless you are exempt)	64.5 (62.2 to 66.8)	1046	**28.2 (26.0 to 30.4)**	**457**	7.3 (6.1 to 8.6)	119	71.2 (69.1 to 73.3)	1311	**19.5 (17.7 to 21.3)**	**359**	9.3 (8.0 to 10.6)	171
You must wear a face covering in all crowded and enclosed spaces where you come into contact with people you don't usually meet (unless you are exempt)	77.9 (75.8 to 79.9)	1263	**15.2 (13.4 to 16.9)**	**246**	7.0 (5.7 to 8.2)	113	83.5 (81.8 to 85.2)	1538	**10.3 (8.9 to 11.7)**	**190**	6.1 (5.0 to 7.2)	113
All contacts of suspected Omicron cases must self-isolate, regardless of their vaccination status	**80.1 (78.1 to 82)**	**1299**	9.1 (7.7 to 10.5)	148	10.8 (9.3 to 12.3)	175	**76.9 (75.0 to 78.8)**	**1416**	12.7 (11.1 to 14.2)	233	10.4 (9.0 to 11.8)	192
You should stay at home as much as you can	61.7 (59.3 to 64.1)	1001	**27.2 (25.0 to 29.4)**	**441**	11.1 (9.6 to 12.6)	180	69.5 (67.4 to 71.6)	1280	**20.4 (18.6 to 22.3)**	**376**	10.0 (8.7 to 11.4)	185
You should work from home if possible	69.5 (67.3 to 71.8)	1128	**20.2 (18.3 to 22.2)**	**328**	10.2 (8.8 to 11.7)	166	**90.4 (89.0 to 91.7)**	**1664**	5.6 (4.6 to 6.7)	104	4.0 (3.1 to 4.9)	73
You cannot meet other people indoors, unless you live with them, or they are part of your support bubble	38.1 (35.7 to 40.5)	618	**49.1 (46.6 to 51.5)**	**796**	12.8 (11.2 to 14.5)	208	36.1 (33.9 to 38.3)	665	**49.8 (47.5 to 52.1)**	**917**	14.1 (12.5 to 15.7)	259
International arrivals must take a PCR test by the end of the second day after arrival and self-isolate until they receive a negative result	**84 (82.2 to 85.8)**	**1363**	7.6 (6.3 to 8.9)	123	8.4 (7.0 to 9.7)	136	**81.4 (79.6 to 83.2)**	**1499**	6.5 (5.3 to 7.6)	119	12.1 (10.6 to 13.6)	223
You must wear a face covering at the cinema or theatre	-	-	-	-	-	-	**85.2 (83.5 to 86.8)**	**1568**	7.4 (6.2 to 8.6)	137	7.4 (6.2 to 8.6)	136

For peer review only - http://bmjopen.bmj.com/site/about/guidelines.xhtml

Less than half of respondents agreed that the Government were putting the right measures in place to protect the UK public from Omicron, with around half agreeing that they had enough information about the symptoms of the Omicron variant and the effectiveness of vaccines against Omicron variant (Table 4). Most people agreed that they had enough information about what to do to prevent the spread of Omicron.

For peer review only

For peer review only - http://bmjopen.bmj.com/site/about/guidelines.xhtml

Table 4. Satisfaction with Government response to Omicron.

	The Government is putting the right measures in place to protect the UK public from the Omicron variant of coronavirus, % (n)		I have enough information from the Government and other public authorities on the symptoms of the Omicron variant of coronavirus, % (n)		I have enough information from the Government and other public authorities on how effective current vaccines are against the Omicron variant of coronavirus, % (n)		I have enough information from the Government and public authorities about what I can do to help prevent the spread of the Omicron variant of coronavirus, % (n)	
	Wave 63.5 [total n=1622]	Wave 64 [total n=1841]	Wave 63.5 [total n=1622]	Wave 64 [total n=1841]	Wave 63.5 [total n=1622]	Wave 64 [total n=1841]	Wave 63.5 [total n=1622]	Wave 64 [total n=1841]
Strongly agree	12.5 (203)	12.2 (224)	12.5 (203)	13.1 (242)	12.0 (195)	15.6 (287)	18.6 (301)	18.3 (336)
Agree	34.3 (557)	31.6 (581)	33.7 (546)	36.9 (680)	36.1 (585)	39.5 (728)	49.3 (799)	49.6 (913)
Neither agree nor disagree	23.0 (373)	22.8 (420)	23.9 (388)	21.6 (398)	22.7 (369)	21.0 (387)	17.8 (288)	18.2 (335)
Disagree	17.3 (281)	19.3 (356)	21.9 (355)	19.6 (360)	19.1 (310)	16.5 (303)	9.2 (149)	9.4 (173)
Disagree strongly	10.9 (176)	12.1 (222)	6.5 (105)	7.7 (142)	8.2 (133)	6.2 (115)	4.2 (68)	3.7 (69)
Don't know	2.0 (32)	2.1 (38)	1.5 (25)	1.0 (19)	1.8 (30)	1.1 (21)	1.0 (17)	0.8 (15)
Total strongly agree + agree, % (95% CI)	47.8 (45.3 to 50.3)	44.6 (42.4 to 46.9)	46.9 (44.5 to 49.4)	50.6 (48.3 to 52.9)	49.0 (46.5 to 51.5)	55.8 (53.5 to 58.1)	68.5 (66.3 to 70.8)	68.4 (66.3 to 70.5)
Total neither agree nor disagree + disagree + disagree strongly, % (95% CI)	52.2 (49.7 to 54.7)	55.4 (53.1 to 57.6)	53.1 (50.6 to 55.5)	49.4 (47.1 to 51.7)	51.0 (48.5 to 53.5)	44.2 (41.9 to 46.5)	31.5 (29.2 to 33.7)	31.6 (29.5 to 33.7)

For peer review only

For peer review only - http://bmjopen.bmj.com/site/about/guidelines.xhtml

Factors associated with engaging with protective behaviours

There were no significant associations between out-of-home activity and amount heard about Omicron, perceived worry (COVID-19 generally or Omicron specifically) or perceived risk (to oneself or people in UK, COVID-19 generally or Omicron specifically; Table 5). There were no associations with socio-demographic characteristics, with the exception of greater financial hardship being associated with going out shopping for items other than groceries/pharmacy (see supplementary materials Table 1).

For peer review only

Table 5. Associations between out-of-home activities and amount heard about Omicron, perceived worry, risk to self and risk to people in the UK. Bolding denotes significant findings (p<.002).

Attribute	Level	Going out shopping (for groceries/pharmacy and other items) Wave 63.5 [a] IRR for going out shopping (95% CI)	*p*	Wave 64 [b] IRR for going out shopping (95% CI)	*p*	Attending the workplace Wave 63.5 [c] IRR for attending the workplace (95% CI)	*p*	Wave 64 [d] aIRR for attending the workplace (95% CI)	*p*
Amount heard about Omicron variant †	I have not seen or heard anything (1) to I have seen or heard a lot (4)	1.05 (0.97 to 1.14)	0.27	1.08 (1.00 to 1.16)	0.05	1.03 (0.86 to 1.24)	0.72	1.02 (0.86 to 1.20)	0.85
Worry about COVID-19 †	Not at all worried (1) to extremely worried (5)	0.92 (0.87 to 0.97)	0.004	0.96 (0.91 to 1.01)	0.08	1.07 (0.93 to 1.22)	0.33	1.01 (0.89 to 1.14)	0.89
Worry about Omicron variant ‡	Not at all worried (1) to extremely worried (5)	0.93 (0.86 to 1.02)	0.12	0.93 (0.86 to 1.01)	0.10	0.93 (0.78 to 1.12)	0.44	0.97 (0.80 to 1.18)	0.76
Amount heard about Omicron variant †	I have not seen or heard anything (1) to I have seen or heard a lot (4)	1.04 (0.96 to 1.13)	0.30	1.08 (1.00 to 1.16)	0.05	1.06 (0.88 to 1.27)	0.56	1.02 (0.86 to 1.20)	0.85
Perceived risk of COVID-19 to self †	No risk at all (1) to major risk (5)	0.95 (0.90 to 1.00)	0.05	0.99 (0.94 to 1.04)	0.65	0.97 (0.87 to 1.09)	0.61	1.03 (0.92 to 1.15)	0.62
Perceived risk of Omicron variant to self ‡	No risk at all (1) to major risk (5)	0.99 (0.91 to 1.08)	0.90	1.02 (0.94 to 1.11)	0.57	1.09 (0.92 to 1.29)	0.31	1.04 (0.85 to 1.28)	0.71
Amount heard about Omicron variant †	I have not seen or heard anything (1) to I have seen or heard a lot (4)	1.04 (0.96 to 1.13)	0.34	1.07 (0.99 to 1.15)	0.08	1.05 (0.88 to 1.26)	0.59	1.01 (0.85 to 1.20)	0.90
Perceived risk of COVID-19 to people in UK †	No risk at all (1) to major risk (5)	0.96 (0.91 to 1.02)	0.21	0.98 (0.93 to 1.03)	0.46	1.01 (0.88 to 1.15)	0.91	1.00 (0.89 to 1.12)	0.95
Perceived risk of Omicron variant to people in UK ‡	No risk at all (1) to major risk (5)	0.92 (0.85 to 1.00)	0.05	1.10 (1.02 to 1.19)	0.02	1.13 (0.96 to 1.33)	0.15	1.01 (0.84 to 1.21)	0.93

† Adjusting for all other socio-demographic characteristics; amount heard about Omicron, and worry about COVID-19 / perceived risk of COVID-19 to self / perceived risk of COVID-19 to people in the UK.

‡ Adjusting for all other socio-demographic characteristics; amount heard about Omicron, and worry about COVID-19 / perceived risk of COVID-19 to self / perceived risk of COVID-19 to people in the UK; and Omicron-specific worry / perceived risk to self / perceived to people in the UK.

a) 1622 people were eligible for inclusion in analyses investigating going out shopping analyses. There were different amounts of missing data depending on variables included in the models, so n ranged between 1440 and 1491 for different models.

b) 1841 people were eligible for inclusion in analyses investigating going out shopping analyses. There were different amounts of missing data depending on variables included in the models, so n ranged between 1671 and 1713 for different models.

c) 372 people were eligible for inclusion in analyses investigating non-essential workplace attendance (sample limited to people who reported they could work entirely from home). Due to missing data, n included in analyses ranged between 349 and 354.

d) 410 people were eligible for inclusion in analyses investigating non-essential workplace attendance (sample limited to people who reported they could work entirely from home). Due to missing data, n included in analyses ranged between 379 and 389.

For peer review only - http://bmjopen.bmj.com/site/about/guidelines.xhtml

Engaging in highest risk social mixing and always wearing a face covering in hospitality venues were associated with worry about, and perceived risk of, COVID-19 (Table 6). Always wearing a face covering in shops was independently associated with having heard more about Omicron. Associations between behaviour and Omicron-specific worry and perceived risk often did not reach our statistical significance level required after a Bonferroni correction but showed some relationship with behaviour. Always wearing a face covering was associated with having been vaccinated (see supplementary materials Table 2).

For peer review only

For peer review only - http://bmjopen.bmj.com/site/about/guidelines.xhtml

Table 6. Associations between highest risk social mixing and wearing a face covering and amount heard about Omicron, perceived worry, risk to self and risk to people in the UK. Bolding denotes significant findings (p<.002).

		Highest risk social mixing				Always wearing a face covering in shops				Always wearing a face covering in hospitality venues			
Attribute	Level	Wave 63.5 [a] aOR for engaging in highest risk social mixing (95% CI)	*p*	Wave 64 [b] aOR for engaging in highest risk social mixing (95% CI)	*p*	Wave 63.5 [c] aOR for wearing a face covering in shops (95% CI)	*p*	Wave 64 [d] aOR for wearing a face covering in shops (95% CI)	*p*	Wave 63.5 [e] aOR for wearing a face covering in hospitality venues (95% CI)	*p*	Wave 64 [f] aOR for wearing a face covering in hospitality venues (95% CI)	*p*
Amount heard about Omicron variant †	I have not seen or heard anything (1) to I have seen or heard a lot (4)	1.03 (0.89 to 1.20)	0.69	1.09 (0.94 to 1.25)	0.25	**1.47 (1.20 to 1.79)**	**<0.001**	1.32 (1.09 to 1.59)	0.004	1.11 (0.89 to 1.39)	0.35	1.26 (1.03 to 1.54)	0.03
Worry about COVID-19 †	Not at all worried (1) to extremely worried (5)	**0.79 (0.71 to 0.88)**	**<0.001**	**0.73 (0.66 to 0.80)**	**<0.001**	**1.43 (1.23 to 1.65)**	**<0.001**	1.43 (1.26 to 1.64)	**<0.001**	**1.55 (1.31 to 1.84)**	**<0.001**	**1.34 (1.17 to 1.55)**	**<0.001**
Worry about Omicron variant ‡	Not at all worried (1) to extremely worried (5)	**0.76 (0.65 to 0.89)**	**0.001**	0.93 (0.79 to 1.09)	0.35	1.21 (0.98 to 1.50)	0.07	1.33 (1.07 to 1.65)	0.009	1.17 (0.94 to 1.46)	0.16	1.26 (1.02 to 1.55)	0.04
Amount heard about Omicron variant †	I have not seen or heard anything (1) to I have seen or heard a lot (4)	0.99 (0.85 to 1.15)	0.91	1.04 (0.90 to 1.19)	0.61	**1.50 (1.23 to 1.84)**	**<0.001**	1.33 (1.10 to 1.61)	0.003	1.17 (0.93 to 1.46)	0.18	1.33 (1.08 to 1.63)	0.006
Perceived risk of COVID-19 to self †	No risk at all (1) to major risk (5)	**0.85 (0.76 to 0.94)**	**0.001**	**0.78 (0.72 to 0.86)**	**<0.001**	**1.39 (1.21 to 1.60)**	**<0.001**	1.25 (1.11 to 1.42)	**<0.001**	**1.35 (1.16 to 1.57)**	**<0.001**	**1.24 (1.09 to 1.41)**	**0.001**
Perceived risk of Omicron variant to self ‡	No risk at all (1) to major risk (5)	0.85 (0.72 to 0.99)	0.04	0.90 (0.78 to 1.05)	0.18	1.20 (0.97 to 1.48)	0.10	1.22 (0.99 to 1.49)	0.06	1.25 (0.99 to 1.59)	0.07	1.02 (0.83 to 1.26)	0.84
Amount heard about Omicron variant †	I have not seen or heard anything (1) to I have seen or heard a lot (4)	1.01 (0.86 to 1.17)	0.95	1.04 (0.91 to 1.20)	0.57	**1.53 (1.25 to 1.87)**	**<0.001**	1.35 (1.12 to 1.63)	0.002	1.16 (0.93 to 1.45)	0.20	1.33 (1.09 to 1.63)	0.006

For peer review only

For peer review only - http://bmjopen.bmj.com/site/about/guidelines.xhtml

Perceived risk of COVID-19 to people in UK †	No risk at all (1) to major risk (5)	**0.82 (0.73 to 0.92)**	**<0.001**	**0.83 (0.75 to 0.92)**	**<0.001**	**1.28 (1.10 to 1.48)**	**0.001**	**1.41 (1.23 to 1.61)**	**<0.001**	**1.41 (1.19 to 1.66)**	**<0.001**	**1.28 (1.11 to 1.47)**	**0.001**
Perceived risk of Omicron variant to people in UK ‡	No risk at all (1) to major risk (5)	0.93 (0.8 to 1.09)	0.38	0.91 (0.78 to 1.05)	0.20	1.34 (1.09 to 1.64)	0.005	1.37 (1.12 to 1.68)	0.003	1.42 (1.13 to 1.77)	0.002	1.20 (0.98 to 1.46)	0.08

† Adjusting for all other socio-demographic characteristics; amount heard about Omicron, and worry about COVID-19 / perceived risk of COVID-19 to self / perceived risk of COVID-19 to people in the UK.

‡ Adjusting for all other socio-demographic characteristics; amount heard about Omicron, and worry about COVID-19 / perceived risk of COVID-19 to self / perceived risk of COVID-19 to people in the UK; and Omicron-specific worry / perceived risk to self / perceived to people in the UK.

a) 1622 people were eligible for inclusion in highest risk social mixing analyses. There were different amounts of missing data depending on variables included in the models, so n ranged between 1439 and 1446 for different models.

b) 1841 people were eligible for inclusion in highest risk social mixing analyses. There were different amounts of missing data depending on variables included in the models, so n ranged between 1668 and 1689 for different models.

c) 1404 people were eligible for inclusion in wearing a face covering in shops analyses (sample limited to people who reported having been out shopping in the last week). Due to missing data, n included in analyses ranged between 1247 and 1266.

d) 1600 people were eligible for inclusion in wearing a face covering in shops analyses (sample limited to people who reported having been out shopping in the last week). There were different amounts of missing data depending on variables included in the models, so n ranged between 1454 and 1475 for different models.

e) 789 people were eligible for inclusion in wearing a face covering in hospitality venues analyses (sample limited to people who reported having been out to hospitality venues in the last week). Due to missing data, n included in analyses ranged between 700 and 713.

f) 894 people were eligible for inclusion in wearing a face covering in hospitality venues analyses (sample limited to people who reported having been out to hospitality venues in the last week). There were different amounts of missing data depending on variables included in the models, so n ranged between 817 and 829 for different models.

For peer review only

Discussion

Our data suggest that initial reporting around the emergence of Omicron had relatively little impact on public perceptions. There were small changes in worry about, and perceived risk of, COVID-19 days after the emergence of Omicron was reported. While over one third of participants reported being very or extremely worried about Omicron, and over half of respondents perceived a major or significant risk of Omicron to people in the UK, these figures were very close to the rates observed for concerns about 'coronavirus' in general.

Engagement with certain protective behaviours (wearing a face covering, testing) increased between 1 November and 16 December 2021. Approximately 80% of the sample reported "always" wearing a face covering while in shops. This rate is similar to the percentage who reported "frequently" or "very frequently" wearing a face covering outside the home during the second lockdown in England (November 2020).(24) Rates of wearing a face covering increased even in hospitality settings, where rules were not changed, possibly reflecting the misunderstanding of the extent of official guidance that we observed. A survey by the English Office for National Statistics has also shown an increase in wearing a face covering in data collected 1 to 12 December 2021.(25) Increases in uptake of testing may reflect a higher prevalence of symptoms in the population during this period.(26) While there have been media reports of behaviour change in response to Omicron (for example, restaurant industry figures reporting a fall in eating out early on),(27) our results show that there were few changes in out-of-home activity up to 16 December 2021. This is in line with other polling carried out on 14 to 15 December 2021.(28) Despite Omicron being a key story in the media, it appears that early behavioural responses to it were largely restricted to changes that were required by legislation, rather than more spontaneous changes among the public.

Despite over one-third of people thinking that indoor mixing with other households was not allowed, there were no changes in patterns of social mixing. Our question on knowledge of the rules may be insensitive to degrees of certainty or may be demonstrating a social desirability effect. Social mixing may normally increase in the run-up to Christmas, so we cannot tell whether a flat statistic actually represents a reduction compared to the usual pattern for the time of year. Nonetheless, in contrast to the early stages of the COVID-19 pandemic, we have not yet observed a substantial "spill-over" effect involving non-recommended behaviours following the emergence of the Omicron variant.

Previous research has suggested that a constant stream of changes to guidance over the course of the pandemic has left many people confused and disengaged.(29, 30) Understanding of the new rules in response to Omicron was mixed. In general, people greatly over-estimated the stringency of the rules. This has the potential to be positive in terms of reducing transmission, but could also have a negative impact in terms of wellbeing,(31) economic activity,(27) and social tension.(32) Additional rules were introduced on 13 December 2021 (England's "plan B", working from home where possible, face coverings becoming compulsory in most public indoor venues apart from hospitality, introduction of vaccine passports in some settings).(11) Recognition of the rule regarding working from home increased in data collected 13 to 16 December, but there was no evidence for a corresponding change in behaviour. This is likely because we measured behaviour in the previous week, before the rule was introduced. Furthermore, there was no legal underpinning to this rule in England, unlike during the third UK lockdown.(33)

We investigated associations between engaging in protective behaviours that had and had not been legislated, and worry and perceived risk. There were no associations for out-of-home activity (shopping and non-essential workplace attendance). Other behaviours for which there was no change in legislation (engaging in highest risk social mixing, wearing a face covering in hospitality

For peer review only - http://bmjopen.bmj.com/site/about/guidelines.xhtml

venues) were associated independently with worry about, and perceived risk of, COVID-19 in general. Analyses investigating associations with a behaviour that was legislated (wearing a face covering in shops) found that use was initially associated with greater amount heard about Omicron (wave 63.5). Data are cross-sectional and we cannot tell the direction of causation. It may be the case that people wear face coverings are more likely to pay attention to news about COVID-19.

To the best of our knowledge, this is the first paper investigating the influence of the Omicron variant on public worry, perceived risk and behaviour. This rapid response was facilitated by having regular data collection measuring public behaviour and attitudes. Limitations of the study include the use of self-reported data. We have previously noted that self-reported face covering wearing is likely to over-estimate observed rates, although self-reports of "always" wearing a face covering in a particular location appear more robust.(6) Participants in our study were slightly more likely to be female and white than the general population. Whether the behaviour and attitudes of people who sign up to take part in surveys is representative of the behaviour and attitudes of the general population is unknown. Participants were asked to report on their behaviour in the last week. For wave 63 and 64 data, this overlapped the period before and after rules (in response to the Omicron variant, and England's "Plan B" respectively) came into force. We did not investigate factors associated with all potential out-of-home activities, nor uptake of testing, as this would have been too many outcomes. We focused our analyses on activities where the chance of coming into close contact with people from other households was greatest, and where legislation had recently changed. We investigated wearing a face covering only in people who reported having been out shopping or to hospitality venues in the past week. Workplace attendance was investigated only in those who reported being able to fully work from home. This limited our sample size and our ability to detect small effects. Data are cross-sectional and we are unable to determine direction of associations. One complicating factor for our analyses was the national discussion around "partygate" that occurred at around the same time as the emergence of Omicron. A debate has developed over what, if any, effects the reporting about social events in No 10 had on public adherence.(34) We do not know if perceptions or behaviours might have been different, had reporting of these events not occurred at this time.

The Omicron variant emerged almost two years after the start of the COVID-19 outbreak. Despite substantial uncertainty about the impact of the resulting wave of infections, our data indicate that the emergence of the Omicron variant only slightly influenced worry about and perceived risk of COVID-19, suggesting a degree of habituation among the public to new announcements about the pandemic. Despite this, wearing a face covering – the main legislated change in response to Omicron – and uptake of testing increased between 1 November and 16 December 2021. These results suggest that specific behaviour changes continued to occur in response to changes in rules. Amount heard about Omicron was associated with always wearing a face covering, suggesting that communications emphasising protective behaviours may increase engagement for behaviours that are required by law still further.

For peer review only - http://bmjopen.bmj.com/site/about/guidelines.xhtml

Funding statement

This work was funded by the National Institute for Health Research (NIHR) Health Services and Delivery Research programme (NIHR project reference number (11/46/21)). Surveys were commissioned and funded by Department of Health and Social Care (DHSC), with the authors providing advice on the question design and selection. LS, RA and GJR are supported by the National Institute for Health Research Health Protection Research Unit (NIHR HPRU) in Emergency Preparedness and Response, a partnership between the UK Health Security Agency, King's College London and the University of East Anglia. RA is also supported by the NIHR HPRU in Behavioural Science and Evaluation, a partnership between the UK Health Security Agency and the University of Bristol. HWWP has received funding from Public Health England and NHS England. NTF is part funded by a grant from the UK Ministry of Defence. The views expressed are those of the authors and not necessarily those of the NIHR, UK Health Security Agency, the Department of Health and Social Care or the Ministry of Defence. The Department of Health and Social Care funded data collection (no grant number).

Competing interests statement

All authors have completed the ICMJE uniform disclosure form at www.icmje.org/coi_disclosure.pdf and declare: all authors had financial support from NIHR for the submitted work; RA is an employee of the UK Health Security Agency; HWWP received additional salary support from Public Health England and NHS England; HWWP receives consultancy fees to his employer from Ipsos MORI and has a PhD student who works at and has fees paid by Astra Zeneca; no other financial relationships with any organisations that might have an interest in the submitted work in the previous three years; no other relationships or activities that could appear to have influenced the submitted work. NTF is a participant of an independent group advising NHS Digital on the release of patient data. All authors are participants of the UK's Scientific Advisory Group for Emergencies or its subgroups.

Author contribution statement

All authors conceptualised the study and contributed to survey materials. LS completed analyses with guidance from HWWP and GJR. LS and GJR wrote the first draft of the manuscript. HWWP, RA, NTF, and SM contributed to subsequent drafts of the manuscript. LS, HWWP, RA, NTF, SM and GJR approved the final manuscript. GJR is guarantor. The corresponding author attests that all listed authors meet authorship criteria and that no others meeting the criteria have been omitted.

Data sharing statement

No additional data are available from the authors.

For peer review only

References

1. World Health Organization. Classification of Omicron (B.1.1.529): SARS-CoV-2 Variant of Concern. 2021. Available from: https://www.who.int/news/item/26-11-2021-classification-of-omicron-(b.1.1.529)-sars-cov-2-variant-of-concern.

2. Merrick J. Covid 'super variant' with 32 mutations found with cases in South Africa, Botswana and Hong Kong. iNews. 24 November 2021. Available from: https://inews.co.uk/news/politics/covid-super-variant-mutations-cases-found-south-africa-botswana-hong-kong-1316864.

3. Sky News. COVID-19: 'Really awful' new coronavirus variant B.1.1.529 identified in Botswana. 25 November 2021. Available from: https://news.sky.com/story/covid-19-new-coronavirus-variant-b-1-1-529-identified-in-botswana-as-scientists-play-down-concerns-12478164.

4. Smith LE, Potts HWW, Amlôt R, Fear N, Michie S, Rubin GJ. Worry and behaviour at the start of the COVID-19 outbreak: results from three UK surveys. Prev Med Rep. 2022;25(101686).

5. Prime Minister confirms move to Step 4 [press release]. 12 July 2021. Available from: https://www.gov.uk/government/news/prime-minister-confirms-move-to-step-4.

6. Davies R, Martin AF, Smith LE, Mowbray F, Woodland L, Amlot R, et al. The impact of "freedom day" on COVID-19 health protective behaviour in England: An observational study of hand hygiene, face covering use and physical distancing in public spaces pre and post the relaxing of restrictions. Open Science Framework. 2021. DOI [pre-print]: 10.17605/OSF.IO/CK2U4

7. First UK cases of Omicron variant identified [press release]. 2021. Available from: https://www.gov.uk/government/news/first-uk-cases-of-omicron-variant-identified.

8. Prime Minister sets out new measures as Omicron variant identified in UK: 27 November 2021 [press release]. 27 November 2021. Available from: https://www.gov.uk/government/news/prime-minister-sets-out-new-measures-as-omicron-variant-identified-in-uk-27-november-2021.

9. Measures against Omicron variant come into effect: 30 November 2021 [press release]. 30 November 2021. Available from: https://www.gov.uk/government/news/measures-against-omicron-variant-come-into-effect-30-november-2021.

10. Northern Ireland Department of Health. 'Lateral Flow before you go'. 2021. Available from: https://www.health-ni.gov.uk/news/lateral-flow-you-go.

11. Prime Minister's Office 10 Downing Street. Prime Minister confirms move to Plan B in England. 2021. Available from: https://www.gov.uk/government/news/prime-minister-confirms-move-to-plan-b-in-england.

12. Jessica Elgot, Nicola Davis. No new Covid restrictions in England before new year, Boris Johnson says. The Guardian. 27 December 2021. Available from: https://www.theguardian.com/world/2021/dec/27/boris-johnson-will-impose-no-further-covid-restrictions-before-new-year.

13. Reicher S, Drury J. Pandemic fatigue? How adherence to covid-19 regulations has been misrepresented and why it matters. BMJ Opinion. 2021.

For peer review only

14. Smith LE, Potts HWW, Amlôt R, Fear N, Michie S, Rubin GJ. How have patterns of social mixing changed during the pandemic? A series of cross-sectional nationally representative surveys. Open Science Framework. 2021. DOI [pre-print]: 10.17605/OSF.IO/DQA7Z

15. Rubin GJ, Potts HWW, Michie S. The impact of communications about swine flu (influenza A H1N1v) on public responses to the outbreak: results from 36 national telephone surveys in the UK. Health Technol Asses. 2010;14(34):183-266.

16. Rubin GJ, Amlot R, Page L, Wessely S. Public perceptions, anxiety, and behaviour change in relation to the swine flu outbreak: cross sectional telephone survey. BMJ. 2009;339:b2651.

17. Dryhurst S, Schneider CR, Kerr J, Freeman ALJ, Recchia G, van der Bles AM, et al. Risk perceptions of COVID-19 around the world. J Risk Res. 2020:1-13.

18. Slovic P, Fischhoff B, Lichtenstein S. Facts and fears, Understanding perceived risk. In: Schwing R, Albers Jr. WA, editors. How safe is safe enough? . New York: Plenum; 1980.

19. Smith LE, Potts HWW, Amlot R, Fear NT, Michie S, Rubin GJ. Adherence to the test, trace, and isolate system in the UK: results from 37 nationally representative surveys. BMJ. 2021;372:n608.

20. NHS. Who is at high risk from coronavirus (COVID-19). 2021. Available from: https://www.nhs.uk/conditions/coronavirus-covid-19/people-at-higher-risk/who-is-at-high-risk-from-coronavirus/.

21. Ministry of Housing Communities and Local Government. The English Indices of Deprivation 2019 (IoD2019). 2019. Available from: https://assets.publishing.service.gov.uk/government/uploads/system/uploads/attachment_data/file/835115/IoD2019_Statistical_Release.pdf.

22. Rubin GJ, Bakhshi S, Amlot R, Fear N, Potts HWW, Michie S. The design of a survey questionnaire to measure perceptions and behaviour during an influenza pandemic: the Flu TElephone Survey Template (FluTEST). Health Services and Delivery Research. 2014;2(41).

23. GOV.UK. Cases in England. 2022. Available from: https://coronavirus.data.gov.uk/details/cases?areaType=nation&areaName=England.

24. Smith LE, Potts HWW, Amlôt R, Fear N, Michie S, Rubin GJ. Perseverance not 'fatigue': A series of cross-sectional surveys of recommended behaviours during the COVID-19 pandemic in the UK. Open Science Framework. 2021. DOI [pre-print]: 10.17605/OSF.IO/FQKW8

25. Office for National Statistics. Coronavirus and the social impacts on Great Britain: 17 December 2021. 2021. Available from: https://www.ons.gov.uk/peoplepopulationandcommunity/healthandsocialcare/healthandwellbeing/bulletins/coronavirusandthesocialimpactsongreatbritain/17december2021.

26. UK Health Security Agency. Weekly national Influenza and COVID-19 surveillance report Week 50 report (up to week 49 data) 16 December 2021. 2021. Available from: https://assets.publishing.service.gov.uk/government/uploads/system/uploads/attachment_data/file/1041447/Weekly_Flu_and_COVID-19_report_w50.pdf.

27. Richard Partington. Dining out in UK at lowest level since May amid Omicron fears. The Guardian. 2 December 2021. Available from: https://www.theguardian.com/business/2021/dec/02/dining-out-in-uk-at-lowest-level-since-may-amid-omicron-fears.

For peer review only - http://bmjopen.bmj.com/site/about/guidelines.xhtml

28. Connor Ibbetson. Most Britons are carrying on as normal despite Omicron. 2021. Available from: https://yougov.co.uk/topics/lifestyle/articles-reports/2021/12/17/most-britons-are-carrying-normal-despite-omicron.

29. Williams SN, Armitage CJ, Tampe T, Dienes K. Public perceptions and experiences of social distancing and social isolation during the COVID-19 pandemic: a UK-based focus group study. BMJ Open. 2020;10(7):e039334.

30. Williams SN, Dienes K. 'Variant fatigue'? Public attitudes to COVID-19 18 months into the pandemic: A qualitative study. PsyArXiv. 2021. DOI [pre-print]: 10.31234/osf.io/vam4t

31. Smith LE, Amlôt R, Fear NT, Michie S, Rubin GJ, Potts HWW. Psychological wellbeing in the English population during the COVID-19 pandemic: a series of cross-sectional surveys. OSF. 2021. DOI [pre-print]: 10.17605/OSF.IO/U34F6

32. Smith LE, Duffy B, Moxham-Hall V, Strang L, Wessely S, Rubin GJ. Anger and confrontation during the COVID-19 pandemic: a national cross-sectional survey in the UK. J R Soc Med. 2021;114(2):77-90.

33. Cabinet Office. COVID-19 Response - Spring 2021 (Summary). 2021. Available from: https://www.gov.uk/government/publications/covid-19-response-spring-2021/covid-19-response-spring-2021-summary.

34. Reicher S. It is wrong to claim that the public won't follow covid rules just because the government don't. BMJ Opinion. 2021;375(n3150).

For peer review only

For peer review only - http://bmjopen.bmj.com/site/about/guidelines.xhtml

Figure 1. Perceived worry about, and risk of, COVID-19 between 1st November and 16th December 2021.

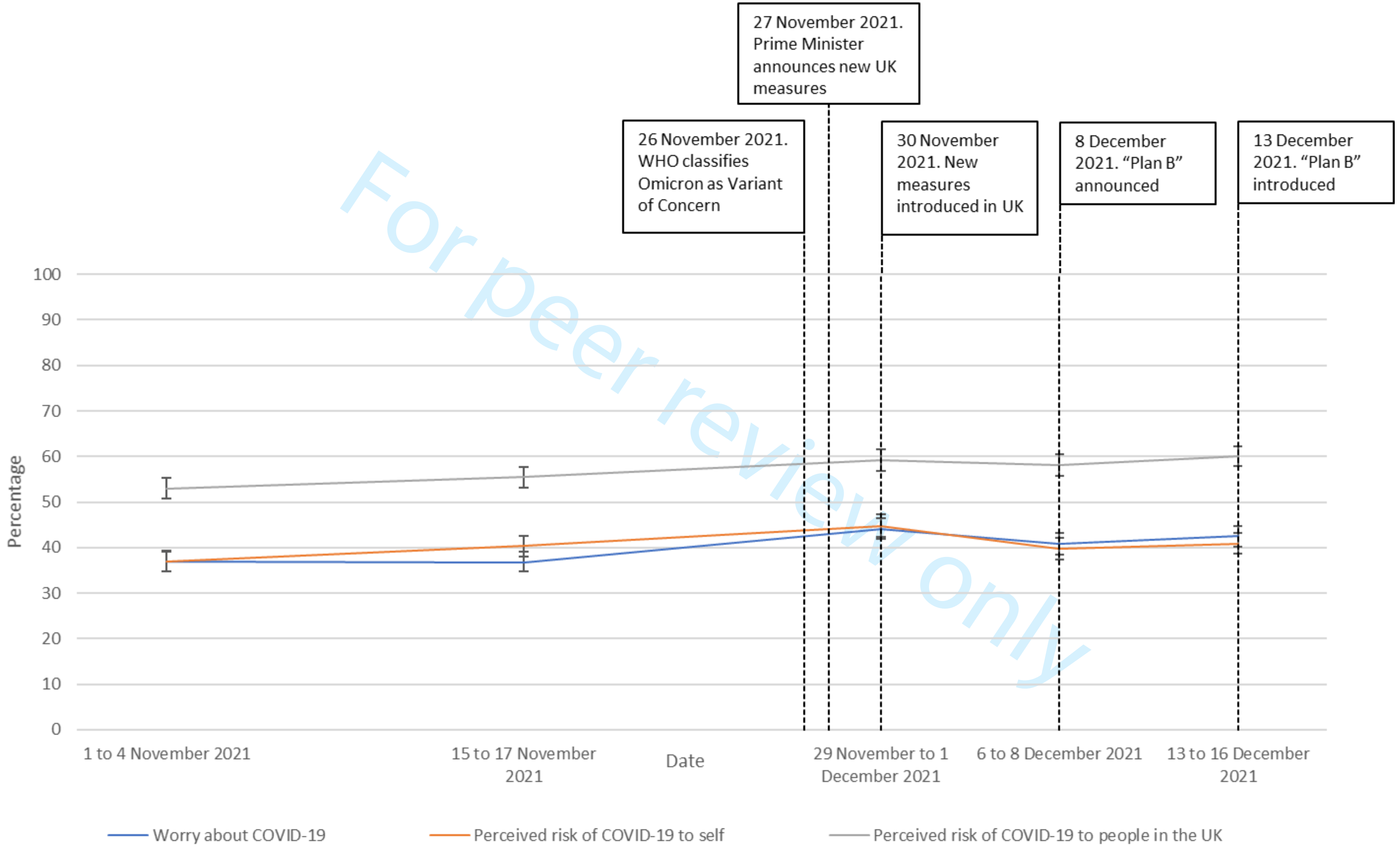

For peer review only

Figure 2. Out-of-home activity, between 1st November and 16th December 2021.

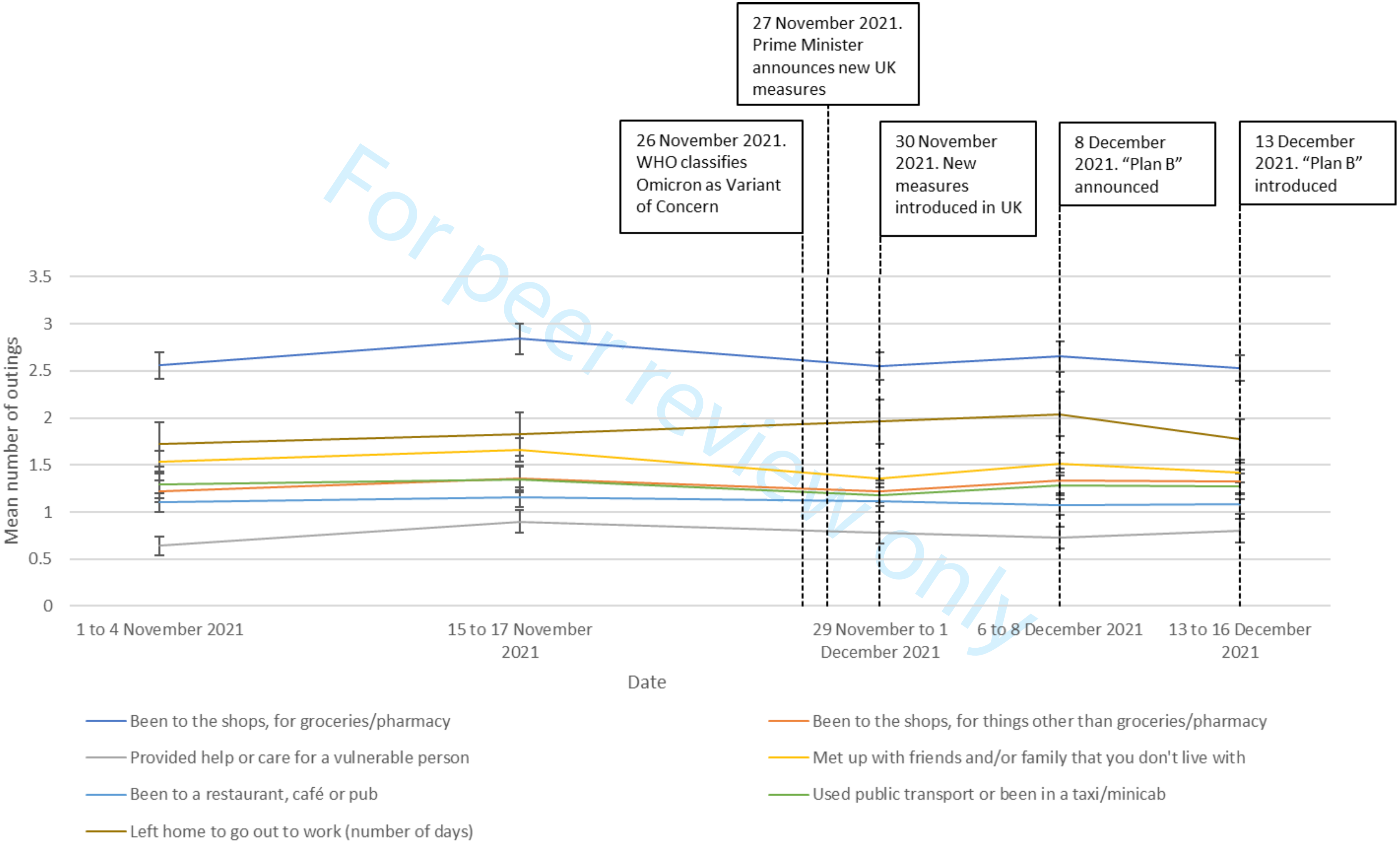

Figure 3. Risky social mixing, between 1st November and 16th December 2021.

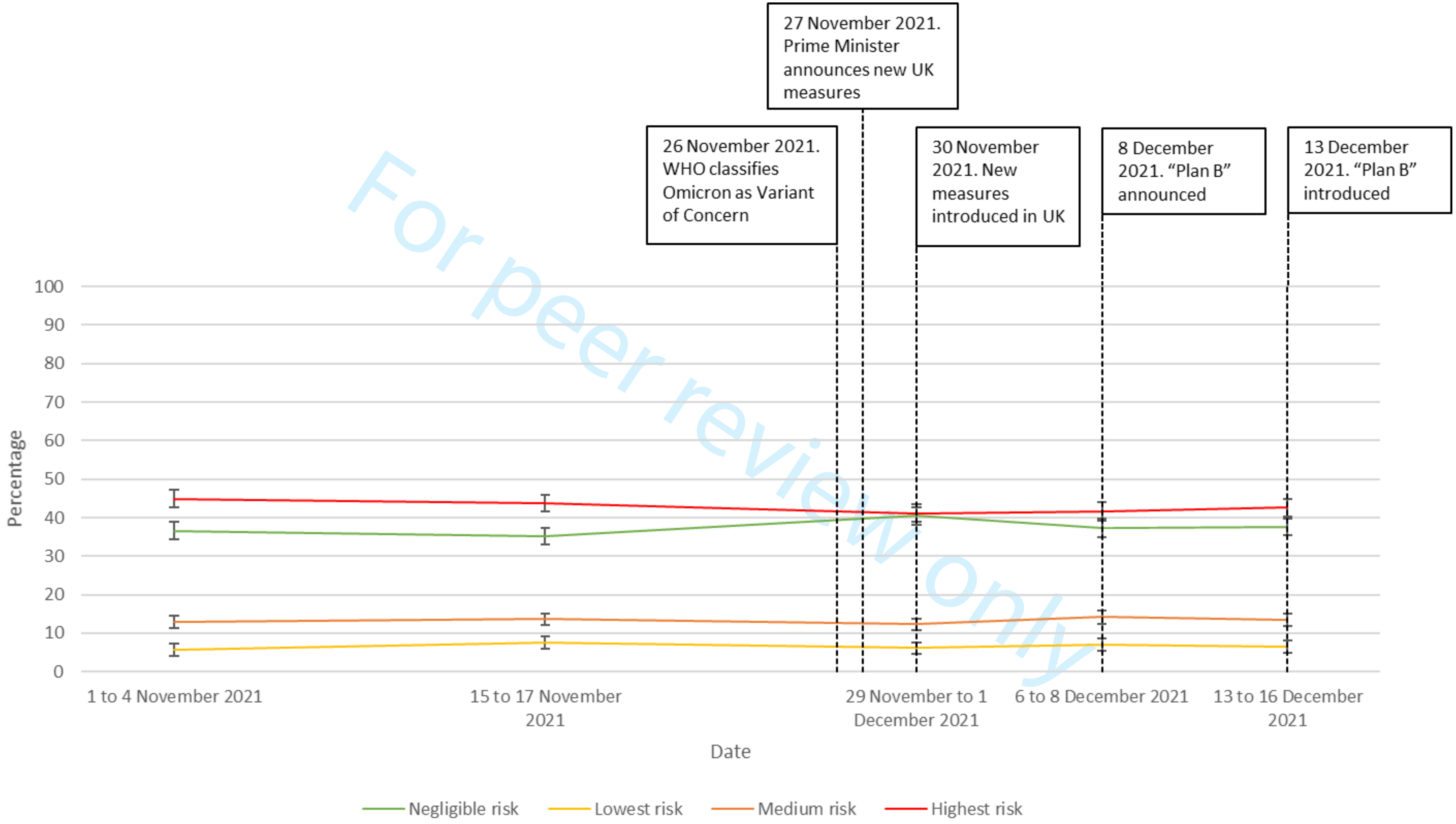

For peer review only

Figure 4. Always wearing a face covering, between 1st November and 16th December 2021.

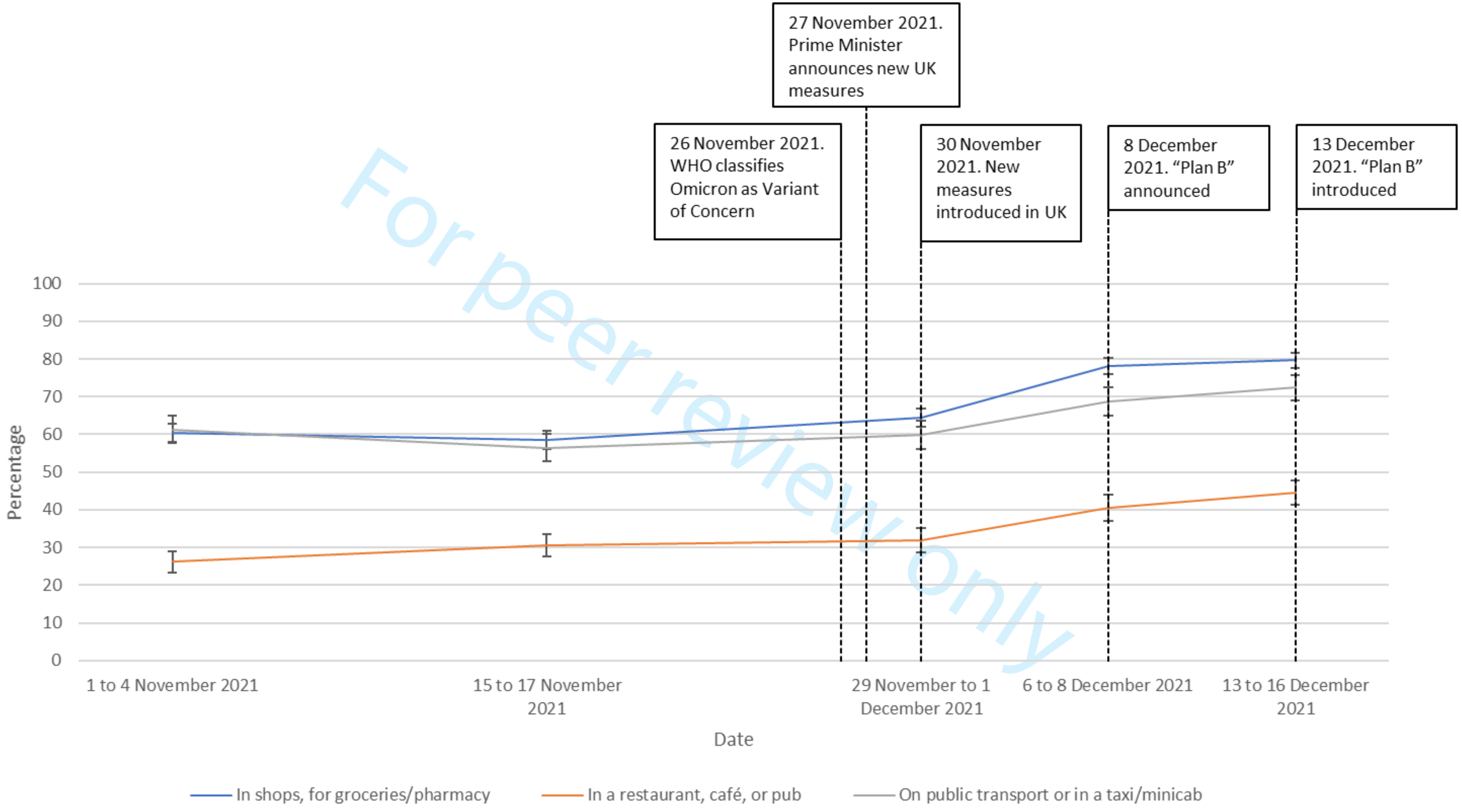

For peer review only

Figure 5. Uptake of testing, between 1st November and 16th December 2021. The dashed line shows the seven-day average for new cases in England.(22)

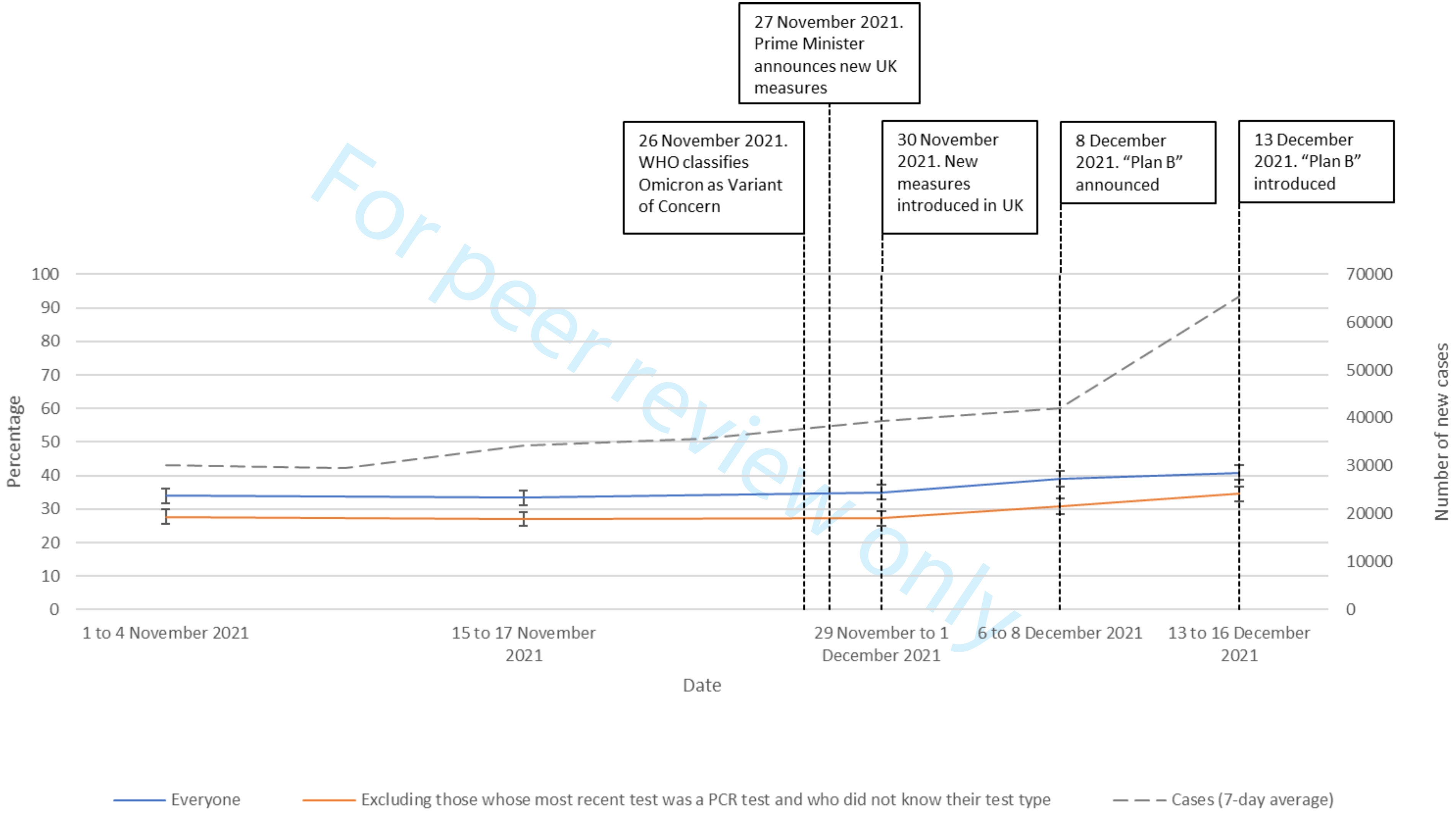

Supplementary materials

Figure 1. Timeline of announcements, data collection, and dates of self-reported behaviours. All dates 2021.

<table>
<tr><td>24-Nov</td><td>Omicron variant reported to WHO</td><td></td><td rowspan="9">Wave 63 “past seven days” could cover</td><td></td><td></td></tr>
<tr><td>25-Nov</td><td></td><td></td><td></td><td></td></tr>
<tr><td>26-Nov</td><td>Omicron designated a variant of concern</td><td></td><td></td><td></td></tr>
<tr><td>27-Nov</td><td>New measures announced
First UK cases</td><td></td><td></td><td></td></tr>
<tr><td>28-Nov</td><td></td><td></td><td></td><td></td></tr>
<tr><td>29-Nov</td><td></td><td rowspan="4">Wave 63</td><td rowspan="10">Wave 63.5 “past seven days” could cover</td><td></td></tr>
<tr><td>30-Nov</td><td>New measures take effect
Daily Mirror breaks ‘Partygate’</td><td></td></tr>
<tr><td>01-Dec</td><td></td><td></td></tr>
<tr><td>02-Dec</td><td></td><td></td></tr>
<tr><td>03-Dec</td><td>Omicron cases in the UK exceed 100</td><td></td><td></td><td></td></tr>
<tr><td>04-Dec</td><td></td><td></td><td></td><td></td></tr>
<tr><td>05-Dec</td><td></td><td></td><td></td><td></td></tr>
<tr><td>06-Dec</td><td></td><td rowspan="3">Wave 63.5</td><td></td><td rowspan="10">Wave 64 “past seven days” could cover</td></tr>
<tr><td>07-Dec</td><td>ITV reports on a video seeming to confirm ‘Partygate’</td><td></td></tr>
<tr><td>08-Dec</td><td>“Plan B” announced</td><td></td></tr>
<tr><td>09-Dec</td><td></td><td></td><td></td><td></td></tr>
<tr><td>10-Dec</td><td></td><td></td><td></td><td></td></tr>
<tr><td>11-Dec</td><td></td><td></td><td></td><td></td></tr>
<tr><td>12-Dec</td><td></td><td></td><td></td><td></td></tr>
<tr><td>13-Dec</td><td>“Plan B” mostly takes effect
First UK death from Omicron</td><td rowspan="3">Wave 64</td><td></td><td></td></tr>
<tr><td>14-Dec</td><td></td><td></td><td></td></tr>
<tr><td>15-Dec</td><td>UK records its highest number of daily cases (78,610)</td><td></td><td></td></tr>
</table>

For peer review only

16-Dec	UK records its highest number of daily cases (88,376)				

For peer review only

Table 1. Associations between out-of-home activities and socio-demographic factors, adjusting for all other socio-demographic factors. Bolding denotes significant findings (p<.002).

		Going out shopping (for groceries/pharmacy and other items) [block 1]				Attending the workplace [block 1]			
Attribute	Level	Wave 63.5 [a] aIRR for going out shopping (95% CI)	*p*	Wave 64 [b] aIRR for going out shopping (95% CI)	*p*	Wave 63.5 [c] aIRR for attending the workplace (95% CI)	*p*	Wave 64 [d] aIRR for attending the workplace (95% CI)	*p*
Region	East Midlands	Ref	-	Ref	-	Ref	-	Ref	-
	East of England	0.93 (0.72 to 1.21)	0.59	0.90 (0.70 to 1.15)	0.39	1.07 (0.53 to 2.16)	0.84	0.96 (0.53 to 1.73)	0.88
	London	1.10 (0.84 to 1.43)	0.51	1.08 (0.85 to 1.37)	0.54	0.87 (0.48 to 1.60)	0.66	1.14 (0.68 to 1.91)	0.61
	North East	1.03 (0.72 to 1.47)	0.85	0.96 (0.70 to 1.30)	0.77	0.79 (0.36 to 1.76)	0.57	1.08 (0.50 to 2.35)	0.84
	North West	1.09 (0.83 to 1.42)	0.54	1.05 (0.83 to 1.32)	0.68	1.10 (0.59 to 2.05)	0.77	0.90 (0.52 to 1.56)	0.72
	South East	0.99 (0.77 to 1.27)	0.93	0.93 (0.74 to 1.16)	0.51	0.90 (0.47 to 1.72)	0.75	1.11 (0.64 to 1.91)	0.71
	South West	0.98 (0.75 to 1.30)	0.91	1.05 (0.82 to 1.34)	0.72	1.48 (0.78 to 2.82)	0.23	1.51 (0.80 to 2.85)	0.21
	West Midlands	1.13 (0.86 to 1.49)	0.37	1.01 (0.79 to 1.29)	0.93	1.28 (0.68 to 2.42)	0.44	0.77 (0.44 to 1.37)	0.38
	Yorkshire and The Humber	1.38 (0.99 to 1.91)	0.05	0.88 (0.69 to 1.12)	0.30	1.19 (0.51 to 2.79)	0.69	0.67 (0.34 to 1.30)	0.23
	Overall	$\chi^2(8)=9.4$	0.31	$\chi^2(8)=6.5$	0.60	$\chi^2(8)=6.7$	0.57	$\chi^2(8)=7.7$	0.46
Sex	Male	Ref	-	Ref	-	Ref	-	Ref	-
	Female	0.84 (0.74 to 0.94)	0.004	0.94 (0.84 to 1.05)	0.27	0.71 (0.53 to 0.95)	0.02	0.73 (0.55 to 0.97)	0.03
Age (per decade)	Raw age	0.95 (0.90 to 0.99)	0.01	0.97 (0.94 to 1.01)	0.19	0.77 (0.64 to 0.92)	0.004	0.89 (0.77 to 1.03)	0.12
Age: quadratic $(\text{age-mean})^2$	-	1.0000 (0.9998 to 1.0002)	1.00	0.9999 (0.9997 to 1.0001)	0.36	0.9994 (0.9986 to 1.0002)	0.16	0.9997 (0.999 to 1.0005)	0.49
Dependent child in household	None	Ref	-	Ref	-	Ref	-	Ref	-
	Child present	1.06 (0.92 to 1.22)	0.39	1.14 (1.00 to 1.31)	0.04	1.35 (0.99 to 1.83)	0.06	1.19 (0.89 to 1.59)	0.24
At high risk (self)	No	Ref	-	Ref	-	Ref	-	Ref	-
	Yes	0.88 (0.75 to 1.02)	0.10	0.96 (0.83 to 1.10)	0.54	1.02 (0.72 to 1.45)	0.91	0.81 (0.55 to 1.18)	0.27
Household member has chronic illness	No	Ref	-	Ref	-	Ref	-	Ref	-
	Yes	0.95 (0.8 to 1.12)	0.55	1.14 (0.98 to 1.33)	0.09	1.11 (0.67 to 1.83)	0.69	0.94 (0.59 to 1.50)	0.79
Employment status	Not working	Ref	-	Ref	-	-	-	-	-
	Working	1.07 (0.92 to 1.24)	0.37	0.98 (0.85 to 1.12)	0.72	-	-	-	-
Socio-economic grade‡	AB	Ref	-	Ref	-	Ref	-	Ref	-
	C1C2	0.89 (0.77 to 1.03)	0.11	0.94 (0.82 to 1.08)	0.38	1.39 (1.04 to 1.87)	0.03	0.94 (0.70 to 1.27)	0.69
	DE	0.88 (0.74 to 1.05)	0.16	1.01 (0.86 to 1.18)	0.91	1.41 (0.87 to 2.31)	0.17	1.48 (0.93 to 2.34)	0.10
	Overall	$\chi^2(2)=2.9$	0.23	$\chi^2(2)=1.4$	0.51	$\chi^2(2)=5.3$	0.07	$\chi^2(2)=4.1$	0.13
Index of multiple deprivation	1st (least) to 4th quartile (most deprived)	0.95 (0.89 to 1.01)	0.08	0.95 (0.89 to 1.00)	0.05	0.97 (0.84 to 1.13)	0.73	0.85 (0.74 to 0.98)	0.03
Highest educational or professional qualification	Less than degree	Ref	-	Ref	-	Ref	-	Ref	-
	Degree or higher	0.94 (0.82 to 1.08)	0.38	1.01 (0.88 to 1.15)	0.92	0.86 (0.64 to 1.15)	0.31	0.64 (0.47 to 0.88)	0.01
Ethnicity	White British	Ref	-	Ref	-	Ref	-	Ref	-
	White other	1.22 (0.89 to 1.66)	0.21	0.76 (0.55 to 1.06)	0.11	1.30 (0.66 to 2.58)	0.45	0.95 (0.50 to 1.81)	0.87

For peer review only

	Black and minority ethnicity	0.88 (0.71 to 1.08)	0.21	1.06 (0.88 to 1.27)	0.55	1.01 (0.70 to 1.47)	0.96	0.97 (0.66 to 1.43)	0.89
	Overall	$\chi^2(2)=4.4$	0.11	$\chi^2(2)=3.9$	0.15	$\chi^2(2)=0.6$	0.75	$\chi^2(2)=0.0$	0.98
First language	Not English	Ref	-	Ref	-	Ref	-	Ref	-
	English	0.72 (0.55 to 0.94)	0.02	0.71 (0.54 to 0.92)	0.009	1.20 (0.72 to 2.00)	0.49	0.65 (0.39 to 1.07)	0.09
Ever had COVID-19	Think not	Ref	-	Ref	-	Ref	-	Ref	-
	Think yes	1.16 (1.01 to 1.33)	0.03	0.95 (0.84 to 1.09)	0.48	0.93 (0.68 to 1.26)	0.63	0.96 (0.71 to 1.29)	0.78
Vaccination status	Not vaccinated	Ref	-	Ref	-	Ref	-	Ref	-
	1 dose	1.18 (0.89 to 1.56)	0.26	1.14 (0.88 to 1.49)	0.32	1.40 (0.83 to 2.37)	0.21	1.54 (0.91 to 2.60)	0.11
	2 doses or more	0.97 (0.81 to 1.17)	0.78	0.80 (0.68 to 0.95)	0.01	0.95 (0.64 to 1.40)	0.78	1.12 (0.77 to 1.62)	0.57
	Overall	$\chi^2(2)=2.2$	0.33	$\chi^2(2)=11.9$	0.003	$\chi^2(2)=3.0$	0.23	$\chi^2(2)=2.7$	0.27
Financial hardship	Range 3 (least) to 15 (most)	1.01 (0.99 to 1.03)	0.19	**1.03 (1.02 to 1.05)**	**<0.001**	1.02 (0.97 to 1.07)	0.47	1.06 (1.01 to 1.10)	0.02

a) 1622 people were eligible for inclusion in analyses investigating going out shopping analyses. There were different amounts of missing data depending on variables included in the models, so n ranged between 1440 and 1491 for different models.

b) 1841 people were eligible for inclusion in analyses investigating going out shopping analyses. There were different amounts of missing data depending on variables included in the models, so n ranged between 1671 and 1713 for different models.

c) 372 people were eligible for inclusion in analyses investigating non-essential workplace attendance (sample limited to people who reported they could work entirely from home). Due to missing data, n included in analyses ranged between 349 and 354.

d) 410 people were eligible for inclusion in analyses investigating non-essential workplace attendance (sample limited to people who reported they could work entirely from home). Due to missing data, n included in analyses ranged between 379 and 389.

For peer review only

Table 2. Associations between highest risk social mixing and socio-demographic factors, adjusting for all other socio-demographic characteristics. Results reported are for block 1 of worry analyses. Bolding denotes significant findings (p<.002).

		Highest risk social mixing				Wearing a face covering in shops				Wearing a face covering in hospitality venues			
Attribute	Level	Wave 63.5 [a] aOR for engaging in highest risk social mixing (95% CI)	*p*	Wave 64 [b] aOR for engaging in highest risk social mixing (95% CI)		Wave 63.5 [c] aOR for wearing a face covering in shops (95% CI)	*p*	Wave 64 [d] aOR for wearing a face covering in shops (95% CI)	*p*	Wave 63.5 [e] aOR for wearing a face covering in hospitality venues (95% CI)	*p*	Wave 64 [f] aOR for wearing a face covering in hospitality venues (95% CI)	*p*
Region	East Midlands	Ref	-	Ref	-	Ref	-	Ref	-	Ref	-	Ref	-
	East of England	1.55 (0.97 to 2.50)	0.07	0.88 (0.57 to 1.38)	0.58	1.02 (0.53 to 1.98)	0.95	0.54 (0.25 to 1.16)	0.11	0.59 (0.28 to 1.22)	0.16	1.13 (0.59 to 2.19)	0.71
	London	1.36 (0.82 to 2.25)	0.24	0.95 (0.61 to 1.46)	0.80	1.10 (0.56 to 2.15)	0.78	0.48 (0.23 to 1.00)	0.05	0.52 (0.25 to 1.08)	0.08	0.90 (0.49 to 1.64)	0.72
	North East	1.49 (0.77 to 2.86)	0.24	1.31 (0.76 to 2.27)	0.33	1.00 (0.42 to 2.37)	1.00	0.45 (0.18 to 1.10)	0.08	0.48 (0.18 to 1.27)	0.14	2.03 (0.95 to 4.30)	0.07
	North West	1.23 (0.75 to 2.04)	0.41	1.18 (0.77 to 1.79)	0.44	0.85 (0.44 to 1.66)	0.63	**0.31 (0.15 to 0.62)**	**0.001**	0.44 (0.21 to 0.96)	0.04	0.66 (0.35 to 1.22)	0.18
	South East	1.57 (0.99 to 2.49)	0.05	1.17 (0.78 to 1.76)	0.46	0.92 (0.49 to 1.71)	0.79	0.49 (0.24 to 1.00)	0.05	0.50 (0.25 to 1.02)	0.06	1.24 (0.69 to 2.25)	0.47
	South West	1.32 (0.79 to 2.20)	0.29	1.03 (0.66 to 1.62)	0.89	1.00 (0.50 to 1.98)	1.00	0.43 (0.20 to 0.92)	0.03	0.65 (0.29 to 1.43)	0.28	1.26 (0.67 to 2.35)	0.48
	West Midlands	1.26 (0.76 to 2.09)	0.37	1.01 (0.65 to 1.59)	0.95	1.05 (0.53 to 2.08)	0.88	0.34 (0.16 to 0.71)	0.004	0.51 (0.24 to 1.08)	0.08	0.99 (0.51 to 1.92)	0.97
	Yorkshire and The Humber	1.32 (0.72 to 2.43)	0.36	1.21 (0.78 to 1.88)	0.39	1.20 (0.53 to 2.73)	0.67	0.33 (0.16 to 0.68)	0.003	0.25 (0.09 to 0.68)	0.007	1.21 (0.64 to 2.30)	0.56
	Overall	$\chi^2(8)=5.3$	0.72	$\chi^2(8)=5.1$	0.74	$\chi^2(8)=1.6$	0.99	$\chi^2(8)=15.2$	0.06	$\chi^2(8)=9.0$	0.34	$\chi^2(8)=12.4$	0.13
Sex	Male	Ref	-	Ref	-	Ref	-	Ref	-	Ref	-	Ref	-
	Female	1.40 (1.12 to 1.74)	0.003	1.34 (1.10 to 1.64)	0.004	**1.65 (1.22 to 2.22)**	**0.001**	1.37 (1.03 to 1.82)	0.03	1.34 (0.97 to 1.86)	0.08	1.46 (1.09 to 1.96)	0.01
Age (per decade)	Raw age	0.98 (0.90 to 1.06)	0.60	0.95 (0.88 to 1.02)	0.15	1.18 (1.05 to 1.32)	0.005	**1.22 (1.10 to 1.36)**	**<0.001**	1.02 (0.90 to 1.15)	0.75	1.06 (0.96 to 1.17)	0.28
Age: quadratic $(\text{age-mean})^2$	-	1.0000 (0.9996 to 1.0004)	0.95	1.0002 (0.9999 to 1.0006)	0.18	1.000 (0.9995 to 1.0006)	0.91	0.9998 (0.9993 to 1.0003)	0.38	0.9999 (0.9993 to 1.0005)	0.72	1.0006 (1.0001 to 1.0011)	0.03
Dependent child in household	None	Ref	-	Ref	-	Ref	-	Ref	-	Ref	-	Ref	-
	Child present	0.82 (0.63 to 1.06)	0.13	0.83 (0.65 to 1.05)	0.12	**0.56 (0.40 to 0.77)**	**<0.001**	0.93 (0.68 to 1.28)	0.67	0.96 (0.66 to 1.40)	0.83	1.67 (1.18 to 2.36)	0.004
At high risk (self)	No	Ref	-	Ref	-	Ref	-	Ref	-	Ref	-	Ref	-
	Yes	0.89 (0.67 to 1.18)	0.43	0.77 (0.59 to 1.00)	0.05	1.12 (0.76 to 1.65)	0.57	0.81 (0.56 to 1.16)	0.25	1.29 (0.86 to 1.96)	0.22	1.33 (0.91 to 1.94)	0.14
	No	Ref	-	Ref	-	Ref	-	Ref	-	Ref	-	Ref	-

For peer review only

Household member has chronic illness	Yes	0.83 (0.61 to 1.14)	0.25	1.07 (0.81 to 1.41)	0.65	0.84 (0.56 to 1.27)	0.41	0.85 (0.58 to 1.24)	0.40	0.98 (0.61 to 1.58)	0.93	1.45 (0.96 to 2.19)	0.08
Employment status	Not working	Ref	-	Ref	-	Ref	-	Ref	-	Ref	-	Ref	-
	Working	0.75 (0.57 to 0.98)	0.03	0.77 (0.60 to 0.99)	0.04	0.98 (0.68 to 1.41)	0.91	0.94 (0.67 to 1.34)	0.74	0.65 (0.43 to 0.98)	0.04	0.97 (0.67 to 1.41)	0.88
Socio-economic grade‡	AB	Ref	-	Ref	-	Ref	-	Ref	-	Ref	-	Ref	-
	C1C2	1.06 (0.81 to 1.38)	0.69	1.01 (0.79 to 1.29)	0.95	0.89 (0.62 to 1.27)	0.52	1.16 (0.80 to 1.68)	0.44	0.78 (0.54 to 1.13)	0.19	0.85 (0.60 to 1.2)	0.35
	DE	0.89 (0.65 to 1.22)	0.48	0.86 (0.65 to 1.15)	0.32	0.76 (0.50 to 1.17)	0.21	0.69 (0.46 to 1.03)	0.07	0.89 (0.56 to 1.43)	0.64	0.94 (0.61 to 1.43)	0.77
	Overall	$\chi^2(2)=1.4$	0.50	$\chi^2(2)=1.6$	0.46	$\chi^2(2)=1.6$	0.46	$\chi^2(2)=8.9$	0.01	$\chi^2(2)=1.8$	0.41	$\chi^2(2)=0.9$	0.63
Index of multiple deprivation	1st (least) to 4th quartile (most deprived)	1.02 (0.91 to 1.14)	0.71	1.04 (0.94 to 1.15)	0.47	1.01 (0.87 to 1.17)	0.92	1.07 (0.93 to 1.23)	0.36	0.99 (0.84 to 1.18)	0.93	1.04 (0.90 to 1.21)	0.57
Highest educational or professional qualification	Less than degree	Ref	-	Ref	-	Ref	-	Ref	-	Ref	-	Ref	-
	Degree or higher	1.07 (0.84 to 1.37)	0.58	1.10 (0.87 to 1.38)	0.45	1.18 (0.85 to 1.64)	0.33	1.27 (0.89 to 1.79)	0.18	1.02 (0.71 to 1.45)	0.92	1.39 (0.99 to 1.94)	0.06
Ethnicity	White British	Ref	-	Ref	-	Ref	-	Ref	-	Ref	-	Ref	-
	White other	1.13 (0.62 to 2.06)	0.68	0.99 (0.55 to 1.81)	0.98	1.16 (0.54 to 2.49)	0.71	0.84 (0.38 to 1.84)	0.66	0.53 (0.21 to 1.34)	0.18	1.62 (0.69 to 3.84)	0.27
	Black and minority ethnicity	0.70 (0.47 to 1.03)	0.07	0.82 (0.58 to 1.17)	0.28	1.40 (0.86 to 2.26)	0.18	1.67 (1.03 to 2.70)	0.04	1.48 (0.87 to 2.54)	0.15	1.42 (0.87 to 2.34)	0.16
	Overall	$\chi^2(2)=4.2$	0.12	$\chi^2(2)=1.3$	0.52	$\chi^2(2)=1.8$	0.40	$\chi^2(2)=5.7$	0.06	$\chi^2(2)=5.1$	0.08	$\chi^2(2)=2.4$	0.30
First language	Not English	Ref	-	Ref	-	Ref	-	Ref	-	Ref	-	Ref	-
	English	1.44 (0.85 to 2.45)	0.17	0.93 (0.56 to 1.53)	0.77	1.08 (0.58 to 2.01)	0.80	0.92 (0.47 to 1.80)	0.80	1.49 (0.73 to 3.05)	0.28	1.13 (0.57 to 2.21)	0.73
Ever had COVID-19	Think not	Ref	-	Ref	-	Ref	-	Ref	-	Ref	-	Ref	-
	Think yes	1.01 (0.78 to 1.30)	0.96	1.30 (1.03 to 1.65)	0.03	1.10 (0.79 to 1.53)	0.57	0.85 (0.62 to 1.18)	0.34	1.14 (0.78 to 1.64)	0.50	0.62 (0.45 to 0.87)	0.005
Vaccination status	Not vaccinated	Ref	-	Ref	-	Ref	-	Ref	-	Ref	-	Ref	-
	1 dose	0.71 (0.4 to 1.25)	0.24	0.62 (0.37 to 1.03)	0.07	2.59 (1.36 to 4.95)	0.004	**2.69 (1.51 to 4.79)**	**0.001**	**3.86 (1.87 to 7.96)**	**<0.001**	1.21 (0.62 to 2.37)	0.58
	2 doses or more	0.9 (0.64 to 1.27)	0.56	1.00 (0.73 to 1.37)	0.99	**2.31 (1.57 to 3.41)**	**<0.001**	**4.50 (3.08 to 6.56)**	**<0.001**	2.28 (1.35 to 3.87)	0.002	1.35 (0.84 to 2.16)	0.22
	Overall	$\chi^2(2)=1.4$	0.50	$\chi^2(2)=4.2$	0.12	**$\chi^2(2)=19.3$**	**<0.001**	**$\chi^2(2)=60.7$**	**<0.001**	**$\chi^2(2)=14.9$**	**0.001**	$\chi^2(2)=1.5$	0.47
Financial hardship	Range 3 (least) to 15 (most)	0.95 (0.92 to 0.98)	0.005	0.96 (0.92 to 0.99)	0.01	0.97 (0.93 to 1.02)	0.22	0.95 (0.90 to 0.99)	0.02	1.03 (0.97 to 1.08)	0.37	1.01 (0.96 to 1.06)	0.74

a) 1622 people were eligible for inclusion in highest risk social mixing analyses. There were different amounts of missing data depending on variables included in the models, so n ranged between 1439 and 1446 for different models.

For peer review only

b) 1841 people were eligible for inclusion in highest risk social mixing analyses. There were different amounts of missing data depending on variables included in the models, so n ranged between 1668 and 1689 for different models.

c) 1404 people were eligible for inclusion in wearing a face covering in shops analyses (sample limited to people who reported having been out shopping in the last week). Due to missing data, n included in analyses ranged between 1247 and 1266.

d) 1600 people were eligible for inclusion in wearing a face covering in shops analyses (sample limited to people who reported having been out shopping in the last week). There were different amounts of missing data depending on variables included in the models, so n ranged between 1454 and 1475 for different models.

e) 789 people were eligible for inclusion in wearing a face covering in hospitality venues analyses (sample limited to people who reported having been out to hospitality venues in the last week). Due to missing data, n included in analyses ranged between 700 and 713.

f) 894 people were eligible for inclusion in wearing a face covering in hospitality venues analyses (sample limited to people who reported having been out to hospitality venues in the last week). There were different amounts of missing data depending on variables included in the models, so n ranged between 817 and 829 for different models.

For peer review only

STROBE Statement—Checklist of items that should be included in reports of ***cross-sectional studies***

	Item No	Recommendation	Page No
**Title and abstract**	1	(*a*) Indicate the study's design with a commonly used term in the title or the abstract	1,2
		(*b*) Provide in the abstract an informative and balanced summary of what was done and what was found	2
**Introduction**			
Background/rationale	2	Explain the scientific background and rationale for the investigation being reported	4
Objectives	3	State specific objectives, including any prespecified hypotheses	4
**Methods**			
Study design	4	Present key elements of study design early in the paper	5
Setting	5	Describe the setting, locations, and relevant dates, including periods of recruitment, exposure, follow-up, and data collection	5
Participants	6	(*a*) Give the eligibility criteria, and the sources and methods of selection of participants	5
Variables	7	Clearly define all outcomes, exposures, predictors, potential confounders, and effect modifiers. Give diagnostic criteria, if applicable	5-7
Data sources/ measurement	8*	For each variable of interest, give sources of data and details of methods of assessment (measurement). Describe comparability of assessment methods if there is more than one group	*5-7*
Bias	9	Describe any efforts to address potential sources of bias	5-8
Study size	10	Explain how the study size was arrived at	5
Quantitative variables	11	Explain how quantitative variables were handled in the analyses. If applicable, describe which groupings were chosen and why	7-8
Statistical methods	12	(*a*) Describe all statistical methods, including those used to control for confounding	7-8
		(*b*) Describe any methods used to examine subgroups and interactions	N/A
		(*c*) Explain how missing data were addressed	7-8
		(*d*) If applicable, describe analytical methods taking account of sampling strategy	7-8
		(*e*) Describe any sensitivity analyses	N/A
**Results**			
Participants	13*	(a) Report numbers of individuals at each stage of study—eg numbers potentially eligible, examined for eligibility, confirmed eligible, included in the study, completing follow-up, and analysed	8
		(b) Give reasons for non-participation at each stage	N/A
		(c) Consider use of a flow diagram	Considered
Descriptive data	14*	(a) Give characteristics of study participants (eg demographic, clinical, social) and information on exposures and potential confounders	8
		(b) Indicate number of participants with missing data for each variable of interest	9-19
Outcome data	15*	Report numbers of outcome events or summary measures	9-19

For peer review only - http://bmjopen.bmj.com/site/about/guidelines.xhtml

Main results	16	(*a*) Give unadjusted estimates and, if applicable, confounder-adjusted estimates and their precision (eg, 95% confidence interval). Make clear which confounders were adjusted for and why they were included	9-19
		(*b*) Report category boundaries when continuous variables were categorized	Reported
		(*c*) If relevant, consider translating estimates of relative risk into absolute risk for a meaningful time period	Considered
Other analyses	17	Report other analyses done—eg analyses of subgroups and interactions, and sensitivity analyses	9-19
**Discussion**			
Key results	18	Summarise key results with reference to study objectives	20-21
Limitations	19	Discuss limitations of the study, taking into account sources of potential bias or imprecision. Discuss both direction and magnitude of any potential bias	21
Interpretation	20	Give a cautious overall interpretation of results considering objectives, limitations, multiplicity of analyses, results from similar studies, and other relevant evidence	20-21
Generalisability	21	Discuss the generalisability (external validity) of the study results	20-21
**Other information**			
Funding	22	Give the source of funding and the role of the funders for the present study and, if applicable, for the original study on which the present article is based	22

*Give information separately for exposed and unexposed groups.

Note: An Explanation and Elaboration article discusses each checklist item and gives methodological background and published examples of transparent reporting. The STROBE checklist is best used in conjunction with this article (freely available on the Web sites of PLoS Medicine at http://www.plosmedicine.org/, Annals of Internal Medicine at http://www.annals.org/, and Epidemiology at http://www.epidem.com/). Information on the STROBE Initiative is available at www.strobe-statement.org.

For peer review only

BMJ Open

How has the emergence of the Omicron SARS-CoV-2 variant of concern influenced worry, perceived risk, and behaviour in the UK? A series of cross-sectional surveys

Journal:	*BMJ Open*
Manuscript ID	bmjopen-2022-061203.R1
Article Type:	Original research
Date Submitted by the Author:	26-May-2022
Complete List of Authors:	Smith, Louise; King's College London<br>Potts, Henry; University College London, Institute of Health Informatics<br>Amlot, Richard; UK Health Security Agency<br>Fear, Nicola; King's College London, King's Centre for Military Health Research and Academic Department of Military Mental Health<br>Michie, Susan; University College London, Centre for Behaviour Change<br>Rubin, G James; King's College London, Institute of Psychiatry, Psychology and Neuroscience
**Primary Subject Heading**:	Public health
Secondary Subject Heading:	Infectious diseases
Keywords:	COVID-19, Public health < INFECTIOUS DISEASES, PUBLIC HEALTH

SCHOLARONE™
Manuscripts

I, the Submitting Author has the right to grant and does grant on behalf of all authors of the Work (as defined in the below author licence), an exclusive licence and/or a non-exclusive licence for contributions from authors who are: i) UK Crown employees; ii) where BMJ has agreed a CC-BY licence shall apply, and/or iii) in accordance with the terms applicable for US Federal Government officers or employees acting as part of their official duties; on a worldwide, perpetual, irrevocable, royalty-free basis to BMJ Publishing Group Ltd ("BMJ") its licensees and where the relevant Journal is co-owned by BMJ to the co-owners of the Journal, to publish the Work in this journal and any other BMJ products and to exploit all rights, as set out in our licence.

The Submitting Author accepts and understands that any supply made under these terms is made by BMJ to the Submitting Author unless you are acting as an employee on behalf of your employer or a postgraduate student of an affiliated institution which is paying any applicable article publishing charge ("APC") for Open Access articles. Where the Submitting Author wishes to make the Work available on an Open Access basis (and intends to pay the relevant APC), the terms of reuse of such Open Access shall be governed by a Creative Commons licence – details of these licences and which Creative Commons licence will apply to this Work are set out in our licence referred to above.

Other than as permitted in any relevant BMJ Author's Self Archiving Policies, I confirm this Work has not been accepted for publication elsewhere, is not being considered for publication elsewhere and does not duplicate material already published. I confirm all authors consent to publication of this Work and authorise the granting of this licence.

For peer review only

How has the emergence of the Omicron SARS-CoV-2 variant of concern influenced worry, perceived risk, and behaviour in the UK? A series of cross-sectional surveys

Louise E Smith (0000-0002-1277-2564),[1,2] PhD, Henry WW Potts (0000-0002-6200-8804),[3] PhD, Richard Amlôt (0000-0003-3481-6588),[2,4] PhD, Nicola T Fear (0000-0002-5792-2925),[1,5] DPhil (Oxon), Susan Michie (0000-0003-0063-6378),[6] DPhil, G James Rubin (0000-0002-4440-0570),[1,2] PhD

1 King's College London, Institute of Psychiatry, Psychology and Neuroscience

2 NIHR Health Protection Research Unit in Emergency Preparedness and Response

3 University College London, Institute of Health Informatics

4 UK Health Security Agency, Behavioural Science and Insights Unit

5 King's Centre for Military Health Research and Academic Department of Military Mental Health, King's College London

6 University College London, Centre for Behaviour Change

Corresponding author: Louise E Smith, Post-doctoral Researcher. Department of Psychological Medicine, King's College London, Weston Education Centre, Cutcombe Road, London, SE5 9RJ. Email: louise.e.smith@kcl.ac.uk. ORCID number: 0000-0002-1277-2564. Twitter handle: @louisesmith142

Henry WW Potts, Professor of Health Informatics. Institute of Health Informatics, 222 Euston Road, London, NW1 2DA.

Richard Amlôt, Head of Behavioural Science in the Behavioural Science and Insights Unit at the UK Health Security Agency. Porton Down, Salisbury, Wiltshire, SP4 0JG.

Nicola T Fear, Professor of Epidemiology. Department of Psychological Medicine, King's College London, Weston Education Centre, Cutcombe Road, London, SE5 9RJ.

Susan Michie, Professor of Health Psychology. Centre for Behaviour Change, University College London,1-19 Torrington Place, London, WC1E 7HB.

G James Rubin, Assistant Director, NIHR Health Protection Research Unit in Emergency Preparedness and Response, King's College London, Weston Education Centre, Cutcombe Road, London, SE5 9RJ.

Manuscript word count: 4599

For peer review only - http://bmjopen.bmj.com/site/about/guidelines.xhtml

Abstract

Objectives: To investigate changes in beliefs and behaviours following news of the Omicron variant and changes to guidance understanding of Omicron-related guidance, and factors associated with engaging with protective behaviours.

Design: Series of cross-sectional surveys (1 November to 16 December 2021, 5 waves of data collection).

Setting: Online.

Participants: People living in England, aged 16 years or over (n=1622 to 1902 per wave).

Primary and secondary outcome measures: Levels of worry and perceived risk, and engagement with key behaviours (out-of-home activities, risky social mixing, wearing a face covering, and testing uptake).

Results: Degree of worry and perceived risk of COVID-19 (to oneself, and people in the UK) fluctuated over time, increasing slightly around the time of the announcement about Omicron ($p<0.001$). Understanding of rules in England was varied, ranging between 20.2% and 91.9%, with people over-estimating the stringency of the new rules. Rates of wearing a face covering and testing increased over time ($p<0.001$). Meeting up with people from another household decreased around the time of the announcement of Omicron (29 November to 1 December), but then returned to previous levels ($p=0.002$). Associations with protective behaviours were investigated using regression analyses. There was no evidence for significant associations between out-of-home activity and worry or perceived risk (COVID-19 generally or Omicron-specific, $p>0.004$; Bonferroni adjustment $p<0.002$ applied). Engaging in highest risk social mixing and always wearing a face covering were associated with worry and perceived risk about COVID-19 ($p>=0.001$). Always wearing a face covering in shops was associated with having heard more about Omicron ($p<0.001$).

Conclusions: Almost two years into the COVID-19 outbreak, the emergence of a novel variant of concern only slightly influenced worry and perceived risk. The main protective behaviour (wearing a face covering) promoted by new guidance showed significant re-uptake, but other protective behaviours showed little or no change.

Abstract word count: 300

Key words: COVID-19, variant of concern, perceptions, behaviour, non-pharmaceutical interventions, public health and social measures

For peer review only - http://bmjopen.bmj.com/site/about/guidelines.xhtml

Strengths and limitations of this study

- Rapid data collection, reporting on beliefs and behaviours immediately following news of the emergence of the Omicron variant of concern.
- Large sample size, and repeated questions, allow for precise prevalence estimates and investigation of longer-term trends.
- Data are self-reported and may therefore represent an overestimation of engagement with protective behaviours.
- Data are cross-sectional, and we cannot imply the direction of associations.
- We are unsure of the representativeness of the beliefs and behaviours of people who sign up to take part in online surveys.

For peer review only

For peer review only - http://bmjopen.bmj.com/site/about/guidelines.xhtml

Introduction

The Omicron variant of SARS-CoV-2 was reported to the World Health Organization (WHO) on 24 November 2021 and was designated by the WHO as a variant of concern on 26 November 2021.(1) Since this date, it has attracted substantial media coverage.(2, 3) The emergence of the Omicron variant presented policymakers, and society more generally, with a dilemma. What action should be taken in the face of a rapidly spreading infection, the severity of which is unclear? The UK has witnessed intense debate around this question, with disagreements being played out across the national press, in the House of Commons and in academic articles. In the early stages of the COVID-19 pandemic, the emergence of the original SARS-CoV-2 virus prompted similar controversy and led to modest increases in levels of worry among the UK public, with 40% engaging in recommended respiratory and hand hygiene behaviours, and 14% reducing the number of people that they met, a behaviour that had not then been officially recommended.(4)

England removed legal COVID-19 mandations to wear a face covering and physically distance on 19 July 2021.(5) This was followed by decreases in rates of protective behaviour.(6) In response to the Omicron variant, the UK Prime Minister, English Chief Medical Officer and Government Chief Scientific Advisor held a press conference on 27 November, the same day the first UK cases were reported,(7) in which new measures were announced.(8) These were implemented from 30 November 2021.(9) They included making face coverings compulsory in shops and on public transport, and requiring all international arrivals to take a polymerase chain reaction (PCR) test within two days of arriving in the UK and self-isolating until they received a negative test result.(5, 6) Recommendations for all members of the public to use lateral flow tests regularly, and before meeting other people (epitomised by the slogan “lateral flow before you go” used in the Devolved Administrations (10)) were retained and reiterated.

As more evidence about the rapid spread of the Omicron variant appeared, on 8 December 2021, further measures were announced as part of the UK’s “Plan B”, with face coverings becoming compulsory in most public indoor venues (apart from hospitality), vaccine passports becoming mandatory in specific settings and people being asked to work from home where possible.(11) These changes came into effect on 13 December 2021. On 27 December, the Government announced no new restrictions for England before the end of the year.(12)

Throughout the pandemic, concern has been raised that public adherence to rules may wane over time.(13) Nonetheless, changes in rules have consistently caused changes in behaviour.(14) Research conducted during the COVID-19 and the 2009 H1N1 pandemics indicated that engagement with protective behaviours was associated with having heard more about the pandemic,(4, 15) and increased worry about, and perceived risk of, infection.(16, 17) Public fears are known to be greater when risks are novel and uncertain.(18) While the risks of COVID-19 were familiar to the public, the new variant represented a possible new source of public worry that may have affected behaviour.

In this study, we investigated whether beliefs about COVID-19 and engagement with protective behaviours changed in the first three weeks of the emergence of the Omicron variant. We measured understanding of new guidance and satisfaction with the government response to Omicron. We also investigated whether engaging with protective behaviours was associated with amount heard about Omicron, worry (about COVID-19 generally and Omicron specifically), and perceived risk (of COVID-19 generally and Omicron specifically).

For peer review only

For peer review only - http://bmjopen.bmj.com/site/about/guidelines.xhtml

Methods

Design

A series of online cross-sectional surveys have been conducted by Savanta (a Market Research Society company partner) since January 2020 on behalf of the English Department of Health and Social Care, and analysed by the CORSAIR (COVID-19 Rapid Survey of Adherence to Interventions and Responses) research team.(19) For these analyses, we used data collected in five waves: wave 61 (1-4 November 2021), wave 62 (15-17 November), wave 63 (29 November-2 December), an *ad hoc* wave added to the series to assess responses to Omicron (6-8 December 2021; wave 63.5), and wave 64 (13-16 December).

Questions in each wave asked about behaviour over the previous week. Data collection for wave 63 took place after the first news about Omicron and the announcement of new rules. It spanned a longer period before (8 days), and a shorter period after (3 days), the rules came into force (30 November 2021; see supplementary materials Figure 1 for a timeline). The added survey (wave 63.5) was issued after the emergence of Omicron, but encompassed a shorter period before (1 day), and a longer period after (9 days), the new rules came into force. Wave 64 data collection started on the same day as further rules ("Plan B") came into force (13 December 2021; rules announced on 8 December). Wave 64 data therefore encompasses a longer period before (7 days), and after shorter period after (4 days), Plan B rules came into force.

Participants

Participants were recruited from a pool of people who had signed up to take part in online surveys (known as online research panels). Participants were eligible to take part if they were aged 16 years or over and lived in the UK. Non-probability sampling (quotas based on age and sex [combined], and region) was used to ensure the sample was broadly similar to the UK general population. After completing the survey, participants were unable to take part in the subsequent three waves of data collection. Participants were reimbursed in points which could be redeemed in cash, gift vouchers or charitable donations (up to 70p per survey).

We report figures for England only as the four nations of the UK implemented different changes for Omicron. We excluded participants in Wave 63.5 who completed the survey after the 8 December Government press conference began (n=58).

Study materials

Unless otherwise specified, participants answered all items.

Worry and perceived risk

Participants were asked "overall, how worried are you about coronavirus" on a five-point scale from "not at all worried" to "extremely worried". They were also asked "to what extent you think coronavirus poses a risk to…" them personally and people in the UK, on a five-point scale from "no risk at all" to "major risk". From wave 63.5, participants were also asked congruent questions about their worry about, and perceived risk of, Omicron. The items asked participants "Thinking about the Omicron variant, how worried are you about this specific variant of coronavirus?" and "to what extent you think this specific variant of coronavirus poses a risk…".

Worry and perceived risk (to oneself, others in the UK) were coded into separate binary variables (worry: very and extremely worried, versus somewhat, not very, and not at all worried; perceived risk: major and significant risk, versus moderate, minor, and no risk at all).

For peer review only - http://bmjopen.bmj.com/site/about/guidelines.xhtml

For peer review only

Behaviours

Participants were asked how many times in the last week they had done each of a list of 20 activities including shopping for groceries/pharmacy, shopping for other items, providing help or care for a vulnerable person, meeting up with friends or family that they did not live with, going to a restaurant, café or pub, using public transport or a taxi/minicab, and going out to work (number of days). Responses were capped at 30; going out to work was capped at 7.

Participants who indicated that they had met up with friends or family from another household were asked a series of follow-up questions about the setting and number of people involved in their most recent meeting in the past seven days. We derived a measure categorising the risk of transmission involved in a participant's most recent instance of social mixing.(14) We were unable to calculate this measure for five participants due to missing data.

Participants who indicated that they had visited a shop, hospitality venue, or used public transport or a minicab were asked whether they wore a face covering while doing so. Response options were "yes – on all occasions", "yes – on some occasions", and "no, not at all". We categorised people as wearing a face covering all the time, versus sometimes or not at all.

We asked participants when they last took a test for coronavirus. We categorised people as having tested if they indicated that they took their most recent test in the last week.

Amount heard about Omicron

From wave 63.5, participants were asked to indicate "how much, if anything, have you seen or heard about the new Omicron variant of coronavirus that was first detected in southern Africa?" on a four-point scale from "I have not seen or heard anything" to "I have seen or heard a lot".

Satisfaction with Government response

Participants in wave 63.5 onwards were asked to what extent they agreed or disagreed that "The Government was putting the right measures in place to protect the UK public from the Omicron variant of coronavirus", you "have enough information from the Government and other public authorities on the symptoms associated with the Omicron variant of coronavirus", and you "have enough information from the Government and other public authorities on how effective current vaccines are against the Omicron variant of coronavirus" on a five-point scale from strongly agree to strongly disagree.

Understanding of new rules

From wave 63.5, participants living in England were asked to indicate whether a series of nine statements about rules brought in to prevent the spread of Omicron were true, false, or they did not know. A tenth statement was added for wave 64. Statements included items about wearing a face covering in different locations (in shops, on public transport, in hospitality venues), self-isolation, and out-of-home behaviour.

Socio-demographic factors

We measured participants' age in years, sex, employment status, socio-economic grade, highest educational or professional qualification, ethnicity, their first language, COVID-19 vaccination status, whether there was a dependent child in the household, whether they were at high risk for COVID-19,(20) whether a household member had a chronic illness, and whether they thought they had previously, or currently, had COVID-19 (recoded to a binary variable: "I've definitely had it, and had

For peer review only - http://bmjopen.bmj.com/site/about/guidelines.xhtml

it confirmed by a test" and "I think I've probably had it", vs "I don't know whether I've had it or not", "I think I've probably not had it", and "I've definitely not had it"). Participants were also asked to report their full postcode, from which geographical region and indices of multiple deprivation were determined.(21)

To measure financial hardship, participants were asked to what extent in the past seven days they had been struggling to make ends meet, skipping meals they would usually have, and were finding their current living situation difficult (Cronbach's α=.84).

Patient and public involvement

Lay members served on the advisory group for the project that developed our prototype survey material; this included three rounds of qualitative testing.(22) Due to the rapid nature of this research, the public was not involved in the further development of the materials during the COVID-19 pandemic.

Power

A sample size of 1,600 per wave allows a 95% confidence interval of approximately plus or minus 2% for the prevalence estimate for a survey item with an overall prevalence of 50%.

Analysis

Unless otherwise specified, answers of "don't know" were coded as missing.

We limited analyses investigating non-essential workplace attendance to participants who reported being in in full-, part-, or self-employment, and who indicated that they could work from home full-time. Questions about wearing a face covering were only asked to people who reported having completed that activity in the past seven days. Therefore, analyses were restricted to those who reported having been in shops, on public transport, and in hospitality venues in the last week.

We plotted worry and perceived risk, and behaviours by survey wave. For uptake of testing, we plotted two lines, including and excluding those whose most recent test was a polymerase chain reaction (PCR) test and who did not know their most recent test type. To investigate change over time, we used χ^2 analyses (categorical data), one-way ANOVAs (continuous data), and Kruskal-Wallis tests (skewed continuous data).

We present descriptive statistics of participants' understanding of the new rules brought in in response to Omicron and satisfaction with the Government response.

To investigate associations with engagement with protective behaviours, we used data collected 6 to 8 December 2021 (wave 63.5) and 13 to 16 December (wave 64) separately as we hypothesised that people's views and behaviour were likely to change due to the fast-moving nature of the spread of Omicron. We used negative binomial regression analyses (to account for skewed outcomes) to investigate associations with out-of-home activities (going out shopping, going to the workplace). For these analyses, we summed the number of times participants reported going out shopping for groceries/pharmacy and other items, to give a total number of times gone shopping. We ran one model including only socio-demographic factors; a second that additionally included amount heard about Omicron, and perceived worry about COVID-19, perceived risk of COVID-19 to oneself, or perceived risk of COVID-19 to people in the UK; and a third that additionally included Omicron-specific worry or perceived risk. For these analyses, we report adjusted incidence rate ratios (aIRR).

For peer review only - http://bmjopen.bmj.com/site/about/guidelines.xhtml

For binary outcomes (risky social mixing: highest risk social mixing, vs other; always wearing a face covering in shops: wearing a face covering on all occasions, vs other; wearing a face covering in hospitality venues: wearing a face covering on all occasions, vs other), we used logistic regression analyses. Socio-demographic factors were entered as block one. Amount heard about Omicron and either worry about COVID-19, perceived risk of COVID-19 to oneself, or perceived risk of COVID-19 to people in the UK were entered as block two. Omicron-specific worry, perceived risk to self or perceived risk to people in the UK were entered as block three. For these analyses, we report adjusted odds ratios (aOR).

To account for the large number of analyses, we used a Bonferroni correction. For analyses investigating changes in beliefs and behaviour over time, we set significance at p<0.003 (n=22). For regression analyses, we set significance at p<0.002 (n=28).

Results

Respondent characteristics

8941 responses were included in analyses (wave 61, n=1833; wave 62, n=1902; wave 63, n=1743; wave 63.5, n=1622; wave 64, n=1841). Respondents were slightly more likely to be women, white, be educated to degree level or higher compared to the general population (Table 1).(23, 24) There was a significant difference in uptake of vaccination ($\chi^2(8)=17.0$, $p=0.03$). In practice, there were small differences between waves, with percentages differing at most by 4.2%.

Table 1. Respondent characteristics

Attribute	Level	Wave 61, % (n) [total n=1833]	Wave 62, % (n) [total n=1902]	Wave 63, % (n) [total n=1743]	Wave 63.5, % (n) [total n=1622]	Wave 64, % (n) [total n=1841]	*p*
Sex	Male	46.8 (853)	47.2 (893)	46.7 (812)	45.8 (741)	47.0 (862)	0.94
	Female	53.2 (968)	52.8 (999)	53.3 (925)	54.2 (878)	53.0 (973)	
Age	Range 16 to >90 years	M=48.7, SD=19.2	M=47.8, SD=18.8	M=49.1, SD=18.2	M=47.7, SD=18.4	M=47.7, SD=18.8	0.07
Employment status	Not working	46.8 (844)	44.8 (840)	45.5 (786)	44.0 (707)	44.7 (813)	0.54
	Working	53.2 (959)	55.2 (1033)	54.5 (943)	56.0 (899)	55.3 (1005)	
Index of multiple deprivation	1st (least) to 4th quartile (most deprived)	M=2.7, SD=1.0	M=2.7, SD=1.0	M=2.7, SD=1.0	M=2.8, SD=1.0	M=2.7, SD=1.0	0.62
Highest educational or professional qualification	Less than degree	65.4 (1198)	67.1 (1277)	66.8 (1165)	65.9 (1069)	67.5 (1243)	0.63
	Degree or higher	34.6 (635)	32.9 (625)	33.2 (578)	34.1 (553)	32.5 (598)	
Ethnicity	White British	82.2 (1498)	82.7 (1563)	84.2 (1460)	82.4 (1329)	82.0 (1505)	0.09
	White other	6.1 (111)	5.1 (96)	5.5 (96)	5.1 (82)	4.5 (83)	
	Black and minority ethnicity	11.7 (214)	12.2 (231)	10.2 (177)	12.5 (202)	13.5 (247)	
Vaccination status	Not vaccinated	10.7 (195)	14.4 (269)	13.1 (226)	13.4 (215)	13.6 (248)	0.03
	1 dose	5.4 (99)	6.0 (112)	5.2 (89)	6.5 (104)	6.3 (115)	
	2 doses or more	83.9 (1528)	79.7 (1493)	81.7 (1411)	80.2 (1291)	80.1 (1463)	

Where percentages do not sum to 100%, this is due to rounding errors.

Beliefs and behaviours over time

Perceived worry about, and risk of, COVID-19 fluctuated over time, with worry, perceived risk to self and perceived risk to people increasing slightly around the time of the announcement about the Omicron variant, then returning to pre-Omicron levels (worry ($F(4,8921)=10.08$, $p<.001$); perceived risk to self ($F(4,8857)=7.10$, $p<.001$); perceived risk to people in UK ($F(4,8854)=5.12$, $p<.001$); Figure 1).

For peer review only - http://bmjopen.bmj.com/site/about/guidelines.xhtml

Between 1 November and 16 December 2021, reported rates of meeting up with people from another household changed ($H(4)=17.4$, n=8941, $p=0.002$; Figure 2). This change was driven by a decrease in reported rates in data collected on 29 November to 1 December 2021 (wave 63, around the time of the announcement of Omicron) compared to the previous survey wave. Providing help or care for a vulnerable person also changed between 1 November and 16 December 2021 ($H(4)=17.0$, n=8941, $p=0.002$), with this change being driven by an increase in reported rates in data collected on 15 to 17 November 2021 compared to the previous survey wave. There were no other significant changes in out-of-home activity over time (been to the shops, for groceries/pharmacy ($H(4)=7.5$, n=8941, $p=0.11$); been to the shops, for things other than groceries/pharmacy ($H(4)=8.4$, n=8941, $p=0.08$); been to a restaurant, café or pub ($H(4)=7.0$, n=8941, $p=0.14$); used public transport or been in a taxi/minicab ($H(4)=1.1$, n=8941, $p=0.90$); left home to go to out to work (number of days) ($H(4)=4.3$, n=1904, $p=0.36$).

There were no differences in social mixing over time, stratified by risk of transmission ($H(4)=8.9$, $p=.06$; Figure 3).

Rates of always wearing a face covering increased over time in all settings (in shops for groceries / pharmacy ($\chi^2(4)=286.0$, n=7815, p<.001); in a restaurant, café or pub ($\chi^2(4)=90.9$, n=4497, p<.001); on public transport or in a taxi/minicab ($\chi^2(4)=50.8$, n=3310, p<.001); Figure 4).

Rates of testing increased over time (whole sample ($\chi^2=33.2$ (4), n=8780, $p<.001$; excluding people whose most recent test was a PCR test or who did not know what their most recent test type was ($\chi^2=32.4$ (4), n=7912, $p<.001$); Figure 5).

Omicron worry, perceived risk, and amount heard

Thirty nine percent to 42.7% of people reported being very or extremely worried about the Omicron variant (Table 2; Figure 1). More people (44.9% to 46.4%) perceived a major or significant risk of Omicron to themselves, with 56.7% to 61.4% of respondents perceiving a major or significant risk of Omicron to people in the UK. When applying a Bonferroni correction, there was no significant difference in Omicron worry or risk between Wave 63.5 and wave 64 data (worry: $F(1,3417)=4.74$, $p=.03$; perceived risk to self: $F(1,3371)=0.75$, $p=.39$; perceived risk to people in the UK: $F(1,3391)=7.67$, $p=.006$).

For peer review only - http://bmjopen.bmj.com/site/about/guidelines.xhtml

Table 2. Perceived worry about, and risk of, Omicron variant.

	Thinking about the Omicron variant, how worried are you about this specific variant of coronavirus?					Still thinking about the Omicron variant, to what extent do you think this specific variant of coronavirus poses a risk to you personally				Still thinking about the Omicron variant, to what extent do you think this specific variant of coronavirus poses a risk to people in the UK					How much, if anything, have you seen or heard about the new Omicron variant of coronavirus that was first detected in southern Africa?			
	Wave 63.5 [total n=1622]		Wave 64 [total n=1841]			Wave 63.5 [total n=1622]		Wave 64 [total n=1841]		Wave 63.5 [total n=1622]		Wave 64 [total n=1841]			Wave 63.5 [total n=1622]		Wave 64 [total n=1841]	
	% (95% CI)	n	% (95% CI)	n		% (95% CI)	n	% (95% CI)	n	% (95% CI)	n	% (95% CI)	n		% (95% CI)	n	% (95% CI)	n
Extremely worried	14.1 (12.5 to 15.9)	229	17.2 (15.4 to 18.9)	316	Major risk	20.6 (18.7 to 22.6)	334	23.2 (21.3 to 25.1)	427	25.5 (23.4 to 27.7)	414	29.0 (26.9 to 31.0)	533	I have seen or heard a lot	31.6 (29.3 to 33.9)	512	37.1 (34.9 to 39.3)	683
Very worried	24.4 (22.3 to 26.5)	395	25.0 (23.1 to 27.0)	461	Significant risk	22.7 (20.7 to 24.8)	369	22.4 (20.5 to 24.3)	413	29.5 (27.3 to 31.8)	478	31.7 (29.5 to 33.8)	583	I have seen or heard a fair amount	47.7 (45.3 to 50.2)	774	44.2 (41.9 to 46.5)	814
Somewhat worried	36.9 (34.6 to 39.3)	599	34.1 (31.9 to 36.3)	628	Moderate risk	27.9 (25.7 to 30.1)	452	26.6 (24.5 to 28.6)	489	27.2 (25.0 to 29.4)	441	23.8 (21.8 to 25.7)	438	I have seen or heard a little	18.7 (16.8 to 20.7)	303	16.3 (14.6 to 18.0)	300
Not very worried	16.5 (14.7 to 18.4)	267	14.4 (12.8 to 16.0)	265	Minor risk	20.3 (18.4 to 22.3)	329	21.0 (19.1 to 22.8)	386	12.2 (10.7 to 13.9)	198	11.2 (9.8 to 12.7)	207	I have not seen or heard anything	1.7 (1.1 to 2.4)	27	2.0 (1.3 to 2.6)	36
Not at all worried	6.7 (5.6 to 8.1)	109	8.1 (6.9 to 9.4)	150	No risk at all	4.9 (3.9 to 6.1)	80	5.1 (4.1 to 6.1)	94	2.7 (1.9 to 3.6)	43	3.2 (2.4 to 3.9)	58					
Don't know	1.4 (0.9 to 2.1)	23	1.1 (0.7 to 1.6)	21	Don't know	3.6 (2.7 to 4.6)	58	1.7 (1.1 to 2.3)	32	3.0 (2.2 to 3.9)	48	1.2 (0.7 to 1.7)	22	Don't know	0.4 (0.1 to 0.8)	6	0.4 (0.1 to 0.7)	8

For peer review only - http://bmjopen.bmj.com/site/about/guidelines.xhtml

Understanding of new rules

Understanding of the new rules introduced in response to Omicron was varied (Table 3). Understanding of rules requiring behaviour was good (around 80%+ correct, 90%+ correct on some rules). However, other items were answered incorrectly by most people, in the direction of believing that the rules were stricter than was the case. For some items (wearing a face covering in hospitality venues and all crowded and enclosed spaces), the percentage over-estimating the rules increased from Wave 63.5 to Wave 64. From 13 December 2021, people were asked to work from home if possible. This was the only rule that changed between survey waves, with high recognition in the latter wave.

For peer review only

Table 3. Endorsement of rules introduced in response to Omicron. Bold answers are correct.

The Government has issued new rules on how people should act to help prevent the spread of the Omicron variant of coronavirus. Please tell us, for the following options, if you think they are true or false?	Wave 63.5 [total n=1622]						Wave 64 [total n=1841]					
	True		False		Don't know		True		False		Don't know	
	% (95% CI)	n	% (95% CI)	n	% (95% CI)	n	% (95% CI)	n	% (95% CI)	n	% (95% CI)	n
You must wear a face covering in shops (unless you are exempt)*	**91.9 (90.5 to 93.2)**	**1490**	5.6 (4.5 to 6.7)	91	2.5 (1.8 to 3.3)	41	**90.3 (88.9 to 91.6)**	**1622**	5.4 (4.4 to 6.5)	100	4.3 (3.4 to 5.2)	79
You must wear a face covering on public transport (unless you are exempt)*	**91.1 (89.7 to 92.5)**	**1477**	6.2 (5.0 to 7.3)	100	2.8 (2.0 to 3.6)	45	**91.7 (90.5 to 93.0)**	**1689**	4.8 (3.8 to 5.8)	88	3.5 (2.6 to 4.3)	64
You must wear a face covering while moving around in restaurants, cafés and pubs (unless you are exempt)*	64.5 (62.2 to 66.8)	1046	**28.2 (26.0 to 30.4)**	**457**	7.3 (6.1 to 8.6)	119	71.2 (69.1 to 73.3)	1311	**19.5 (17.7 to 21.3)**	**359**	9.3 (8.0 to 10.6)	171
You must wear a face covering in all crowded and enclosed spaces where you come into contact with people you don't usually meet (unless you are exempt)†	77.9 (75.8 to 79.9)	1263	**15.2 (13.4 to 16.9)**	**246**	7.0 (5.7 to 8.2)	113	83.5 (81.8 to 85.2)	1538	**10.3 (8.9 to 11.7)**	**190**	6.1 (5.0 to 7.2)	113
All contacts of suspected Omicron cases must self-isolate, regardless of their vaccination status‡	**80.1 (78.1 to 82)**	**1299**	9.1 (7.7 to 10.5)	148	10.8 (9.3 to 12.3)	175	**76.9 (75.0 to 78.8)**	**1416**	12.7 (11.1 to 14.2)	233	10.4 (9.0 to 11.8)	192
You should stay at home as much as you can*	61.7 (59.3 to 64.1)	1001	**27.2 (25.0 to 29.4)**	**441**	11.1 (9.6 to 12.6)	180	69.5 (67.4 to 71.6)	1280	**20.4 (18.6 to 22.3)**	**376**	10.0 (8.7 to 11.4)	185
You should work from home if possible*	69.5 (67.3 to 71.8)	1128	**20.2 (18.3 to 22.2)**	**328**	10.2 (8.8 to 11.7)	166	**90.4 (89.0 to 91.7)**	**1664**	5.6 (4.6 to 6.7)	104	4.0 (3.1 to 4.9)	73
You cannot meet other people indoors, unless you live with them, or they are part of your support bubble*	38.1 (35.7 to 40.5)	618	**49.1 (46.6 to 51.5)**	**796**	12.8 (11.2 to 14.5)	208	36.1 (33.9 to 38.3)	665	**49.8 (47.5 to 52.1)**	**917**	14.1 (12.5 to 15.7)	259
International arrivals must take a PCR test by the end of the second day after arrival and self-isolate until they receive a negative result*	**84 (82.2 to 85.8)**	**1363**	7.6 (6.3 to 8.9)	123	8.4 (7.0 to 9.7)	136	**81.4 (79.6 to 83.2)**	**1499**	6.5 (5.3 to 7.6)	119	12.1 (10.6 to 13.6)	223
You must wear a face covering at the cinema or theatre¶	-	-	-	-	-	-	**85.2 (83.5 to 86.8)**	**1568**	7.4 (6.2 to 8.6)	137	7.4 (6.2 to 8.6)	136

* Previously a rule used to prevent the spread of SARS-CoV-2 in England.
† Not previously a rule used to prevent the spread of SARS-CoV-2 in England.
‡ Rule introduced to prevent the spread of the Omicron variant of concern.
¶ Previously a recommendation, but not a legal obligation, used to prevent the spread of SARS-CoV-2 in England.

For peer review only - http://bmjopen.bmj.com/site/about/guidelines.xhtml

For peer review only

Fewer than half of respondents agreed that the Government were putting the right measures in place to protect the UK public from Omicron, with around half agreeing that they had enough information about the symptoms of the Omicron variant and the effectiveness of vaccines against Omicron variant (Table 4). Most people agreed that they had enough information about what to do to prevent the spread of Omicron.

For peer review only

Table 4. Satisfaction with Government response to Omicron.

	The Government is putting the right measures in place to protect the UK public from the Omicron variant of coronavirus, % (n)		I have enough information from the Government and other public authorities on the symptoms of the Omicron variant of coronavirus, % (n)		I have enough information from the Government and other public authorities on how effective current vaccines are against the Omicron variant of coronavirus, % (n)		I have enough information from the Government and public authorities about what I can do to help prevent the spread of the Omicron variant of coronavirus, % (n)	
	Wave 63.5 [total n=1622]	Wave 64 [total n=1841]	Wave 63.5 [total n=1622]	Wave 64 [total n=1841]	Wave 63.5 [total n=1622]	Wave 64 [total n=1841]	Wave 63.5 [total n=1622]	Wave 64 [total n=1841]
Strongly agree	12.5 (203)	12.2 (224)	12.5 (203)	13.1 (242)	12.0 (195)	15.6 (287)	18.6 (301)	18.3 (336)
Agree	34.3 (557)	31.6 (581)	33.7 (546)	36.9 (680)	36.1 (585)	39.5 (728)	49.3 (799)	49.6 (913)
Neither agree nor disagree	23.0 (373)	22.8 (420)	23.9 (388)	21.6 (398)	22.7 (369)	21.0 (387)	17.8 (288)	18.2 (335)
Disagree	17.3 (281)	19.3 (356)	21.9 (355)	19.6 (360)	19.1 (310)	16.5 (303)	9.2 (149)	9.4 (173)
Disagree strongly	10.9 (176)	12.1 (222)	6.5 (105)	7.7 (142)	8.2 (133)	6.2 (115)	4.2 (68)	3.7 (69)
Don't know	2.0 (32)	2.1 (38)	1.5 (25)	1.0 (19)	1.8 (30)	1.1 (21)	1.0 (17)	0.8 (15)
Total strongly agree + agree, % (95% CI)	47.8 (45.3 to 50.3)	44.6 (42.4 to 46.9)	46.9 (44.5 to 49.4)	50.6 (48.3 to 52.9)	49.0 (46.5 to 51.5)	55.8 (53.5 to 58.1)	68.5 (66.3 to 70.8)	68.4 (66.3 to 70.5)
Total neither agree nor disagree + disagree + disagree strongly, % (95% CI)	52.2 (49.7 to 54.7)	55.4 (53.1 to 57.6)	53.1 (50.6 to 55.5)	49.4 (47.1 to 51.7)	51.0 (48.5 to 53.5)	44.2 (41.9 to 46.5)	31.5 (29.2 to 33.7)	31.6 (29.5 to 33.7)

For peer review only

Factors associated with engaging with protective behaviours

There were no significant associations between out-of-home activity and amount heard about Omicron, perceived worry (COVID-19 generally or Omicron specifically) or perceived risk (to oneself or people in UK, COVID-19 generally or Omicron specifically; Table 5). There were no associations with socio-demographic characteristics, with the exception of greater financial hardship being associated with going out shopping for items other than groceries/pharmacy (see supplementary materials Table 1).

For peer review only

For peer review only - http://bmjopen.bmj.com/site/about/guidelines.xhtml

Table 5. Associations between out-of-home activities and amount heard about Omicron, perceived worry, risk to self and risk to people in the UK. Bolding denotes significant findings (p<.002).

		Going out shopping (for groceries/pharmacy and other items)				Attending the workplace			
Attribute	Level	Wave 63.5 [a] IRR for going out shopping (95% CI)	*p*	Wave 64 [b] IRR for going out shopping (95% CI)	*p*	Wave 63.5 [c] IRR for attending the workplace (95% CI)	*p*	Wave 64 [d] aIRR for attending the workplace (95% CI)	*p*
Amount heard about Omicron variant †	I have not seen or heard anything (1) to I have seen or heard a lot (4)	1.05 (0.97 to 1.14)	0.23	1.07 (1.00 to 1.16)	0.05	1.02 (0.85 to 1.23)	0.82	1.03 (0.87 to 1.21)	0.77
Worry about COVID-19 †	Not at all worried (1) to extremely worried (5)	0.92 (0.87 to 0.97)	0.004	0.96 (0.91 to 1.01)	0.08	1.06 (0.93 to 1.21)	0.40	1.01 (0.89 to 1.14)	0.93
Worry about Omicron variant ‡	Not at all worried (1) to extremely worried (5)	0.94 (0.86 to 1.02)	0.15	0.93 (0.86 to 1.01)	0.09	0.93 (0.77 to 1.11)	0.40	0.95 (0.79 to 1.16)	0.63
Amount heard about Omicron variant †	I have not seen or heard anything (1) to I have seen or heard a lot (4)	1.05 (0.97 to 1.14)	0.26	1.08 (1.00 to 1.16)	0.04	1.04 (0.87 to 1.25)	0.65	1.02 (0.86 to 1.21)	0.79
Perceived risk of COVID-19 to self †	No risk at all (1) to major risk (5)	0.95 (0.90 to 1.00)	0.05	0.99 (0.94 to 1.04)	0.65	0.97 (0.86 to 1.08)	0.54	1.02 (0.92 to 1.14)	0.68
Perceived risk of Omicron variant to self ‡	No risk at all (1) to major risk (5)	1.00 (0.91 to 1.09)	0.95	1.02 (0.94 to 1.11)	0.59	1.08 (0.92 to 1.28)	0.35	1.04 (0.85 to 1.28)	0.70
Amount heard about Omicron variant †	I have not seen or heard anything (1) to I have seen or heard a lot (4)	1.04 (0.96 to 1.13)	0.30	1.07 (0.99 to 1.15)	0.08	1.04 (0.86 to 1.24)	0.70	1.02 (0.86 to 1.21)	0.81
Perceived risk of COVID-19 to people in UK †	No risk at all (1) to major risk (5)	0.96 (0.91 to 1.02)	0.24	0.98 (0.93 to 1.03)	0.45	1.01 (0.89 to 1.16)	0.83	0.98 (0.87 to 1.10)	0.74
Perceived risk of Omicron variant to people in UK ‡	No risk at all (1) to major risk (5)	0.92 (0.84 to 1.00)	0.05	1.10 (1.02 to 1.19)	0.02	1.11 (0.94 to 1.31)	0.24	1.01 (0.85 to 1.21)	0.88

† Adjusting for all other socio-demographic characteristics; amount heard about Omicron, and worry about COVID-19 / perceived risk of COVID-19 to self / perceived risk of COVID-19 to people in the UK.

‡ Adjusting for all other socio-demographic characteristics; amount heard about Omicron, and worry about COVID-19 / perceived risk of COVID-19 to self / perceived risk of COVID-19 to people in the UK; and Omicron-specific worry / perceived risk to self / perceived to people in the UK.

a) 1622 people were eligible for inclusion in analyses investigating going out shopping analyses. There were different amounts of missing data depending on variables included in the models, so n ranged between 1440 and 1491 for different models.

b) 1841 people were eligible for inclusion in analyses investigating going out shopping analyses. There were different amounts of missing data depending on variables included in the models, so n ranged between 1671 and 1713 for different models.

c) 374 people were eligible for inclusion in analyses investigating non-essential workplace attendance (sample limited to people who reported they could work entirely from home). Due to missing data, n included in analyses ranged between 349 and 354.

d) 410 people were eligible for inclusion in analyses investigating non-essential workplace attendance (sample limited to people who reported they could work entirely from home). Due to missing data, n included in analyses ranged between 379 and 389.

For peer review only - http://bmjopen.bmj.com/site/about/guidelines.xhtml

For peer review only

Engaging in highest risk social mixing and always wearing a face covering in hospitality venues were associated with worry about, and perceived risk of, COVID-19 (Table 6). Always wearing a face covering in shops was independently associated with having heard more about Omicron. Associations between behaviour and Omicron-specific worry and perceived risk often did not reach the statistical significance level required after a Bonferroni correction but showed some relationship with behaviour. Always wearing a face covering was positively associated with having been vaccinated (see supplementary materials Table 2).

For peer review only

For peer review only - http://bmjopen.bmj.com/site/about/guidelines.xhtml

Table 6. Associations between highest risk social mixing and wearing a face covering and amount heard about Omicron, perceived worry, risk to self and risk to people in the UK. Bolding denotes significant findings (p<.002).

		Highest risk social mixing				Always wearing a face covering in shops				Always wearing a face covering in hospitality venues			
Attribute	Level	Wave 63.5 [a] aOR for engaging in highest risk social mixing (95% CI)	*p*	Wave 64 [b] aOR for engaging in highest risk social mixing (95% CI)		Wave 63.5 [c] aOR for wearing a face covering in shops (95% CI)	*p*	Wave 64 [d] aOR for wearing a face covering in shops (95% CI)	*p*	Wave 63.5 [e] aOR for wearing a face covering in hospitality venues (95% CI)	*p*	Wave 64 [f] aOR for wearing a face covering in hospitality venues (95% CI)	*p*
Amount heard about Omicron variant †	I have not seen or heard anything (1) to I have seen or heard a lot (4)	1.03 (0.88 to 1.20)	0.72	1.08 (0.94 to 1.24)	0.30	**1.46 (1.19 to 1.79)**	**<0.001**	1.32 (1.09 to 1.59)	0.004	1.12 (0.89 to 1.40)	0.34	1.25 (1.02 to 1.53)	0.03
Worry about COVID-19 †	Not at all worried (1) to extremely worried (5)	**0.79 (0.71 to 0.88)**	**<0.001**	**0.73 (0.66 to 0.80)**	**<0.001**	**1.43 (1.24 to 1.65)**	**<0.001**	1.44 (1.26 to 1.64)	**<0.001**	**1.55 (1.31 to 1.84)**	**<0.001**	**1.34 (1.17 to 1.55)**	**<0.001**
Worry about Omicron variant ‡	Not at all worried (1) to extremely worried (5)	**0.76 (0.65 to 0.89)**	**0.001**	0.93 (0.79 to 1.09)	0.35	1.24 (1.00 to 1.53)	0.05	1.35 (1.09 to 1.68)	0.006	1.18 (0.95 to 1.47)	0.13	1.26 (1.02 to 1.56)	0.03
Amount heard about Omicron variant †	I have not seen or heard anything (1) to I have seen or heard a lot (4)	0.99 (0.85 to 1.15)	0.88	1.03 (0.89 to 1.18)	0.69	**1.50 (1.22 to 1.84)**	**<0.001**	1.33 (1.10 to 1.61)	0.003	1.17 (0.93 to 1.46)	0.17	1.32 (1.08 to 1.62)	0.007
Perceived risk of COVID-19 to self †	No risk at all (1) to major risk (5)	**0.85 (0.76 to 0.94)**	**0.001**	**0.78 (0.71 to 0.86)**	**<0.001**	**1.39 (1.21 to 1.60)**	**<0.001**	**1.25 (1.10 to 1.41)**	**<0.001**	**1.35 (1.16 to 1.57)**	**<0.001**	**1.24 (1.09 to 1.41)**	**0.001**
Perceived risk of Omicron variant to self ‡	No risk at all (1) to major risk (5)	0.85 (0.72 to 0.99)	0.04	0.91 (0.78 to 1.05)	0.20	1.20 (0.97 to 1.48)	0.09	1.23 (1.00 to 1.50)	0.05	1.26 (0.99 to 1.60)	0.06	1.03 (0.83 to 1.27)	0.81
Amount heard about Omicron variant †	I have not seen or heard anything (1) to I have seen or heard a lot (4)	1.00 (0.86 to 1.17)	0.98	1.03 (0.90 to 1.19)	0.65	**1.53 (1.25 to 1.87)**	**<0.001**	1.35 (1.12 to 1.63)	0.002	1.16 (0.93 to 1.45)	0.19	1.32 (1.08 to 1.63)	0.007

For peer review only - http://bmjopen.bmj.com/site/about/guidelines.xhtml

For peer review only

Perceived risk of COVID-19 to people in UK †	No risk at all (1) to major risk (5)	**0.82 (0.73 to 0.91)**	**<0.001**	**0.83 (0.76 to 0.92)**	**<0.001**	**1.28 (1.11 to 1.49)**	**0.001**	**1.41 (1.23 to 1.61)**	**<0.001**	**1.41 (1.20 to 1.67)**	**<0.001**	**1.28 (1.11 to 1.48)**	**0.001**
Perceived risk of Omicron variant to people in UK ‡	No risk at all (1) to major risk (5)	0.93 (0.80 to 1.09)	0.37	0.91 (0.78 to 1.05)	0.20	1.33 (1.09 to 1.63)	0.006	1.37 (1.12 to 1.68)	0.002	1.42 (1.14 to 1.78)	0.002	1.20 (0.98 to 1.46)	0.08

† Adjusting for all other socio-demographic characteristics; amount heard about Omicron, and worry about COVID-19 / perceived risk of COVID-19 to self / perceived risk of COVID-19 to people in the UK.

‡ Adjusting for all other socio-demographic characteristics; amount heard about Omicron, and worry about COVID-19 / perceived risk of COVID-19 to self / perceived risk of COVID-19 to people in the UK; and Omicron-specific worry / perceived risk to self / perceived to people in the UK.

a) 1622 people were eligible for inclusion in highest risk social mixing analyses. There were different amounts of missing data depending on variables included in the models, so n ranged between 1439 and 1446 for different models.

b) 1841 people were eligible for inclusion in highest risk social mixing analyses. There were different amounts of missing data depending on variables included in the models, so n ranged between 1668 and 1689 for different models.

c) 1404 people were eligible for inclusion in wearing a face covering in shops analyses (sample limited to people who reported having been out shopping in the last week). There were different amounts of missing data depending on variables included in the models, so n ranged between 1247 and 1266 for different models.

d) 1600 people were eligible for inclusion in wearing a face covering in shops analyses (sample limited to people who reported having been out shopping in the last week). There were different amounts of missing data depending on variables included in the models, so n ranged between 1454 and 1475 for different models.

e) 789 people were eligible for inclusion in wearing a face covering in hospitality venues analyses (sample limited to people who reported having been out to hospitality venues in the last week). There were different amounts of missing data depending on variables included in the models, so n ranged between 700 and 713 for different models.

f) 894 people were eligible for inclusion in wearing a face covering in hospitality venues analyses (sample limited to people who reported having been out to hospitality venues in the last week). There were different amounts of missing data depending on variables included in the models, so n ranged between 817 and 829 for different models.

For peer review only

For peer review only - http://bmjopen.bmj.com/site/about/guidelines.xhtml

Discussion

These findings suggest that initial reporting of the emergence of Omicron had little impact on public perceptions. There were small increases in worry about, and perceived risk of, COVID-19 in the days after the emergence of Omicron was reported. While over one third of participants reported being very or extremely worried about Omicron, and over half of respondents perceived a major or significant risk of Omicron to people in the UK, these figures were very close to the rates observed for concerns about 'coronavirus' in general.

Engagement with wearing a face covering and testing increased between 1 November and 16 December 2021. Approximately 80% of the sample reported "always" wearing a face covering while in shops. This rate is similar to the percentage who reported "frequently" or "very frequently" wearing a face covering outside the home during the second lockdown in England (November 2020).(25) Rates of wearing a face covering increased even in hospitality settings, where rules were not changed, possibly reflecting the misunderstanding of the extent of official guidance that this study observed. A survey by the English Office for National Statistics also showed an increase in wearing a face covering in data collected 1 to 12 December 2021.(26) Increases in uptake of testing may reflect a higher prevalence of symptoms in the population during this period.(27) While there have been media reports of behaviour change in response to Omicron (for example, restaurant industry figures reporting a fall in eating out early on),(28) our results show that there were few changes in out-of-home activity up to 16 December 2021. This is in line with other polling carried out on 14 to 15 December 2021.(29) Despite Omicron being a key story in the media, it appears that early behavioural responses to it were largely restricted to changes that were required by legislation, rather than more spontaneous changes among the public.

Despite over one-third of people thinking that indoor mixing with other households was not allowed, there were no changes in patterns of social mixing. Our question on knowledge of the rules may be insensitive to degrees of certainty or may be demonstrating a social desirability effect. Social mixing may normally increase in the run-up to Christmas, so we cannot tell whether a flat statistic actually represents a reduction compared to the likely pattern for the time of year. Nonetheless, in contrast to the early stages of the COVID-19 pandemic, we have not yet observed a substantial "spill-over" effect involving non-recommended behaviours following the emergence of the Omicron variant.

Previous research has suggested that a constant stream of changes to guidance over the course of the pandemic left many people confused and disengaged.(30, 31) Understanding of the new rules in response to Omicron was mixed. In general, people greatly over-estimated the stringency of the rules. This had the potential to be positive in terms of reducing transmission, but also the potential to have had a negative impact in terms of wellbeing,(32) economic activity,(28) and social tension.(33) Additional rules were introduced on 13 December 2021 (England's "Plan B", working from home where possible, face coverings becoming compulsory in most public indoor venues apart from hospitality, introduction of vaccine passports in some settings).(11) Recognition of the rule regarding working from home increased in data collected 13 to 16 December, but there was no evidence for a corresponding change in behaviour. This is likely because we measured behaviour in the previous week, before the rule was introduced. Furthermore, there was no legal underpinning to this rule in England, unlike during the third UK lockdown.(34)

We investigated associations between engaging in protective behaviours that had and had not been legislated for, and worry and perceived risk. Engaging in highest risk social mixing and always wearing a face covering in hospitality venues and while shopping were associated independently with worry about, and perceived risk of, COVID-19in general. There were no associations for out-of-

For peer review only - http://bmjopen.bmj.com/site/about/guidelines.xhtml

home activity (shopping and non-essential workplace attendance). Out-of-home activities may be perceived as being necessary (e.g. shopping for provisions, or attending the workplace at your employer's request). Results suggest that those behaviours that are perceived as being within one's control, such as wearing a face covering and engaging in risky social mixing, may be more affected by psychological factors.(35) Similar patterns of results and strengths of associations were seen for associations between behaviours and perceived risk to oneself and others in the UK. This is a slight difference to some previous research, which showed stronger associations between behaviour and perceived risk to others.(36, 37) Of behaviours investigated, only wearing a face covering while shopping was a legislated behaviour. Wearing a face covering was also initially associated with having heard more about Omicron (wave 63.5). Data are cross-sectional and we cannot tell the direction of causation. It may be the case that people who wear face coverings are more likely to pay attention to news about COVID-19.

To the best of our knowledge, this is the first study investigating the influence of the Omicron variant on public worry, perceived risk and behaviour. This rapid response was facilitated by having regular data collection measuring public behaviour and attitudes. Limitations of the study include the use of self-reported data. We have previously noted that self-reported face covering wearing is likely to over-estimate observed rates, although self-reports of "always" wearing a face covering in a particular location appear more robust.(6)

Participants in our study were slightly more likely to be female, white, and highly educated than the general population.(23, 24) Whether the behaviour and attitudes of people who sign up to take part in surveys is representative of the behaviour and attitudes of the general population is unknown. Official statistics on uptake of the COVID-19 vaccine report percentages of the population aged 12 years and over.(38) Our sample comprised people aged 16 years and over and so are not directly comparable. Participants were asked to report on their behaviour in the last week. For wave 63, 63.5 and 64 data, this overlapped the period before and after rules (in response to the Omicron variant, and England's "Plan B") came into force. We did not investigate factors associated with all potential out-of-home activities, nor uptake of testing, as this would have been too many outcomes. We focused our analyses on activities where the chance of coming into close contact with people from other households was greatest, and where legislation had recently changed. We investigated wearing a face covering only in people who reported having been out shopping or to hospitality venues in the past week. Workplace attendance was investigated only in those who reported being able to fully work from home. This limited our sample size and our ability to detect small effects. Data are cross-sectional and we are unable to determine direction of associations. One complicating factor for our analyses a national discussion around "partygate," a news story that broke in November 2021 and was highly publicised in the following weeks, reporting on multiple occasions when Government employees (including the Prime Minister) had attended gatherings that breached COVID-19 regulations (39, 40). This occurred at around the same time as the emergence of Omicron. A debate has developed over what, if any, effects the reporting about these social events had on public adherence.(41) We do not know if perceptions or behaviours might have been different, had reporting of these events not occurred at this time.

The Omicron variant emerged almost two years after the start of the COVID-19 outbreak. Despite substantial uncertainty about the impact of the resulting wave of infections, our data indicate that the emergence of the Omicron variant only slightly influenced worry about and perceived risk of COVID-19, suggesting a degree of habituation among the public to new announcements about the pandemic. Despite this, wearing a face covering, the main legislated change in response to Omicron, and uptake of testing increased between 1 November and 16 December 2021. These results suggest

For peer review only - http://bmjopen.bmj.com/site/about/guidelines.xhtml

that specific behaviour changes continued to occur in response to changes in rules. Amount heard about Omicron was associated with always wearing a face covering, suggesting that communications emphasising protective behaviours may also increase engagement for behaviours that are required by law.

For peer review only

For peer review only - http://bmjopen.bmj.com/site/about/guidelines.xhtml

Funding statement

This work was funded by the National Institute for Health Research (NIHR) Health Services and Delivery Research programme (NIHR project reference number (11/46/21)). Surveys were commissioned and funded by Department of Health and Social Care (DHSC), with the authors providing advice on the question design and selection. LS, RA and GJR are supported by the National Institute for Health Research Health Protection Research Unit (NIHR HPRU) in Emergency Preparedness and Response, a partnership between the UK Health Security Agency, King's College London and the University of East Anglia. RA is also supported by the NIHR HPRU in Behavioural Science and Evaluation, a partnership between the UK Health Security Agency and the University of Bristol. HWWP has received funding from Public Health England and NHS England. NTF is part funded by a grant from the UK Ministry of Defence. The views expressed are those of the authors and not necessarily those of the NIHR, UK Health Security Agency, the Department of Health and Social Care or the Ministry of Defence. The Department of Health and Social Care funded data collection (no grant number).

Competing interests statement

All authors have completed the ICMJE uniform disclosure form at www.icmje.org/coi_disclosure.pdf and declare: all authors had financial support from NIHR for the submitted work; RA is an employee of the UK Health Security Agency; HWWP received additional salary support from Public Health England and NHS England; HWWP receives consultancy fees to his employer from Ipsos MORI and has a PhD student who works at and has fees paid by Astra Zeneca; no other financial relationships with any organisations that might have an interest in the submitted work in the previous three years; no other relationships or activities that could appear to have influenced the submitted work. NTF is a participant of an independent group advising NHS Digital on the release of patient data. At the time of writing GJR is acting as an expert witness in an unrelated case involving Bayer PLC, supported by LS. All authors were participants of the UK's Scientific Advisory Group for Emergencies or its subgroups.

Author contribution statement

All authors conceptualised the study and contributed to survey materials. LS completed analyses with guidance from HWWP and GJR. LS and GJR wrote the first draft of the manuscript. HWWP, RA, NTF, and SM contributed to subsequent drafts of the manuscript. LS, HWWP, RA, NTF, SM and GJR approved the final manuscript. GJR is guarantor. The corresponding author attests that all listed authors meet authorship criteria and that no others meeting the criteria have been omitted.

Data sharing statement

No additional data are available from the authors.

Ethics

This work was conducted as a service evaluation of the Department of Health and Social Care's public communications campaign. Following advice from King's College London Research Ethics Committee, it was exempt from requiring ethical approval.

Participants of online research panels have consented to being contacted to take part in online surveys. Following industry standards, consent was implied by participants' completion of the survey.

For peer review only

For peer review only - http://bmjopen.bmj.com/site/about/guidelines.xhtml

Figure legends

Figure 1. Perceived worry about, and risk of, COVID-19 between 1st November and 16th December 2021.

Figure 2. Out-of-home activity, between 1st November and 16th December 2021.

Figure 3. Risky social mixing, between 1st November and 16th December 2021.

Figure 4. Always wearing a face covering, between 1st November and 16th December 2021.

Figure 5. Uptake of testing, between 1st November and 16th December 2021. The dashed line shows the seven-day average for new cases in England.

For peer review only

For peer review only - http://bmjopen.bmj.com/site/about/guidelines.xhtml

References

1. World Health Organization. Classification of Omicron (B.1.1.529): SARS-CoV-2 Variant of Concern. 2021. Available from: https://www.who.int/news/item/26-11-2021-classification-of-omicron-(b.1.1.529)-sars-cov-2-variant-of-concern.

2. Merrick J. Covid 'super variant' with 32 mutations found with cases in South Africa, Botswana and Hong Kong. iNews. 24 November 2021. Available from: https://inews.co.uk/news/politics/covid-super-variant-mutations-cases-found-south-africa-botswana-hong-kong-1316864.

3. Sky News. COVID-19: 'Really awful' new coronavirus variant B.1.1.529 identified in Botswana. 25 November 2021. Available from: https://news.sky.com/story/covid-19-new-coronavirus-variant-b-1-1-529-identified-in-botswana-as-scientists-play-down-concerns-12478164.

4. Smith LE, Potts HWW, Amlôt R, Fear N, Michie S, Rubin GJ. Worry and behaviour at the start of the COVID-19 outbreak: results from three UK surveys. Prev Med Rep. 2022;25(101686).

5. Prime Minister confirms move to Step 4 [press release]. 12 July 2021. Available from: https://www.gov.uk/government/news/prime-minister-confirms-move-to-step-4.

6. Davies R, Martin AF, Smith LE, Mowbray F, Woodland L, Amlot R, et al. The impact of "freedom day" on COVID-19 health protective behaviour in England: An observational study of hand hygiene, face covering use and physical distancing in public spaces pre and post the relaxing of restrictions. Open Science Framework. 2021. DOI [pre-print]: 10.17605/OSF.IO/CK2U4

7. First UK cases of Omicron variant identified [press release]. 2021. Available from: https://www.gov.uk/government/news/first-uk-cases-of-omicron-variant-identified.

8. Prime Minister sets out new measures as Omicron variant identified in UK: 27 November 2021 [press release]. 27 November 2021. Available from: https://www.gov.uk/government/news/prime-minister-sets-out-new-measures-as-omicron-variant-identified-in-uk-27-november-2021.

9. Measures against Omicron variant come into effect: 30 November 2021 [press release]. 30 November 2021. Available from: https://www.gov.uk/government/news/measures-against-omicron-variant-come-into-effect-30-november-2021.

10. Northern Ireland Department of Health. 'Lateral Flow before you go'. 2021. Available from: https://www.health-ni.gov.uk/news/lateral-flow-you-go.

11. Prime Minister's Office 10 Downing Street. Prime Minister confirms move to Plan B in England. 2021. Available from: https://www.gov.uk/government/news/prime-minister-confirms-move-to-plan-b-in-england.

12. Jessica Elgot, Nicola Davis. No new Covid restrictions in England before new year, Boris Johnson says. The Guardian. 27 December 2021. Available from: https://www.theguardian.com/world/2021/dec/27/boris-johnson-will-impose-no-further-covid-restrictions-before-new-year.

13. Reicher S, Drury J. Pandemic fatigue? How adherence to covid-19 regulations has been misrepresented and why it matters. BMJ Opinion. 2021.

For peer review only

14. Smith LE, Potts HWW, Amlôt R, Fear N, Michie S, Rubin GJ. How have patterns of social mixing changed during the pandemic? A series of cross-sectional nationally representative surveys. Open Science Framework. 2021. DOI [pre-print]: 10.17605/OSF.IO/DQA7Z.

15. Rubin GJ, Potts HWW, Michie S. The impact of communications about swine flu (influenza A H1N1v) on public responses to the outbreak: results from 36 national telephone surveys in the UK. Health Technol Asses. 2010;14(34):183-266.

16. Rubin GJ, Amlot R, Page L, Wessely S. Public perceptions, anxiety, and behaviour change in relation to the swine flu outbreak: cross sectional telephone survey. BMJ. 2009;339:b2651.

17. Dryhurst S, Schneider CR, Kerr J, Freeman ALJ, Recchia G, van der Bles AM, et al. Risk perceptions of COVID-19 around the world. J Risk Res. 2020:1-13.

18. Slovic P, Fischhoff B, Lichtenstein S. Facts and fears, Understanding perceived risk. In: Schwing R, Albers Jr. WA, editors. How safe is safe enough? New York: Plenum; 1980.

19. Smith LE, Potts HWW, Amlot R, Fear NT, Michie S, Rubin GJ. Adherence to the test, trace, and isolate system in the UK: results from 37 nationally representative surveys. BMJ. 2021;372:n608.

20. NHS. Who is at high risk from coronavirus (COVID-19). 2021. Available from: https://www.nhs.uk/conditions/coronavirus-covid-19/people-at-higher-risk/who-is-at-high-risk-from-coronavirus/.

21. Ministry of Housing Communities and Local Government. The English Indices of Deprivation 2019 (IoD2019). 2019. Available from: https://assets.publishing.service.gov.uk/government/uploads/system/uploads/attachment_data/file/835115/IoD2019_Statistical_Release.pdf.

22. Rubin GJ, Bakhshi S, Amlôt R, Fear N, Potts HWW, Michie S. The design of a survey questionnaire to measure perceptions and behaviour during an influenza pandemic: the Flu TElephone Survey Template (FluTEST). Health Services and Delivery Research. 2014;2(41).

23. GOV.UK. Regional ethnic diversity. 2020. Available from: https://www.ethnicity-facts-figures.service.gov.uk/uk-population-by-ethnicity/national-and-regional-populations/regional-ethnic-diversity/latest#areas-of-england-and-wales-by-ethnicity.

24. Office for National Statistics. 2011 Census: Key Statistics and Quick Statistics for Local Authorities in the United Kingdom. 2013. Available from: https://www.ons.gov.uk/employmentandlabourmarket/peopleinwork/employmentandemployeetypes/bulletins/keystatisticsandquickstatisticsforlocalauthoritiesintheunitedkingdom/2013-12-04.

25. Smith LE, Potts HWW, Amlôt R, Fear N, Michie S, Rubin GJ. Engagement with protective behaviours in the UK during the COVID-19 pandemic: a series of cross-sectional surveys (the COVID-19 rapid survey of adherence to interventions and responses [CORSAIR] study). BMC Public Health. 2022;22(1):475.

26. Office for National Statistics. Coronavirus and the social impacts on Great Britain: 17 December 2021. 2021. Available from: https://www.ons.gov.uk/peoplepopulationandcommunity/healthandsocialcare/healthandwellbeing/bulletins/coronavirusandthesocialimpactsongreatbritain/17december2021.

27. UK Health Security Agency. Weekly national Influenza and COVID-19 surveillance report Week 50 report (up to week 49 data) 16 December 2021. 2021. Available from:

For peer review only - http://bmjopen.bmj.com/site/about/guidelines.xhtml

https://assets.publishing.service.gov.uk/government/uploads/system/uploads/attachment_data/file/1041447/Weekly_Flu_and_COVID-19_report_w50.pdf.

28. Richard Partington. Dining out in UK at lowest level since May amid Omicron fears. The Guardian. 2 December 2021. Available from: https://www.theguardian.com/business/2021/dec/02/dining-out-in-uk-at-lowest-level-since-may-amid-omicron-fears.

29. Connor Ibbetson. Most Britons are carrying on as normal despite Omicron. 2021. Available from: https://yougov.co.uk/topics/lifestyle/articles-reports/2021/12/17/most-britons-are-carrying-normal-despite-omicron.

30. Williams SN, Armitage CJ, Tampe T, Dienes K. Public perceptions and experiences of social distancing and social isolation during the COVID-19 pandemic: a UK-based focus group study. BMJ Open. 2020;10(7):e039334.

31. Williams SN, Dienes K. 'Variant fatigue'? Public attitudes to COVID-19 18 months into the pandemic: A qualitative study. PsyArXiv. 2021. DOI [pre-print]: 10.31234/osf.io/vam4t.

32. Smith LE, Amlôt R, Fear NT, Michie S, Rubin GJ, Potts HWW. Psychological wellbeing in the English population during the COVID-19 pandemic: a series of cross-sectional surveys. OSF. 2021. DOI [pre-print]: 10.17605/OSF.IO/U34F6.

33. Smith LE, Duffy B, Moxham-Hall V, Strang L, Wessely S, Rubin GJ. Anger and confrontation during the COVID-19 pandemic: a national cross-sectional survey in the UK. J R Soc Med. 2021;114(2):77-90.

34. Cabinet Office. COVID-19 Response - Spring 2021 (Summary). 2021. Available from: https://www.gov.uk/government/publications/covid-19-response-spring-2021/covid-19-response-spring-2021-summary.

35. Smith LE, Potts HWW, Amlot R, Fear NT, Michie S, Rubin GJ. Tiered restrictions for COVID-19 in England: knowledge, motivation and self-reported behaviour. Public Health. 2022;204:33-9.

36. Smith LE, Potts HWW, Amlot R, Fear NT, Michie S, Rubin GJ. Intention to adhere to test, trace, and isolate during the COVID-19 pandemic (the COVID-19 Rapid Survey of Adherence to Interventions and Responses study). Br J Health Psychol. 2021.

37. Sherman SM, Smith LE, Sim J, Amlot R, Cutts M, Dasch H, et al. COVID-19 vaccination intention in the UK: results from the COVID-19 vaccination acceptability study (CoVAccS), a nationally representative cross-sectional survey. Hum Vaccin Immunother. 2021;17(6):1612-21.

38. GOV.UK. Vaccinations in England. 2022. Available from: https://coronavirus.data.gov.uk/details/vaccinations?areaType=nation&areaName=England.

39. Cabinet Office. Investigation into alleged gatherings on government premises during covid restrictions - update. 31 January 2022. Available from: https://assets.publishing.service.gov.uk/government/uploads/system/uploads/attachment_data/file/1051374/Investigation_into_alleged_gatherings_on_government_premises_during_Covid_restrictions_-_Update.pdf.

40. Institute for Government. Sue Gray investigation. 2022. Available from: https://www.instituteforgovernment.org.uk/explainers/sue-gray-investigation.

For peer review only - http://bmjopen.bmj.com/site/about/guidelines.xhtml

41. Reicher S. It is wrong to claim that the public won't follow covid rules just because the government don't. BMJ Opinion. 2021;375(n3150).

For peer review only

For peer review only - http://bmjopen.bmj.com/site/about/guidelines.xhtml

Figure 1. Perceived worry about, and risk of, COVID-19 between 1st November and 16th December 2021.

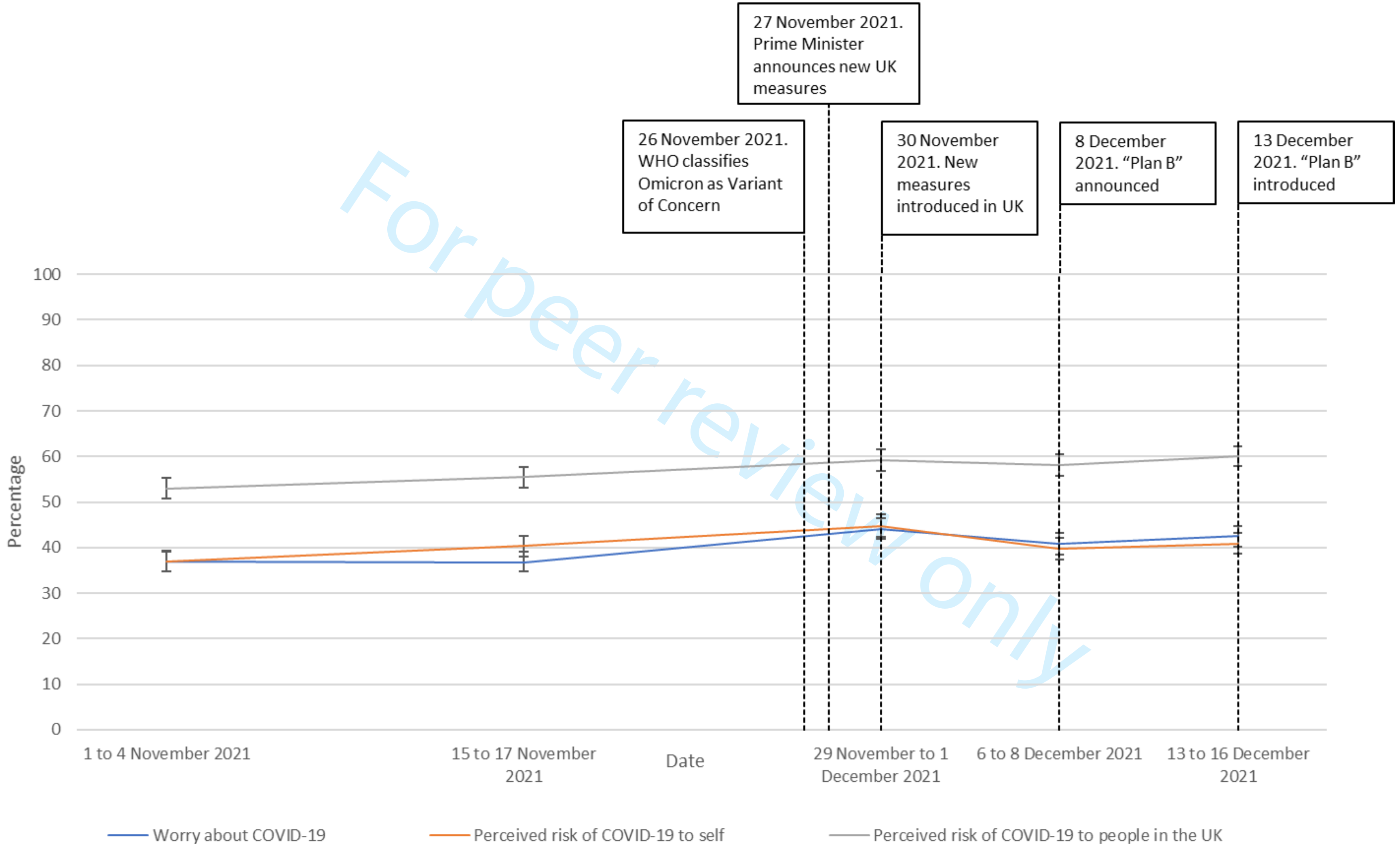

For peer review only

Figure 2. Out-of-home activity, between 1st November and 16th December 2021.

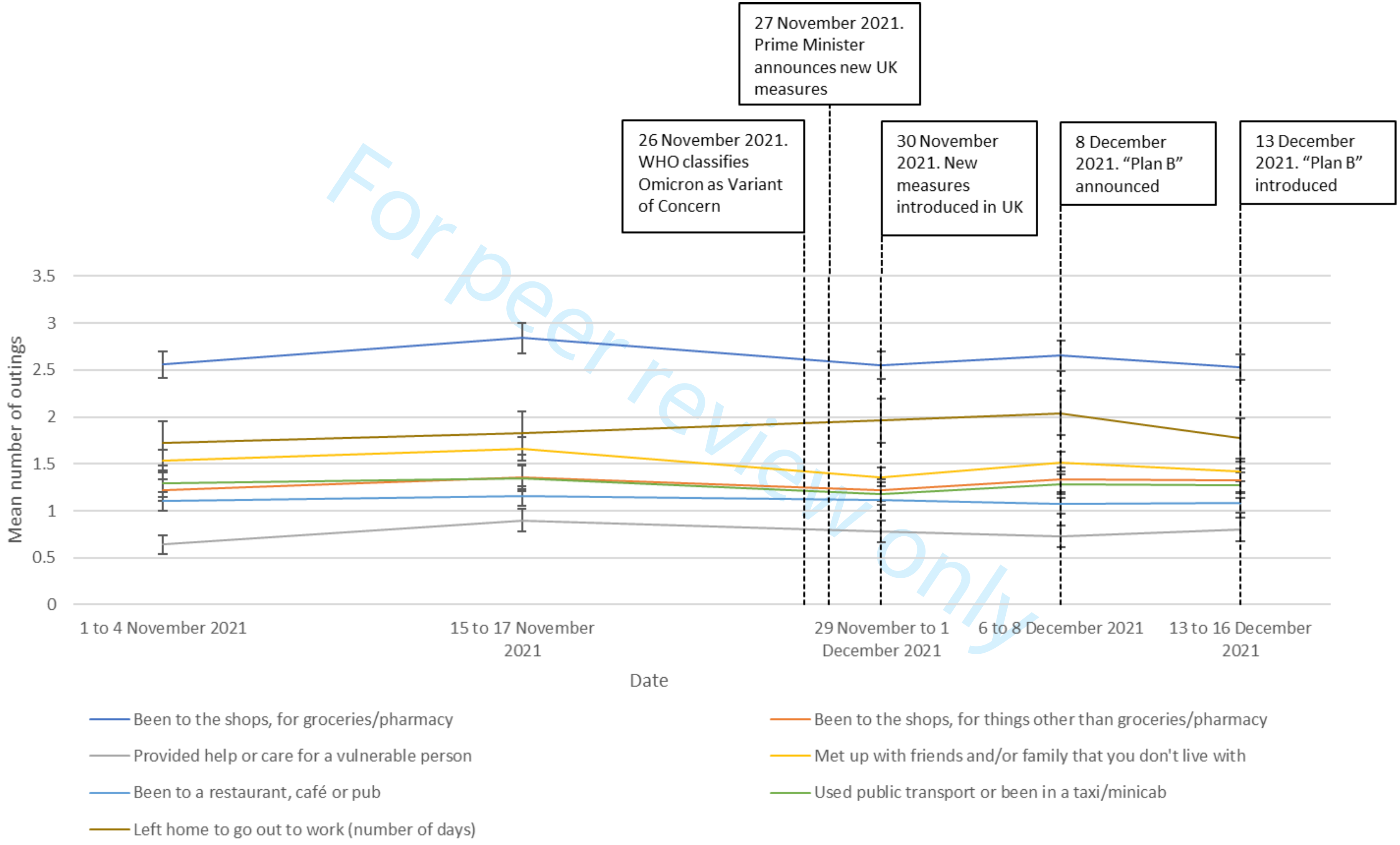

Figure 3. Risky social mixing, between 1st November and 16th December 2021.

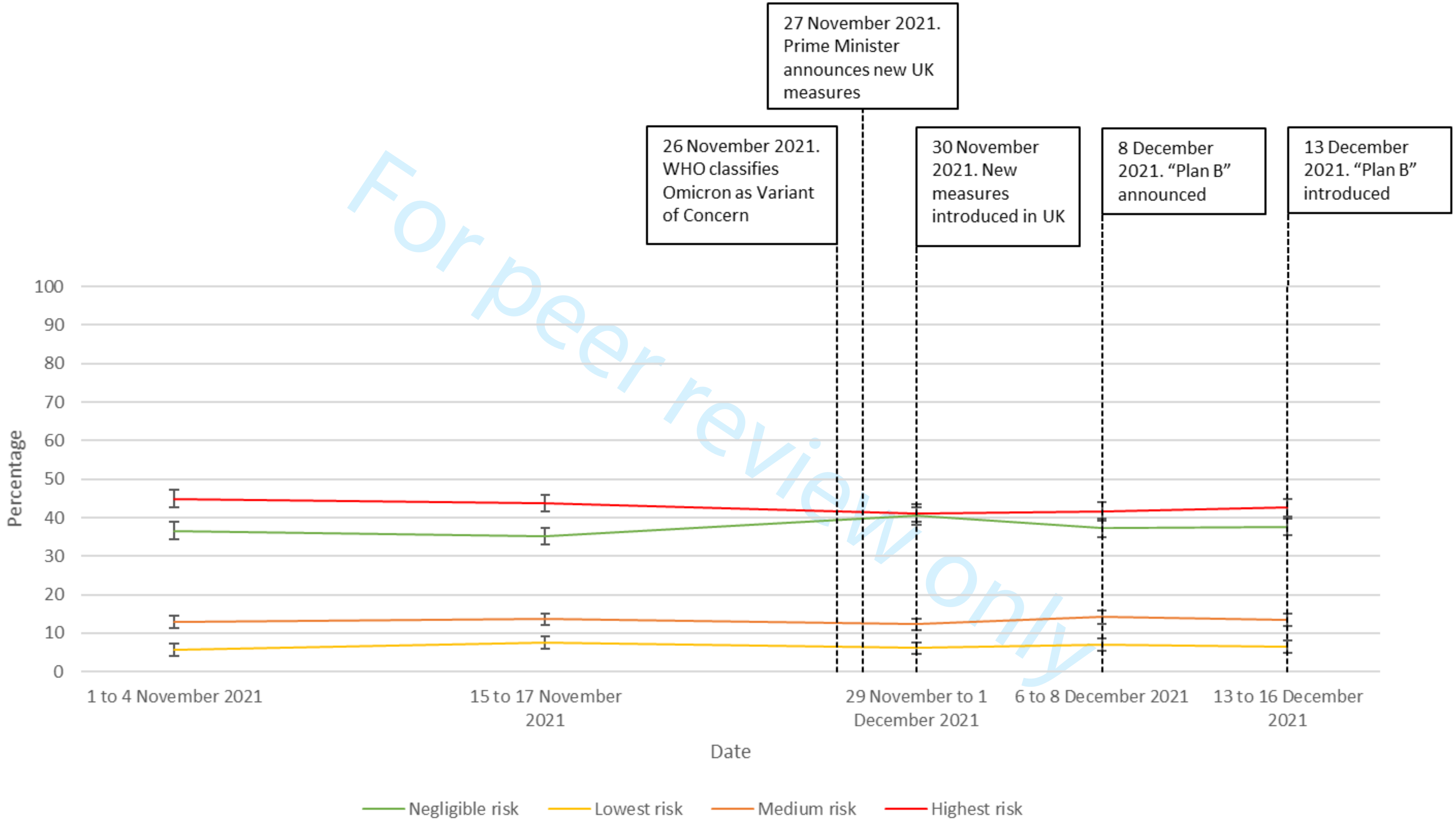

For peer review only

Figure 4. Always wearing a face covering, between 1st November and 16th December 2021.

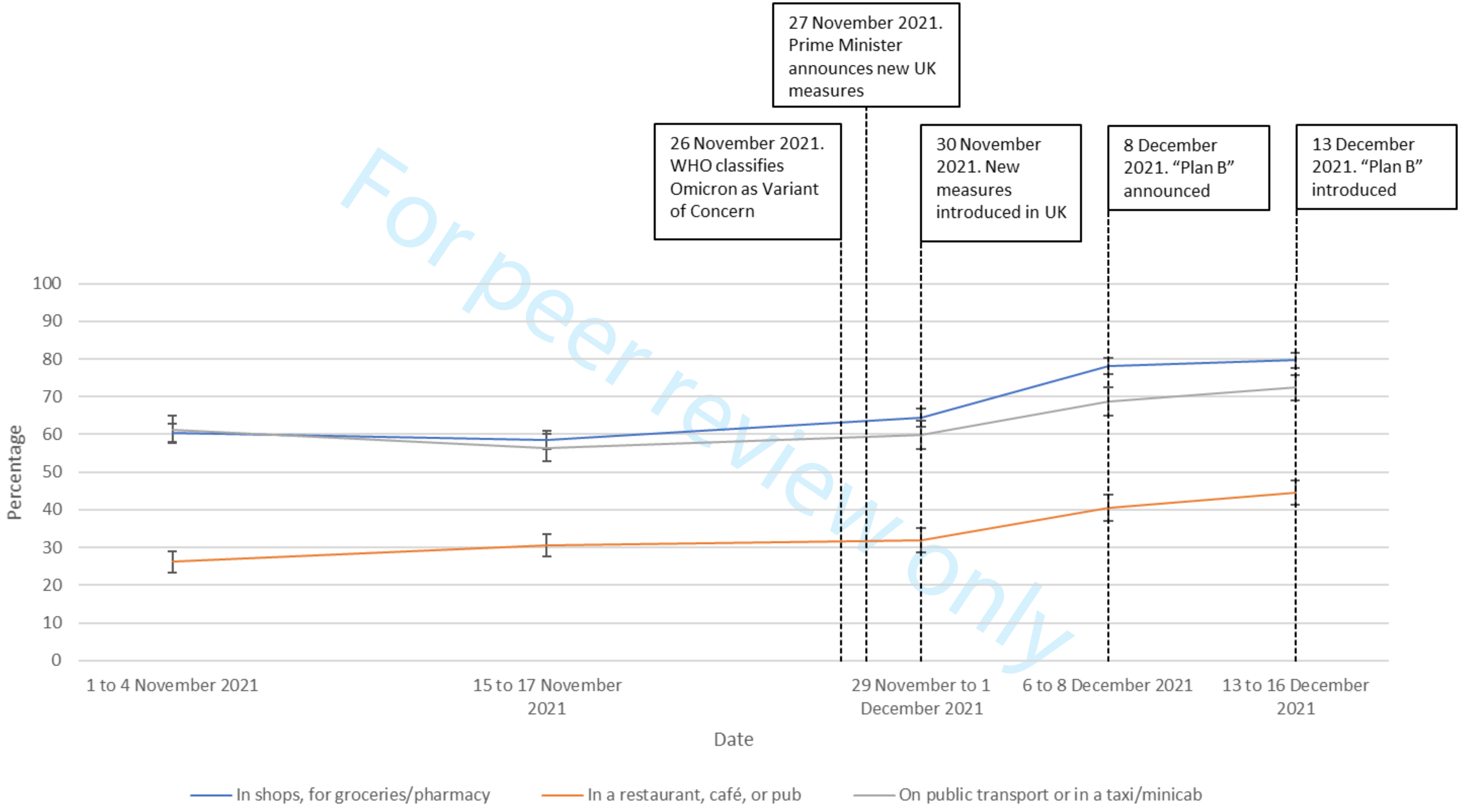

Figure 5. Uptake of testing, between 1st November and 16th December 2021. The dashed line shows the seven-day average for new cases in England.

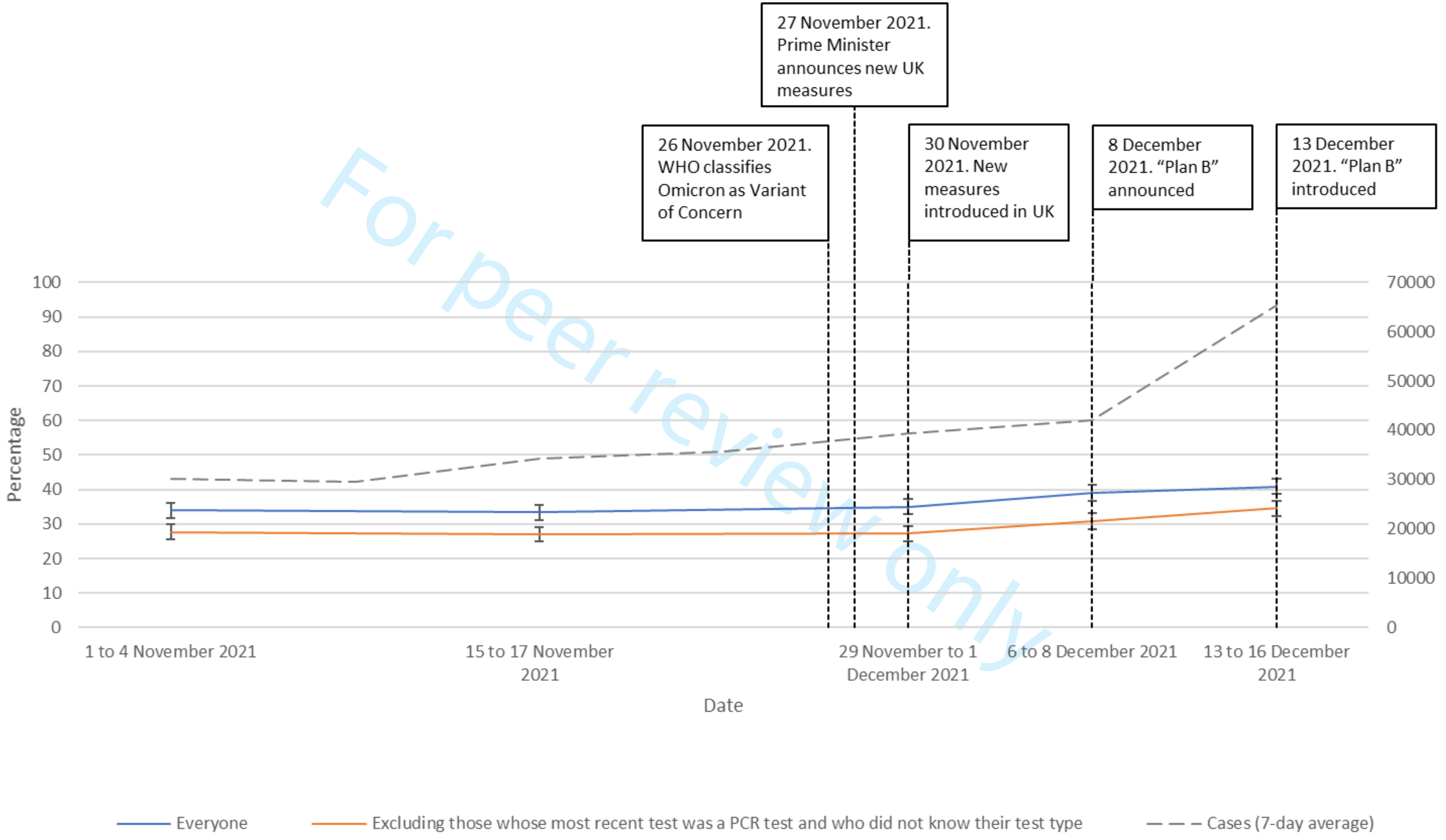

For peer review only

Supplementary materials

Figure 1. Timeline of announcements, data collection, and dates of self-reported behaviours. All dates 2021.

<table>
<tr><td>22-Nov</td><td></td><td></td><td rowspan="11">Wave 63 “past seven days” could cover</td><td></td><td></td></tr>
<tr><td>23-Nov</td><td></td><td></td><td></td><td></td></tr>
<tr><td>24-Nov</td><td>Omicron variant reported to WHO</td><td></td><td></td><td></td></tr>
<tr><td>25-Nov</td><td></td><td></td><td></td><td></td></tr>
<tr><td>26-Nov</td><td>Omicron designated a variant of concern</td><td></td><td></td><td></td></tr>
<tr><td>27-Nov</td><td>New measures announced
First UK cases</td><td></td><td></td><td></td></tr>
<tr><td>28-Nov</td><td></td><td></td><td></td><td></td></tr>
<tr><td>29-Nov</td><td></td><td rowspan="4">Wave 63</td><td rowspan="10">Wave 63.5 “past seven days” could cover</td><td></td></tr>
<tr><td>30-Nov</td><td>New measures take effect
Daily Mirror breaks ‘Partygate’</td><td></td></tr>
<tr><td>01-Dec</td><td></td><td></td></tr>
<tr><td>02-Dec</td><td></td><td></td></tr>
<tr><td>03-Dec</td><td>Omicron cases in the UK exceed 100</td><td></td><td></td><td></td></tr>
<tr><td>04-Dec</td><td></td><td></td><td></td><td></td></tr>
<tr><td>05-Dec</td><td></td><td></td><td></td><td></td></tr>
<tr><td>06-Dec</td><td></td><td rowspan="3">Wave 63.5</td><td></td><td rowspan="8">Wave 64 “past seven days” could cover</td></tr>
<tr><td>07-Dec</td><td>ITV reports on a video seeming to confirm ‘Partygate’</td><td></td></tr>
<tr><td>08-Dec</td><td>“Plan B” announced</td><td></td></tr>
<tr><td>09-Dec</td><td></td><td></td><td></td><td></td></tr>
<tr><td>10-Dec</td><td></td><td></td><td></td><td></td></tr>
<tr><td>11-Dec</td><td></td><td></td><td></td><td></td></tr>
<tr><td>12-Dec</td><td></td><td></td><td></td><td></td></tr>
<tr><td>13-Dec</td><td>“Plan B” mostly takes effect
First UK death from Omicron</td><td>Wave 64</td><td></td><td></td></tr>
</table>

14-Dec					
15-Dec	UK records its highest number of daily cases (78,610)				
16-Dec	UK records its highest number of daily cases (88,376)				

For peer review only

Table 1. Associations between out-of-home activities and socio-demographic factors, adjusting for all other socio-demographic factors. Bolding denotes significant findings (p<.002).

		Going out shopping (for groceries/pharmacy and other items) [block 1]				Attending the workplace [block 1]			
Attribute	Level	Wave 63.5 [a] aIRR for going out shopping (95% CI)	*p*	Wave 64 [b] aIRR for going out shopping (95% CI)	*p*	Wave 63.5 [c] aIRR for attending the workplace (95% CI)	*p*	Wave 64 [d] aIRR for attending the workplace (95% CI)	*p*
Region	East Midlands	Ref	-	Ref	-	Ref	-	Ref	-
	East of England	0.93 (0.72 to 1.21)	0.59	0.90 (0.70 to 1.15)	0.40	1.07 (0.53 to 2.16)	0.84	0.94 (0.52 to 1.70)	0.83
	London	1.10 (0.84 to 1.44)	0.48	1.09 (0.86 to 1.39)	0.46	0.93 (0.51 to 1.69)	0.81	1.15 (0.69 to 1.92)	0.60
	North East	1.01 (0.71 to 1.45)	0.94	0.96 (0.71 to 1.31)	0.81	0.83 (0.38 to 1.84)	0.65	1.07 (0.49 to 2.34)	0.86
	North West	1.08 (0.83 to 1.42)	0.57	1.06 (0.84 to 1.33)	0.63	1.17 (0.62 to 2.18)	0.63	0.91 (0.53 to 1.58)	0.75
	South East	0.99 (0.77 to 1.27)	0.93	0.94 (0.75 to 1.17)	0.57	0.96 (0.51 to 1.82)	0.90	1.14 (0.67 to 1.97)	0.63
	South West	0.98 (0.75 to 1.30)	0.91	1.05 (0.82 to 1.35)	0.68	1.50 (0.79 to 2.84)	0.22	1.54 (0.81 to 2.91)	0.18
	West Midlands	1.13 (0.86 to 1.49)	0.37	1.02 (0.80 to 1.31)	0.86	1.32 (0.71 to 2.46)	0.38	0.78 (0.44 to 1.39)	0.41
	Yorkshire and The Humber	1.37 (0.99 to 1.90)	0.06	0.89 (0.70 to 1.13)	0.33	1.24 (0.53 to 2.88)	0.62	0.67 (0.35 to 1.31)	0.24
	Overall	$\chi^2(8)=9.3$	0.32	$\chi^2(8)=6.6$	0.58	$\chi^2(8)=5.9$	0.66	$\chi^2(8)=7.9$	0.44
Sex	Male	Ref	-	Ref	-	Ref	-	Ref	-
	Female	0.83 (0.74 to 0.94)	0.002	0.94 (0.84 to 1.05)	0.25	0.72 (0.54 to 0.96)	0.03	0.72 (0.54 to 0.95)	0.02
Age (per decade)	Raw age	0.95 (0.91 to 0.99)	0.02	0.98 (0.94 to 1.01)	0.22	0.78 (0.65 to 0.93)	0.007	0.89 (0.77 to 1.03)	0.13
Age: quadratic $(\text{age-mean})^2$	-	1.0000 (0.9998 to 1.0002)	0.99	0.9999 (0.9997 to 1.0001)	0.37	0.9995 (0.9987 to 1.0003)	0.23	0.9997 (0.9990 to 1.0005)	0.48
Dependent child in household	None	Ref	-	Ref	-	Ref	-	Ref	-
	Child present	1.07 (0.93 to 1.23)	0.34	1.15 (1.01 to 1.31)	0.04	1.33 (0.98 to 1.81)	0.07	1.21 (0.91 to 1.61)	0.20
At high risk (self)	No	Ref	-	Ref	-	Ref	-	Ref	-
	Yes	0.88 (0.75 to 1.03)	0.10	0.96 (0.83 to 1.10)	0.53	0.99 (0.69 to 1.41)	0.94	0.83 (0.57 to 1.22)	0.35
Household member has chronic illness	No	Ref	-	Ref	-	Ref	-	Ref	-
	Yes	0.95 (0.80 to 1.13)	0.57	1.14 (0.98 to 1.33)	0.08	1.12 (0.68 to 1.84)	0.66	0.97 (0.61 to 1.55)	0.90
Employment status	Not working	Ref	-	Ref	-	-	-	-	-
	Working	1.07 (0.92 to 1.23)	0.37	0.97 (0.85 to 1.11)	0.65	-	-	-	-
Socio-economic grade	ABC1	Ref	-	Ref	-	Ref	-	Ref	-
	C2DE	1.06 (0.93 to 1.20)	0.38	0.98 (0.87 to 1.10)	0.74	0.70 (0.51 to 0.96)	0.03	0.85 (0.61 to 1.18)	0.32
Index of multiple deprivation	1st (least) to 4th quartile (most deprived)	0.95 (0.90 to 1.01)	0.10	0.95 (0.89 to 1.00)	0.05	0.97 (0.84 to 1.12)	0.71	0.84 (0.73 to 0.97)	0.02
Highest educational or professional qualification	Less than degree	Ref	-	Ref	-	Ref	-	Ref	-
	Degree or higher	0.96 (0.84 to 1.10)	0.54	1.02 (0.90 to 1.16)	0.74	0.83 (0.63 to 1.11)	0.22	0.64 (0.47 to 0.87)	0.004
Ethnicity	White British	Ref	-	Ref	-	Ref	-	Ref	-
	White other	1.21 (0.88 to 1.64)	0.24	0.76 (0.55 to 1.06)	0.11	1.36 (0.69 to 2.68)	0.38	0.97 (0.51 to 1.84)	0.92
	Black and minority ethnicity	0.87 (0.71 to 1.07)	0.20	1.06 (0.88 to 1.27)	0.55	1.03 (0.70 to 1.49)	0.90	0.98 (0.67 to 1.44)	0.92
	Overall	$\chi^2(2)=4.3$	0.12	$\chi^2(2)=3.9$	0.14	$\chi^2(2)=0.8$	0.68	$\chi^2(2)=0.0$	0.99

For peer review only

First language	Not English	Ref	-	Ref	-	Ref	-	Ref	-
	English	0.72 (0.55 to 0.93)	0.013	0.71 (0.55 to 0.92)	0.01	1.23 (0.74 to 2.04)	0.42	0.68 (0.41 to 1.12)	0.13
Ever had COVID-19	Think not	Ref	-	Ref	-	Ref	-	Ref	-
	Think yes	1.17 (1.02 to 1.34)	0.03	0.95 (0.84 to 1.09)	0.48	0.92 (0.68 to 1.25)	0.60	0.96 (0.71 to 1.30)	0.80
Vaccination status	Not vaccinated	Ref	-	Ref	-	Ref	-	Ref	-
	1 dose	1.19 (0.89 to 1.57)	0.24	1.16 (0.89 to 1.51)	0.27	1.37 (0.81 to 2.32)	0.24	1.47 (0.87 to 2.47)	0.15
	2 doses or more	0.98 (0.82 to 1.18)	0.85	0.80 (0.68 to 0.96)	0.01	0.95 (0.64 to 1.40)	0.78	1.10 (0.76 to 1.60)	0.62
	Overall	$\chi^2(2)=2.2$	0.34	$\chi^2(2)=12.5$	0.002	$\chi^2(2)=2.6$	0.27	$\chi^2(2)=2.1$	0.34
Financial hardship	Range 3 (least) to 15 (most)	1.01 (0.99 to 1.03)	0.21	**1.03 (1.02 to 1.05)**	**<0.001**	1.02 (0.97 to 1.07)	0.40	1.06 (1.01 to 1.10)	0.02

a) 1622 people were eligible for inclusion in analyses investigating going out shopping analyses. There were different amounts of missing data depending on variables included in the models, so n ranged between 1440 and 1491 for different models.

b) 1841 people were eligible for inclusion in analyses investigating going out shopping analyses. There were different amounts of missing data depending on variables included in the models, so n ranged between 1671 and 1713 for different models.

c) 374 people were eligible for inclusion in analyses investigating non-essential workplace attendance (sample limited to people who reported they could work entirely from home). Due to missing data, n included in analyses ranged between 349 and 354.

d) 410 people were eligible for inclusion in analyses investigating non-essential workplace attendance (sample limited to people who reported they could work entirely from home). Due to missing data, n included in analyses ranged between 379 and 389.

For peer review only

Table 2. Associations between highest risk social mixing and socio-demographic factors, adjusting for all other socio-demographic characteristics. Results reported are for block 1 of worry analyses. Bolding denotes significant findings (p<.002).

		Highest risk social mixing				Wearing a face covering in shops				Wearing a face covering in hospitality venues			
Attribute	Level	Wave 63.5 [a] aOR for engaging in highest risk social mixing (95% CI)	*p*	Wave 64 [b] aOR for engaging in highest risk social mixing (95% CI)	*p*	Wave 63.5 [c] aOR for wearing a face covering in shops (95% CI)	*p*	Wave 64 [d] aOR for wearing a face covering in shops (95% CI)	*p*	Wave 63.5 [e] aOR for wearing a face covering in hospitality venues (95% CI)	*p*	Wave 64 [f] aOR for wearing a face covering in hospitality venues (95% CI)	*p*
Region	East Midlands	Ref	-	Ref	-	Ref	-	Ref	-	Ref	-	Ref	-
	East of England	1.58 (0.99 to 2.54)	0.06	0.89 (0.57 to 1.39)	0.61	1.04 (0.54 to 2.02)	0.91	0.55 (0.26 to 1.18)	0.13	0.59 (0.29 to 1.24)	0.17	1.17 (0.60 to 2.25)	0.65
	London	1.36 (0.82 to 2.26)	0.23	0.94 (0.61 to 1.45)	0.77	1.12 (0.57 to 2.18)	0.75	0.48 (0.23 to 1.00)	0.05	0.52 (0.25 to 1.09)	0.08	0.91 (0.50 to 1.66)	0.76
	North East	1.52 (0.79 to 2.91)	0.21	1.31 (0.76 to 2.27)	0.33	0.99 (0.42 to 2.35)	0.99	0.46 (0.19 to 1.11)	0.08	0.47 (0.18 to 1.22)	0.12	2.04 (0.97 to 4.33)	0.06
	North West	1.25 (0.75 to 2.06)	0.39	1.17 (0.77 to 1.79)	0.45	0.86 (0.44 to 1.67)	0.65	**0.31 (0.15 to 0.62)**	**0.001**	0.44 (0.21 to 0.96)	0.04	0.67 (0.36 to 1.24)	0.20
	South East	1.59 (1.00 to 2.51)	0.05	1.17 (0.77 to 1.76)	0.46	0.93 (0.50 to 1.73)	0.81	0.49 (0.24 to 1.00)	0.05	0.51 (0.25 to 1.02)	0.06	1.28 (0.71 to 2.30)	0.42
	South West	1.34 (0.80 to 2.23)	0.27	1.03 (0.66 to 1.62)	0.89	1.02 (0.51 to 2.02)	0.96	0.44 (0.21 to 0.94)	0.03	0.65 (0.29 to 1.43)	0.28	1.27 (0.68 to 2.38)	0.45
	West Midlands	1.27 (0.77 to 2.11)	0.35	1.01 (0.65 to 1.59)	0.96	1.07 (0.54 to 2.11)	0.85	0.34 (0.16 to 0.71)	0.004	0.52 (0.24 to 1.10)	0.09	1.01 (0.52 to 1.97)	0.97
	Yorkshire and The Humber	1.34 (0.73 to 2.46)	0.35	1.21 (0.78 to 1.87)	0.40	1.21 (0.53 to 2.75)	0.65	0.33 (0.16 to 0.69)	0.003	0.26 (0.10 to 0.70)	0.008	1.22 (0.64 to 2.32)	0.54
	Overall	$\chi^2(8)=5.6$	0.69	$\chi^2(8)=5.0$	0.76	$\chi^2(8)=1.6$	0.99	$\chi^2(8)=15.2$	0.05	$\chi^2(8)=8.8$	0.36	$\chi^2(8)=12.3$	0.14
Sex	Male	Ref	-	Ref	-	Ref	-	Ref	-	Ref	-	Ref	-
	Female	1.40 (1.12 to 1.74)	0.003	1.35 (1.10 to 1.65)	0.003	**1.64 (1.22 to 2.21)**	**0.001**	1.37 (1.03 to 1.83)	0.03	1.34 (0.96 to 1.86)	0.08	1.46 (1.09 to 1.95)	0.01
Age (per decade)	Raw age	0.98 (0.90 to 1.06)	0.55	0.95 (0.88 to 1.02)	0.15	1.18 (1.05 to 1.32)	0.005	**1.22 (1.10 to 1.35)**	**<0.001**	1.03 (0.91 to 1.16)	0.66	1.06 (0.96 to 1.18)	0.24
Age: quadratic $(age\text{-}mean)^2$	-	1.0000 (0.9996 to 1.0004)	0.97	1.0002 (0.9999 to 1.0006)	0.20	1.0000 (0.9995 to 1.0006)	0.91	0.9998 (0.9993 to 1.0003)	0.40	0.9999 (0.9993 to 1.0005)	0.64	1.0006 (1.0001 to 1.0011)	0.02
Dependent child in household	None	Ref	-	Ref	-	Ref	-	Ref	-	Ref	-	Ref	-
	Child present	0.82 (0.63 to 1.06)	0.13	0.83 (0.65 to 1.05)	0.12	**0.56 (0.41 to 0.78)**	**0.001**	0.93 (0.68 to 1.28)	0.66	0.97 (0.67 to 1.41)	0.88	1.68 (1.19 to 2.37)	0.003
At high risk (self)	No	Ref	-	Ref	-	Ref	-	Ref	-	Ref	-	Ref	-
	Yes	0.90 (0.67 to 1.19)	0.45	0.77 (0.59 to 1.01)	0.05	1.14 (0.77 to 1.69)	0.50	0.80 (0.56 to 1.16)	0.24	1.32 (0.87 to 2.00)	0.19	1.34 (0.91 to 1.96)	0.13
	No	Ref	-	Ref	-	Ref	-	Ref	-	Ref	-	Ref	-

For peer review only

Household member has chronic illness	Yes	0.83 (0.61 to 1.13)	0.24	1.07 (0.81 to 1.41)	0.64	0.84 (0.55 to 1.27)	0.40	0.85 (0.58 to 1.25)	0.42	0.96 (0.60 to 1.55)	0.88	1.47 (0.97 to 2.22)	0.07
Employment status	Not working	Ref	-	Ref	-	Ref	-	Ref	-	Ref	-	Ref	-
	Working	0.76 (0.58 to 0.99)	0.04	0.77 (0.60 to 0.98)	0.04	0.98 (0.69 to 1.40)	0.92	1.01 (0.72 to 1.42)	0.96	0.63 (0.42 to 0.95)	0.03	0.96 (0.66 to 1.38)	0.81
Socio-economic grade‡	ABC1	Ref	-	Ref	-	Ref	-	Ref	-	Ref	-	Ref	-
	C2DE	0.89 (0.71 to 1.13)	0.34	0.79 (0.64 to 0.98)	0.03	0.76 (0.56 to 1.04)	0.09	0.74 (0.55 to 1.01)	0.06	0.84 (0.59 to 1.20)	0.34	0.88 (0.64 to 1.21)	0.43
Index of multiple deprivation	1st (least) to 4th quartile (most deprived)	1.02 (0.91 to 1.14)	0.71	1.03 (0.93 to 1.14)	0.54	1.01 (0.87 to 1.17)	0.93	1.07 (0.93 to 1.24)	0.34	0.99 (0.84 to 1.18)	0.95	1.04 (0.89 to 1.21)	0.61
Highest educational or professional qualification	Less than degree	Ref	-	Ref	-	Ref	-	Ref	-	Ref	-	Ref	-
	Degree or higher	1.05 (0.83 to 1.34)	0.68	1.05 (0.84 to 1.32)	0.66	1.16 (0.83 to 1.61)	0.38	1.21 (0.86 to 1.70)	0.27	1.03 (0.72 to 1.46)	0.88	1.40 (1.01 to 1.95)	0.04
Ethnicity	White British	Ref	-	Ref	-	Ref	-	Ref	-	Ref	-	Ref	-
	White other	1.16 (0.64 to 2.11)	0.63	0.99 (0.54 to 1.80)	0.96	1.17 (0.54 to 2.50)	0.69	0.84 (0.38 to 1.85)	0.67	0.52 (0.21 to 1.30)	0.16	1.57 (0.66 to 3.72)	0.31
	Black and minority ethnicity	0.69 (0.47 to 1.03)	0.07	0.81 (0.57 to 1.16)	0.25	1.39 (0.86 to 2.26)	0.18	1.65 (1.02 to 2.68)	0.04	1.49 (0.87 to 2.55)	0.14	1.40 (0.85 to 2.29)	0.19
	Overall	$\chi^2(2)$=4.4	0.11	$\chi^2(2)$=1.5	0.48	$\chi^2(2)$=1.8	0.41	$\chi^2(2)$=5.4	0.07	$\chi^2(2)$=5.4	0.07	$\chi^2(2)$=2.1	0.35
First language	Not English	Ref	-	Ref	-	Ref	-	Ref	-	Ref	-	Ref	-
	English	1.45 (0.86 to 2.47)	0.17	0.90 (0.55 to 1.49)	0.68	1.09 (0.58 to 2.02)	0.80	0.91 (0.46 to 1.78)	0.77	1.48 (0.72 to 3.03)	0.28	1.10 (0.56 to 2.16)	0.79
Ever had COVID-19	Think not	Ref	-	Ref	-	Ref	-	Ref	-	Ref	-	Ref	-
	Think yes	1.01 (0.78 to 1.31)	0.93	1.31 (1.03 to 1.66)	0.03	1.11 (0.80 to 1.54)	0.54	0.85 (0.62 to 1.18)	0.34	1.13 (0.78 to 1.64)	0.50	0.63 (0.45 to 0.87)	0.006
Vaccination status	Not vaccinated	Ref	-	Ref	-	Ref	-	Ref	-	Ref	-	Ref	-
	1 dose	0.72 (0.41 to 1.26)	0.25	0.61 (0.36 to 1.01)	0.06	2.62 (1.37 to 4.99)	0.003	**2.62 (1.48 to 4.66)**	**0.001**	**3.83 (1.86 to 7.88)**	**<0.001**	1.22 (0.63 to 2.39)	0.55
	2 doses or more	0.90 (0.64 to 1.26)	0.54	0.98 (0.71 to 1.35)	0.92	**2.31 (1.56 to 3.40)**	**<0.001**	**4.44 (3.05 to 6.48)**	**<0.001**	2.29 (1.35 to 3.87)	0.002	1.34 (0.84 to 2.15)	0.22
	Overall	$\chi^2(2)$=1.4	0.51	$\chi^2(2)$=4.3	0.11	**$\chi^2(2)$=19.4**	**<0.001**	**$\chi^2(2)$=60.1**	**<0.001**	**$\chi^2(2)$=14.9**	**0.001**	$\chi^2(2)$=1.5	0.48
Financial hardship	Range 3 (least) to 15 (most)	0.95 (0.92 to 0.98)	0.004	0.96 (0.92 to 0.99)	0.01	0.97 (0.93 to 1.02)	0.21	0.94 (0.9 to 0.99)	0.02	1.03 (0.97 to 1.08)	0.33	1.01 (0.96 to 1.06)	0.65

a) 1622 people were eligible for inclusion in highest risk social mixing analyses. There were different amounts of missing data depending on variables included in the models, so n ranged between 1439 and 1446 for different models.

b) 1841 people were eligible for inclusion in highest risk social mixing analyses. There were different amounts of missing data depending on variables included in the models, so n ranged between 1668 and 1689 for different models.

For peer review only

c) 1404 people were eligible for inclusion in wearing a face covering in shops analyses (sample limited to people who reported having been out shopping in the last week). There were different amounts of missing data depending on variables included in the models, so n ranged between 1247 and 1266 for different models.

d) 1600 people were eligible for inclusion in wearing a face covering in shops analyses (sample limited to people who reported having been out shopping in the last week). There were different amounts of missing data depending on variables included in the models, so n ranged between 1454 and 1475 for different models.

e) 789 people were eligible for inclusion in wearing a face covering in hospitality venues analyses (sample limited to people who reported having been out to hospitality venues in the last week). There were different amounts of missing data depending on variables included in the models, so n ranged between 700 and 713 for different models.

f) 894 people were eligible for inclusion in wearing a face covering in hospitality venues analyses (sample limited to people who reported having been out to hospitality venues in the last week). There were different amounts of missing data depending on variables included in the models, so n ranged between 817 and 829 for different models.

For peer review only

STROBE Statement—Checklist of items that should be included in reports of ***cross-sectional studies***

	Item No	Recommendation	Page No
**Title and abstract**	1	(*a*) Indicate the study's design with a commonly used term in the title or the abstract	1,2
		(*b*) Provide in the abstract an informative and balanced summary of what was done and what was found	2
**Introduction**			
Background/rationale	2	Explain the scientific background and rationale for the investigation being reported	4
Objectives	3	State specific objectives, including any prespecified hypotheses	4
**Methods**			
Study design	4	Present key elements of study design early in the paper	5
Setting	5	Describe the setting, locations, and relevant dates, including periods of recruitment, exposure, follow-up, and data collection	5
Participants	6	(*a*) Give the eligibility criteria, and the sources and methods of selection of participants	5
Variables	7	Clearly define all outcomes, exposures, predictors, potential confounders, and effect modifiers. Give diagnostic criteria, if applicable	5-7
Data sources/ measurement	8*	For each variable of interest, give sources of data and details of methods of assessment (measurement). Describe comparability of assessment methods if there is more than one group	*5-7*
Bias	9	Describe any efforts to address potential sources of bias	5-8
Study size	10	Explain how the study size was arrived at	5
Quantitative variables	11	Explain how quantitative variables were handled in the analyses. If applicable, describe which groupings were chosen and why	7-8
Statistical methods	12	(*a*) Describe all statistical methods, including those used to control for confounding	7-8
		(*b*) Describe any methods used to examine subgroups and interactions	N/A
		(*c*) Explain how missing data were addressed	7-8
		(*d*) If applicable, describe analytical methods taking account of sampling strategy	7-8
		(*e*) Describe any sensitivity analyses	N/A
**Results**			
Participants	13*	(a) Report numbers of individuals at each stage of study—eg numbers potentially eligible, examined for eligibility, confirmed eligible, included in the study, completing follow-up, and analysed	8
		(b) Give reasons for non-participation at each stage	N/A
		(c) Consider use of a flow diagram	Considered
Descriptive data	14*	(a) Give characteristics of study participants (eg demographic, clinical, social) and information on exposures and potential confounders	8
		(b) Indicate number of participants with missing data for each variable of interest	9-19
Outcome data	15*	Report numbers of outcome events or summary measures	9-19

For peer review only - http://bmjopen.bmj.com/site/about/guidelines.xhtml

For peer review only

Main results	16	(*a*) Give unadjusted estimates and, if applicable, confounder-adjusted estimates and their precision (eg, 95% confidence interval). Make clear which confounders were adjusted for and why they were included	9-19
		(*b*) Report category boundaries when continuous variables were categorized	Reported
		(*c*) If relevant, consider translating estimates of relative risk into absolute risk for a meaningful time period	Considered
Other analyses	17	Report other analyses done—eg analyses of subgroups and interactions, and sensitivity analyses	9-19
**Discussion**			
Key results	18	Summarise key results with reference to study objectives	20-21
Limitations	19	Discuss limitations of the study, taking into account sources of potential bias or imprecision. Discuss both direction and magnitude of any potential bias	21
Interpretation	20	Give a cautious overall interpretation of results considering objectives, limitations, multiplicity of analyses, results from similar studies, and other relevant evidence	20-21
Generalisability	21	Discuss the generalisability (external validity) of the study results	20-21
**Other information**			
Funding	22	Give the source of funding and the role of the funders for the present study and, if applicable, for the original study on which the present article is based	22

*Give information separately for exposed and unexposed groups.

Note: An Explanation and Elaboration article discusses each checklist item and gives methodological background and published examples of transparent reporting. The STROBE checklist is best used in conjunction with this article (freely available on the Web sites of PLoS Medicine at http://www.plosmedicine.org/, Annals of Internal Medicine at http://www.annals.org/, and Epidemiology at http://www.epidem.com/). Information on the STROBE Initiative is available at www.strobe-statement.org.

For peer review only

For peer review only - http://bmjopen.bmj.com/site/about/guidelines.xhtml
